# Maleic anhydride derived diphosphines: adaptable chelators for receptor-targeted ^99m^Tc, ^64^Cu and ^188^Re radiotracers

**DOI:** 10.1039/d5sc02110c

**Published:** 2025-08-26

**Authors:** Rachel E. Nuttall, Ingebjørg N. Hungnes, Truc T. Pham, Oliver W. L. Carter, Alex Rigby, Natasha Patel, Zilin Yu, Julie Cleaver, Jennifer D. Young, Gary J. R. Cook, Lefteris Livieratos, Jane Sosabowski, Hong Hoi Ting, Nicholas Vetter, Paul G. Pringle, Michelle T. Ma

**Affiliations:** a School of Bioengineering and Imaging Sciences, King's College London 4th Floor Lambeth Wing, St Thomas' Hospital London SE1 7EH UK rachel.nuttall@kcl.ac.uk michelle.ma@kcl.ac.uk; b Centre for Cancer Biomarkers and Biotherapeutics, Barts Cancer Institute, Queen Mary University of London, John Vane Science Centre Charterhouse Square London EC1M 6BQ UK; c Department of Nuclear Medicine, Guy's and St Thomas' Hospitals NHS Foundation Trust, Guy's Hospital London SE1 9RT UK; d Oncobeta GmbH 85748 Garching Munich Germany; e School of Chemistry, University of Bristol Cantock's Close Bristol BS8 1TS UK

## Abstract

The diagnostic imaging radionuclide, ^99m^Tc, used in Single Photon Emission Computed Tomography (SPECT), has extraordinary potential for enabling economical molecular receptor imaging in oncology, provided suitable chelators are available to enable kit-based radiolabelling. We report the development of two new bis(phosphino)maleic anhydrides, DP^An^ and DP^MEP^, that exhibit increased electron donor capacity and concomitant increased radiochemical yields compared to their first-generation diphosphine analogues. Both DP^An^ and DP^MEP^ can be reacted with a wide range of biological targeting vectors, including receptor-targeted peptides, carbohydrates, vitamins and small-molecule inhibitors. Exemplar diphosphine-peptide bioconjugates, DP^An^-PSMAt and DP^MEP^-PSMAt (which target the prostate-specific membrane antigen, PSMA), can be formulated into kits to enable near-quantitative, one-pot radiosynthesis of new ^99m^Tc radiotracers, *cis*/*trans*-[^99m^TcO_2_(DP^An^-PSMAt)_2_]^+^ and *cis*/*trans*-[^99m^TcO_2_(DP^MEP^-PSMAt)_2_]^+^, respectively. We demonstrate that the two exemplar ^99m^Tc radiotracers, *cis*/*trans*-[^99m^TcO_2_(DP^An^-PSMAt)_2_]^+^ and *cis*/*trans*-[^99m^TcO_2_(DP^MEP^-PSMAt)_2_]^+^, display favourable SPECT imaging properties in murine prostate cancer models, including high tumour uptake, fast clearance from circulation, excretion *via* a renal pathway and high metabolic stability. The same diphosphine-peptide bioconjugates can also be radiolabelled with the Positron Emission Tomography (PET) isotope, ^64^Cu, and the radiotherapeutic β^−^-emitting isotope, ^188^Re, in high radiochemical yields. The new DP^An^ and DP^MEP^ chelator platforms thus enable development of novel molecular imaging radiopharmaceuticals for ^99m^Tc SPECT, ^64^Cu PET and ^188^Re systemic radiotherapy.

## Abstract 2

Two new, versatile bis(phosphino)maleic anhydride platforms enable facile preparation of diphosphine bioconjugates that can be simply radiolabelled with ^99m^Tc for SPECT imaging, ^64^Cu for PET imaging and ^188^Re for systemic radiotherapy.

## Graphical abstract

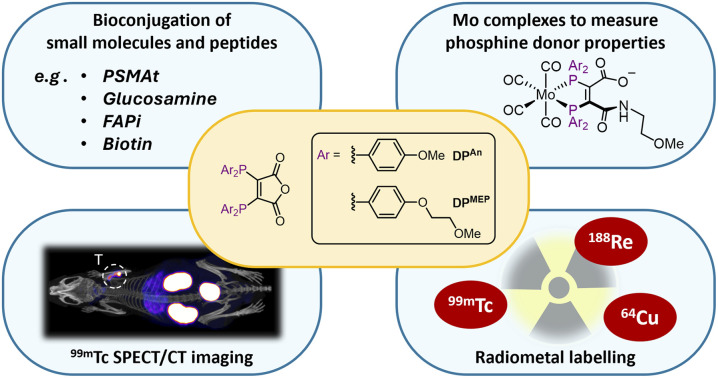

## Introduction

In oncology, receptor-targeted molecular imaging using Single Photon Emission Computed Tomography (SPECT) or γ-scintigraphy has utility in diagnosis, disease staging and clinical decision-making. One class of radiotracer used for this purpose consists of a peptide attached to a chelator, which in turn is complexed to a radioactive metal ion. Two of the most prominent SPECT radiotracers employed for this purpose are ^111^In-DTPA-octreotide and ^99m^Tc-EDDA/HYNIC-octreotide: both target the somatostatin receptor 2 that is overexpressed in neuroendocrine cancers.^1^ Whilst SPECT/γ-scintigraphy procedures with these radiotracers have been superseded in some healthcare settings by more sensitive Positron Emission Tomography (PET) imaging coupled with the PET radiotracer ^68^Ga-DOTA-octreotate,^2^ they are still widely used in many clinics where PET is not available.

There are several factors that have led to the prevalence of SPECT/γ-scintigraphy imaging procedures. First, worldwide, there is simply more SPECT and γ-scintigraphy infrastructure than PET infrastructure, including in lower and middle income countries.^3–6^ Second, ^99m^Tc (*t*_½_ = 6 h; 90% γ, 140 keV) is widely distributed and available from bench-top ^99^Mo/^99m^Tc generators in the form of ^99m^TcO_4_^−^ in aqueous saline solution.^5,6^ Third, ^99m^Tc radiotracers, which are routinely used in 30–40 million procedures globally each year, are produced using simple one- or two-step kits in near-quantitative radiochemical yields.^5–7^

Kits for the preparation of ^99m^Tc radiopharmaceuticals typically contain buffering salts, a reducing agent to reduce ^99m^Tc^VII^O_4_^−^, a chelator derivative that ultimately complexes the ^99m^Tc metal ion to form the desired radiopharmaceutical, and other reagents including weak chelators, which serve to stabilise ^99m^Tc intermediates.^5,7^ The majority of ^99m^Tc radiopharmaceuticals are for imaging perfusion or anatomical processes.^5,7^ Molecular imaging using ^99m^Tc-EDDA/HYNIC-octreotide is not as prevalent, in part because of the low incidence of neuroendocrine cancer.

However, recognising the utility and availability of molecular SPECT/γ-scintigraphy infrastructure, in recent years, several new ^99m^Tc-labelled peptides have been clinically evaluated for receptor-targeted molecular imaging of the prostate-specific membrane antigen (PSMA) overexpressed in prostate cancer. These include ^99m^Tc-MIP-1404 (also known as ^99m^Tc-Trofolastat),^8,9^^99m^Tc-PSMA-I&S^10,11^ and ^99m^Tc-EDDA/HYNIC-iPSMA,^12,13^ which have been shown to be viable alternatives to efficacious PET diagnostic agents that similarly target PSMA.

There is an additional incentive for development of ^99m^Tc radiotracers: in some cases, chemically analogous Re complexes are accessible, providing access to pairs of “theranostic” ^99m^Tc and ^188^Re radiopharmaceuticals for diagnosis and therapy, respectively. The rhenium radionuclide, ^188^Re (*t*_½_ = 17 h; 100% β^−^, *E*_max_ = 2.12 MeV; 15% γ, 155 keV), which can also be produced from a bench-top generator like ^99m^Tc, emits cytotoxic β^−^ particles. ^188^Re radiopharmaceuticals can be effective therapeutics. For example, the lipophilic ^188^Re-labelled radiopharmaceutical ^188^Re-lipiodol is not only clinically efficacious for treatment of inoperable liver cancer,^14^ but is also economically viable in lower and middle income countries where supplies of other β^−^-emitting radiopharmaceuticals are limited due to economical and/or geographical barriers.^15,16^ Indeed, a newly developed pair of ^99m^Tc/^188^Re-labelled PSMA-GCK01 theranostic agents has shown favourable properties in preclinical and initial first-in-human studies,^17^ demonstrating the feasibility of molecular imaging and therapeutic ^99m^Tc/^188^Re pairs.

**Fig. 1:**
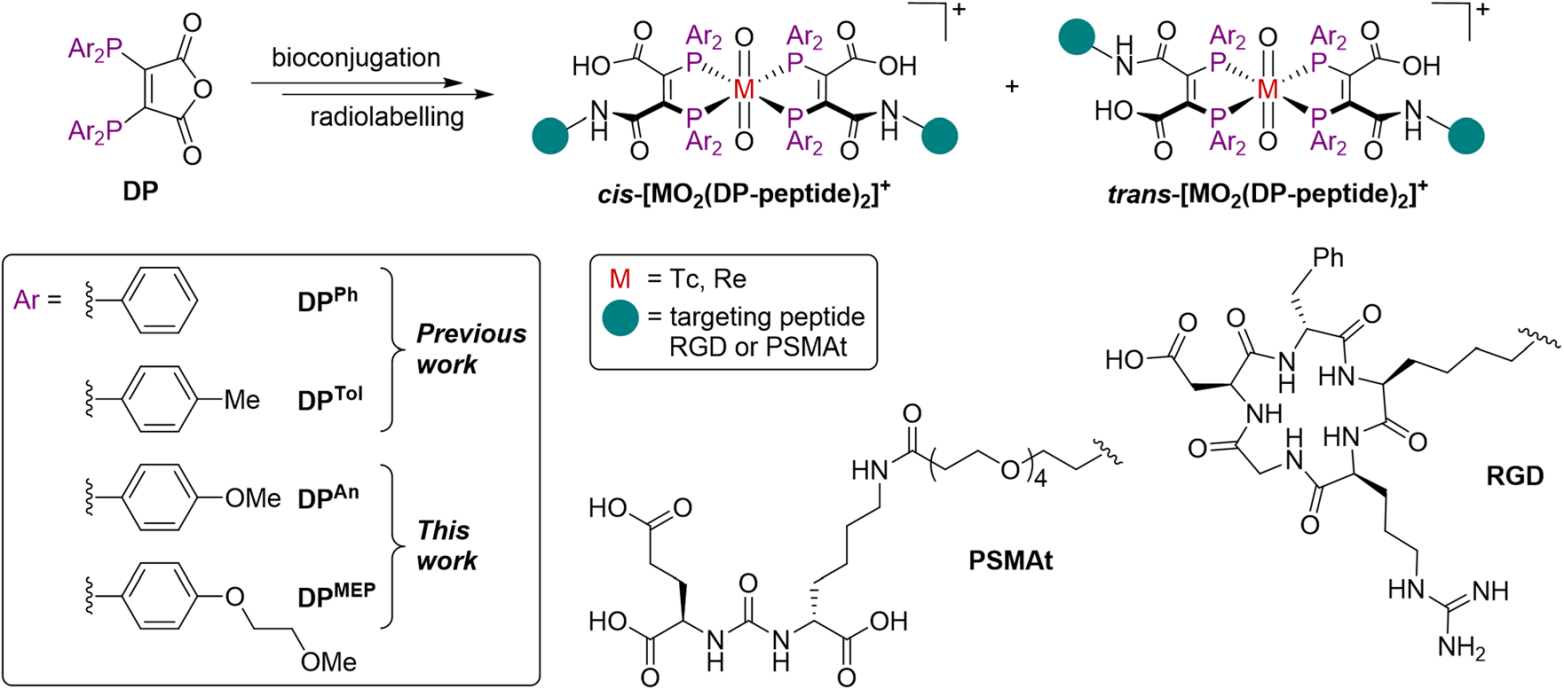
Structures of *cis*/*trans*-[MO_2_(DP-peptide)_2_]^+^ complexes, where M = Tc or Re.^18–20^

We have recently explored diphosphine derivatives as potential platforms for radiolabelling receptor-targeted biomolecules.^18–21^ These diphosphines include 2,3-bis(diphenylphosphino)maleic anhydride (DP^Ph^)^22,23^ and 2,3-bis(di-*p*-tolylphosphino)maleic anhydride (DP^Tol^) (Fig. 1) which react with the primary amine groups of peptides to furnish diphosphine-peptide (DP-peptide) conjugates.^18–20^††DP^Ph^ and DP^Tol^ and their derivatives have alternatively been abbreviated to “DP1” and “DP2”, respectively in our prior report in a medical journal.^20^ The DP-peptide derivatives coordinate [TcO_2_]^+^ or [ReO_2_]^+^ motifs to yield complexes of the type *cis*/*trans*-[MO_2_(DP-peptide)_2_]^+^ (M = Tc, Re); radiolabelled ^99m^Tc and ^188^Re isotopologues are also synthetically accessible. We have also demonstrated that the resulting ^99m^Tc and ^188^Re radiotracers retain affinity for their cognate target receptors *in vitro* and *in vivo*, and have favourable biodistribution pathways including rapid clearance from the bloodstream *via* a renal pathway.^18,20^ We note that these derivatives also coordinate the PET imaging isotope, ^64^Cu (*t*_½_ = 12.7 h; 18% β^+^, *E*_max_ = 653 keV), rapidly at room temperature, furnishing radiotracers of formula [Cu(DP-peptide)_2_]^+^ that show high stability in serum.^19,22,23^

However, DP-peptide derivatives of DP^Ph^ do not provide ^99m^Tc radiotracers in sufficiently high radiochemical yield to enable preparation using a one-step kit without subsequent purification to remove unreacted ^99m^Tc precursors.^19^ Furthermore, radiochemical yields of ^188^Re derivatives are relatively low (<50%).^20^DP-peptide derivatives of DP^Tol^ (which contains *p*-tolyl substituents in place of phenyl groups) have shown increased electron donor capacity and concomitant increased radiochemical yields compared to DP^Ph^ derivatives.^19^ However, even with the improved reactivity of DP^Tol^, we have not been able to obtain radiochemical yields of [^99m^TcO_2_(DP-peptide)_2_]^+^ compounds above ∼85–90%. This is a barrier to clinical translation.

To address this, we have synthesised two novel diphosphine maleic anhydrides, DP^An^ and DP^MEP^ (Fig. 1), which possess either *p*-anisyl or *p*-(2-methoxyethoxy)phenyl groups on the phosphines, respectively. Bioconjugates of these diphosphine maleic anhydride platforms enable near-quantitative radiochemical syntheses of ^99m^Tc radiotracers. In our most comprehensive report yet, we have explored the scope of possible bioconjugation reactions with both DP^An^ and DP^MEP^ using a range of biological targeting vectors. We have attached DP^An^ and DP^MEP^ to a PSMA-targeted dipeptide (PSMAt), enabling comparison of DP^An^-PSMAt and DP^MEP^-PSMAt with our prior conjugate, DP^Ph^-PSMAt, including formation of ^99m^Tc, ^188^Re and ^64^Cu complexes. Lastly, our novel ^99m^Tc radiotracers have been assessed in murine models of prostate cancer using SPECT/CT imaging. We have therefore demonstrated the full utility of our novel and improved DP^An^ and DP^MEP^ platforms for enabling economical and accessible production of pairs of receptor-targeted theranostic radiotracers.

## Experimental

Details of experimental procedures are included in the SI. All animal experiments and procedures were ethically reviewed and approved by the Animal Welfare & Ethical Review Boards at either King's College London or Barts Cancer Institute. All animal experiments and procedures were then carried out in accordance with approvals from these committees and review boards, alongside the Animals (Scientific Procedures) Act 1986 UK Home Office regulations governing animal experimentation.

## Results

### Synthesis of DP^An^ and DP^MEP^ and their PSMAt conjugates

**Scheme 1:**
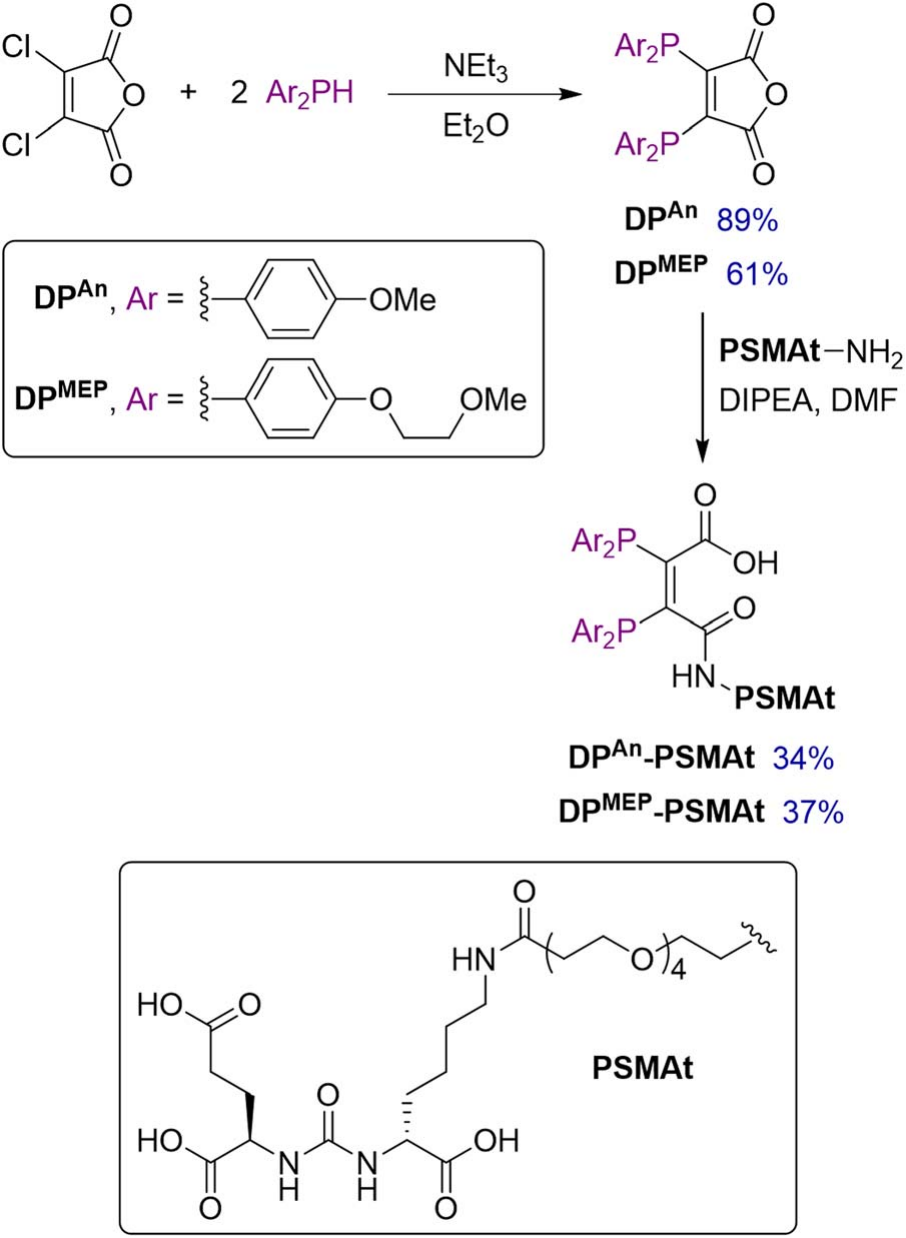
Synthesis of bis(phosphino)maleic anhydrides, DP^An^ and DP^MEP^.

Two novel bis(phosphino)maleic anhydrides were synthesised bearing either *p*-anisyl (DP^An^) or *p*-(2-methoxyethoxy)phenyl (DP^MEP^) substituents. By reaction of the required Ar_2_PH with 2,3-dichloromaleic anhydride in the presence of triethylamine base, DP^An^ and DP^MEP^ were synthesised in 89% and 61% yield, respectively (Scheme 1). The syntheses of all precursors, including both diarylphosphines, are described in the SI.

Next, both diphosphines, DP^An^ and DP^MEP^, were conjugated to a PSMA-targeting dipeptide, PSMAt-NH_2_. Using the pendant primary amine in PSMAt-NH_2_ under mild basic conditions, the maleic anhydrides readily ring-opened to form diphosphine bioconjugates, DP^An^-PSMAt and DP^MEP^-PSMAt in 34% and 37% isolated yield, respectively (Scheme 1). As previously reported for this type of ligand,^18,19^DP^An^-PSMAt and DP^MEP^-PSMAt were stable in the solid state; however, they both slowly oxidised in solution under atmospheric conditions. Under acidic conditions, a reverse reaction occurred to reform DP^An/MEP^ and PSMAt-NH_2_.

### Bioconjugation reactions of DP^An^ and DP^MEP^ with bioactive small molecules and peptides

**Fig. 2:**
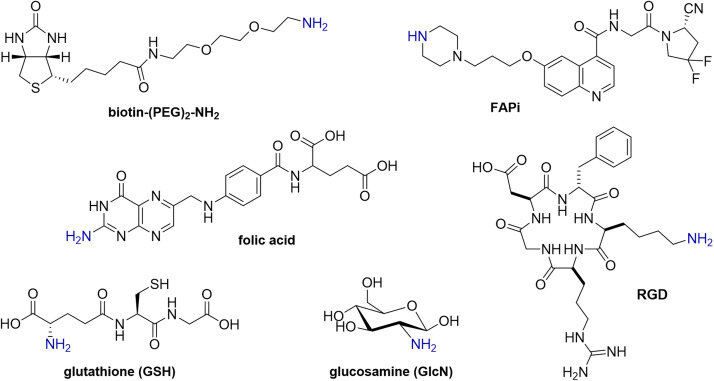
Structures of R–NH_2_ and RR′–NH amines.

To investigate the versatility of this DP platform, the reaction of each of DP^An^ and DP^MEP^ with a range of biologically active amines (Fig. 2) was assessed, including:

• Glucosamine (GlcN), a monosaccharide, as an exemplar carbohydrate;

• Biotin, a vitamin, which binds strongly to streptavidin protein; the biotin/streptavidin pair is used for purification and detection in biotechnology applications;^24,25^

• Folic acid, a vitamin which targets the folate receptor, overexpressed in ovarian, lung, breast and kidney cancers;^26,27^

• RGD, a cyclic pentapeptide, which targets the α_v_β_3_-integrin receptor overexpressed in inflammation, neovasculature and many cancers;^28^

• Glutathione (GSH), a thiol-containing antioxidant tripeptide;

• FAPi, a small molecule receptor-targeted inhibitor, which targets the fibroblast activation protein of fibroblastic cells, associated with hard-to-treat cancers (*e.g.* pancreatic, lung, ovarian cancers).^29^ Notably, the FAPi motif is a secondary amine (R_2_NH), whereas all other amine derivatives above contain primary amines (RNH_2_).

The crude reaction mixtures were analysed by LC-MS (Table 1, see SI for full chromatograms). All reactions underwent >95% consumption of the respective DP and/or amine (except for folic acid which showed no reaction). The lack of reactivity of folic acid with either DP^An^ or DP^MEP^ is presumably due to the low nucleophilicity of the primary amine on the pteridine ring. While both reactions with GSH showed full consumption of DP^An/MEP^, multiple species were detected by LC-MS. Analysis by ^31^P{^1^H} NMR spectroscopy indicated that although the desired amide derivative was formed, additional reactions also occurred, which we attribute to the reactive thiol group of GSH (see SI). When 1-propanethiol was separately reacted with DP^An^, multiple ^31^P{^1^H} NMR signals with similar chemical shifts were observed. In short, both new DP^An^ and DP^MEP^ compounds react with thiols to give multiple products: consideration should be taken when a biomolecule with a free thiol group is reacted with these diphosphine compounds.

**Table 1:** Summary of DP-amine conjugations

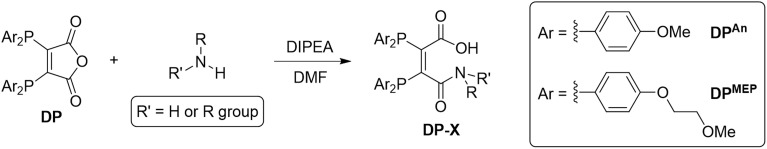						
Entry	DP	R–NH_2_ or RR′–NH^a^	DP-X	Expected *m*/*z* for [M + H]^+^	Observed *m*/*z*	Conversion^b^
1	DP^An^	Biotin-(PEG)_2_-NH_2_	DP^An^-biotin	961.3	961.7	>95%
2		FAPi	DP^An^-FAPi	1073.4	1072.9	>95%
3		Folic acid	DP^An^-folic acid	1028.3	—	NR^c^
4		Glucosamine (GlcN)	DP^An^-GlcN	766.2	766.3	>95%
5		Glutathione (GSH)	DP^An^-GSH	894.2	894.4	∼83%^d^
6		RGD	DP^An^-RGD	1190.5	1190.8	>95%
7	DP^MEP^	Biotin-(PEG)_2_-NH_2_	DP^MEP^-biotin	1138.4	1138.0	>95%
8		FAPi	DP^MEP^-FAPi	1249.5	1249.8	>95%
9		Folic acid	DP^MEP^-folic acid	1204.4	—	NR^c^
10		Glucosamine (GlcN)	DP^MEP^-GlcN	942.3	942.6	>95%
11		Glutathione (GSH)	DP^MEP^-GSH	1070.3	1071.1	∼86%^d^
12		RGD	DP^MEP^-RGD	1366.6	1366.9	>95%

a RR′–NH only applies to the reaction with FAPi, which contains a secondary amine.

b Approximate conversion was calculated using the UV signal (280 nm) in HPLC chromatogram(s). See SI for full chromatograms.

c NR = no reaction.

d Mixture of products.

### Evaluating the donor properties of DP^An^, DP^MEP^ and derivatives: IR spectra of Mo complexes

The IR stretching frequencies of CO ligands (*ν*_CO_) in metal complexes can be used to assess the binding properties of ligands, and complexes of the type *cis*-[Mo(CO)_4_L_2_] have previously been utilised for this purpose.^30^ Strong σ-donors, such as phosphines, increase π-back-bonding from Mo to CO ligands, resulting in lower CO stretching frequencies.

**Scheme 2:**
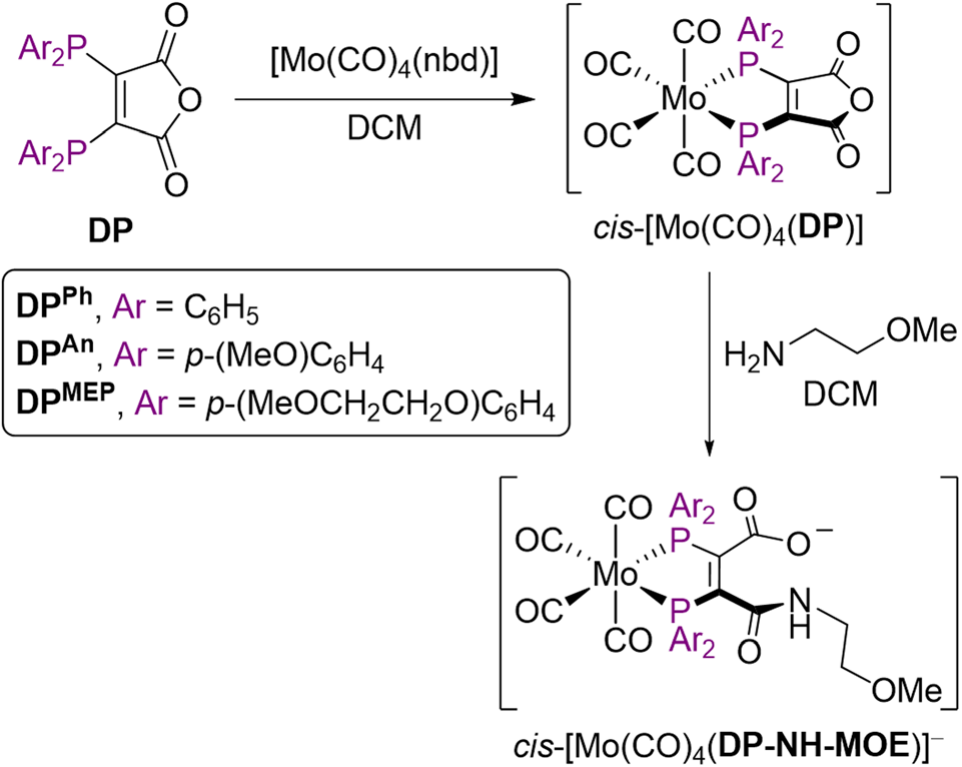
Mo(0) complexation of DP ligands to give *cis*-[Mo(CO)_4_(DP)] and subsequent ring-opening to give *cis*-[Mo(CO)_4_(DP-NH-MOE)]^−^.

Ligands DP^An^ and DP^MEP^, alongside the progenitor DP^Ph^ (for comparison) were reacted with *cis*-[Mo(CO)_4_(nbd)] to give *cis*-[Mo(CO)_4_(DP)] complexes (Scheme 2). Additionally, each of these complexes was reacted with (2-methoxyethyl)amine (MOE-NH_2_) to yield ring-opened compounds of the type [Mo(CO)_4_(DP-NH-MOE)]^−^,^19^ which all contained an amide bond, to model DP-peptide conjugate species (Scheme 2). These complexes were isolated as salts of MOE-NH_3_^+^.

The IR *ν*_CO_ data for these *cis*-[Mo(CO)_4_L_2_] complexes (alongside ^31^P{^1^H} NMR and selected ^13^C{^1^H} NMR data) are shown in Table 2. There was a decrease in *ν*_CO_ in the order [Mo(CO)_4_(nbd)] > [Mo(CO)_4_(DP^Ph^)] > [Mo(CO)_4_(DP^An^)] ∼ [Mo(CO)_4_(DP^MEP^)] > [Mo(CO)_4_(DP^Ph^-NH-MOE)]^−^ > [Mo(CO)_4_(DP^An^-NH-MOE)]^−^ > [Mo(CO)_4_(DP^MEP^-NH-MOE)]^−^. Importantly, complexes of DP^An^ and DP^MEP^ and their amide conjugates exhibited lower *ν*_CO_ values compared with analogous DP^Ph^ complexes, indicating that the new DP^An^ and DP^MEP^ ligands possess increased electron donor capacities compared to DP^Ph^ derivatives.

**Table 2:** Spectroscopic data for Mo complexes of DP^Ph^, DP^An^ and DP^MEP^ ligands and their derivatives

Compound	*v*_CO_ a (cm^−1^)	^31^P{^1^H} NMR (ppm)	^13^C{^1^H} NMR (ppm) of M–CO ligands^d^
DP^Ph^	—	−20.5 (s)^b^	—
DP^An^	—	−22.3 (s)^b^	—
DP^MEP^	—	−22.4 (s)^b^	—
[Mo(CO)_4_(nbd)]	2041, 1951, 1888	—	—
[Mo(CO)_4_(DP^Ph^)]	2031, ∼1939, 1920	51.7 (s)^b^	215.2 (m, CO_eq_), 208.9 (t, ^2^*J*_P,C_ = 8.5 Hz, CO_ax_)^19^
[Mo(CO)_4_(DP^An^)]	2028, ∼1932, 1916	49.0 (s)^b^	215.9–215.5 (m, CO_eq_), 209.1 (t, ^2^*J*_P,C_ = 8.6 Hz, CO_ax_)
[Mo(CO)_4_(DP^MEP^)]	2028, ∼1932, 1915	48.9 (s)^b^	215.8–215.4 (m, CO_eq_), 209.0 (t, ^2^*J*_P,C_ = 8.6 Hz, CO_ax_)
[MOE-NH_3_][Mo(CO)_4_(DP^Ph^-NH-MOE)]	2024, ∼1931, 1902	72.5 (d, *J* = 3.3 Hz), 70.2 (d, *J* = 3.3 Hz)^c^	217.9 (m, CO_eq_), 210.0 (t, ^2^*J*_P,C_ = 8.6 Hz, CO_ax_)^19^
[MOE-NH_3_][Mo(CO)_4_(DP^An^-NH-MOE)]	2023, ∼1928, 1899	69.3 (s), 67.4 (s)^c^	218.0–217.5 (m, CO_eq_), 209.7 (t, ^2^*J*_P,C_ = 8.5 Hz, CO_ax_)
[MOE-NH_3_][Mo(CO)_4_(DP^MEP^-NH-MOE)]	2021, 1927, 1897	69.1 (d, *J* = 2.7 Hz), 66.4 (d, *J* = 2.7 Hz)^c^	218.3–217.8 (m, CO_eq_), 209.8 (t, ^2^*J*_P,C_ = 8.5 Hz, CO_ax_)

a CH_2_Cl_2_, 1 mg mL^−1^, “∼” denotes shoulder peak.

b 162 MHz, CDCl_3_.

c 162 MHz, CD_2_Cl_2_.

d 126 MHz CD_2_Cl_2_.

The *ν*_CO_ stretches were all lower for the amide conjugates, [Mo(CO)_4_(DP^X^-NH-MOE)]^−^ (X = Ph, An, MEP), compared with the precursor species, [Mo(CO)_4_(DP^X^)] (X = Ph, An, MEP), indicating that DP-NHR ligands are significantly better electron donating ligands than the highly electron withdrawing bis(phosphino)maleic anhydride precursor ligands.

### ^99m^Tc and ^188^Re radiolabelling using generator-produced eluate

We next elected to study the reactions of DP^An^-PSMAt and DP^MEP^-PSMAt derivatives with generator-produced ^99m^Tc and ^188^Re. For this, the DP^An^-PSMAt and DP^MEP^-PSMAt conjugates were incorporated into lyophilised kits containing all components required for labelling: reducing agent (SnCl_2_), stabilising chelator (sodium tartrate) and buffer (sodium bicarbonate) (Table S1).

**Fig. 3:**
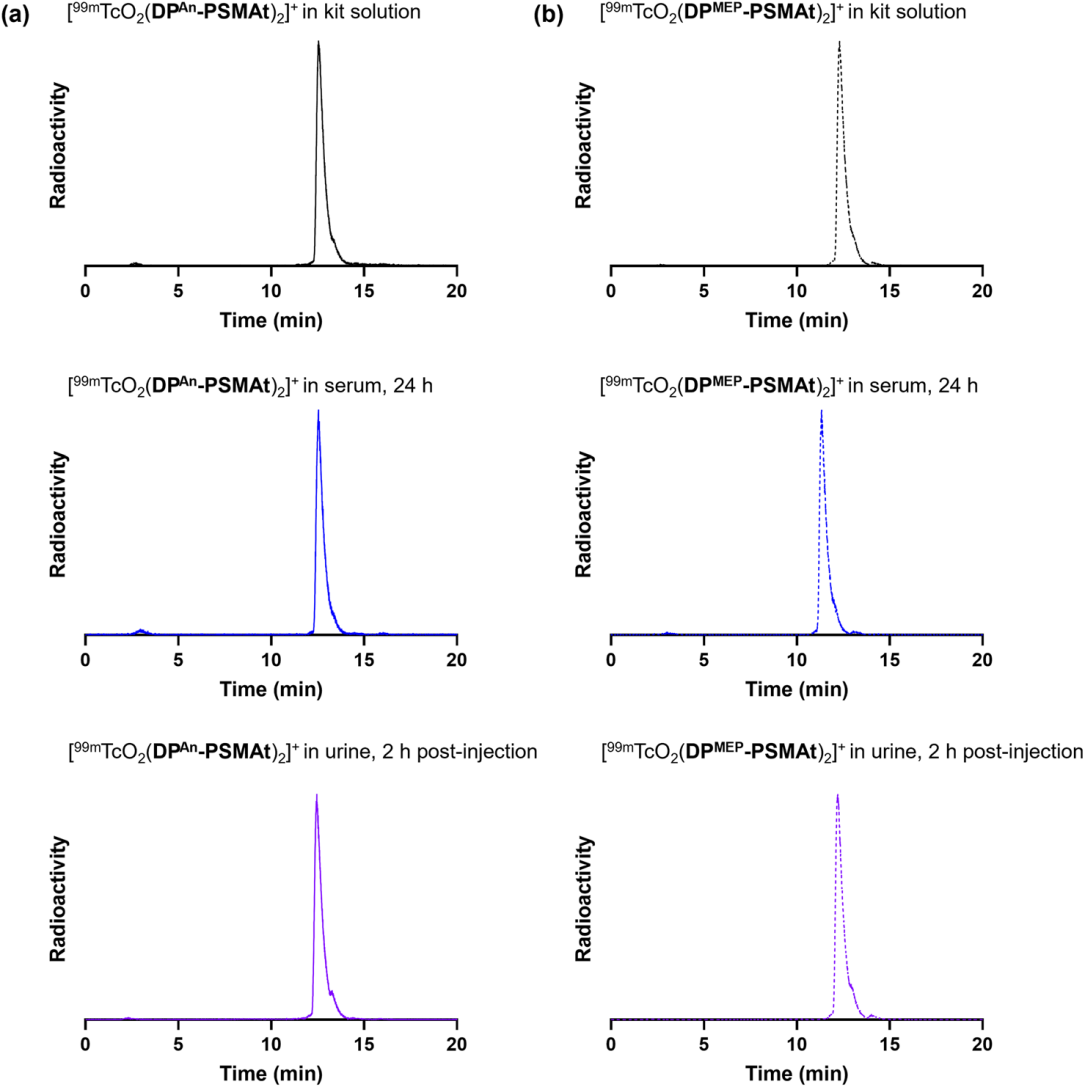
Radiochromatograms of reactions containing (a) [^99m^TcO_2_(DP^An^-PSMAt)_2_]^+^ and (b) [^99m^TcO_2_(DP^MEP^-PSMAt)_2_]^+^, including radiochromatograms of ^99m^Tc samples after either incubation in human serum at 37 °C, or after administration to a mouse, followed by collection of urine 2 hours post-administration. Radio-HPLC analyses of serum and urine samples indicate that ^99m^Tc radiotracers possess high stability in the presence of serum proteins and *in vivo*.

For ^99m^Tc radiosyntheses, the kits were reconstituted using generator produced ^99m^TcO_4_^−^ (200 MBq) in saline solution (500 μL) alongside an analogous DP^Ph^-PSMAt kit for comparison. The mixtures were then heated at 100 °C for 10 min before radio-iTLC and radio-HPLC analyses. Both [^99m^TcO_2_(DP^An^-PSMAt)_2_]^+^ and [^99m^TcO_2_(DP^MEP^-PSMAt)_2_]^+^ were consistently formed in ≥95% RCY (Table 3), whilst side-by-side radiolabelling reactions of DP^Ph^-PSMAt yielded <80% RCY. The major radiochemical impurity in these reactions was ^99m^Tc colloidal species. Analytical reverse-phase radio-HPLC chromatograms of reactions containing either [^99m^TcO_2_(DP^An^-PSMAt)_2_]^+^ or [^99m^TcO_2_(DP^MEP^-PSMAt)_2_]^+^ showed only one major radioactive species, which we attributed to a mixture of *cis*- and *trans*-[^99m^TcO_2_(DP^An^-PSMAt)_2_]^+^ or *cis*- and *trans*-[^99m^TcO_2_(DP^MEP^-PSMAt)_2_]^+^ (Fig. 3). Under these HPLC conditions, *cis* and *trans* isomers were not resolved, however using alternative HPLC conditions (*vide infra*), *cis* and *trans* isomers for [^99m^TcO_2_(DP^An^-PSMAt)_2_]^+^ could be discerned (see Fig. 5).

**Table 3:** RCYs of [^99m^TcO_2_(DP-PSMAt)_2_]^+^ and [^188^ReO_2_(DP-PSMAt)_2_]^+^ complexes

Complex	RCY (%)	Mean difference compared to [^99m^TcO_2_(DP^Ph^-PSMAt)_2_]^+^
[^99m^TcO_2_(DP^Ph^-PSMAt)_2_]^+^	76.9 ± 1.9 (*n* = 4)	Comparator
[^99m^TcO_2_(DP^An^-PSMAt)_2_]^+^	95.1 ± 0.7 (*n* = 6)	Mean difference = 18.2, 95% confidence interval = 15.3 to 21.1%, *p* = 0.0001
[^99m^TcO_2_(DP^MEP^-PSMAt)_2_]^+^	95.3 ± 1.3 (*n* = 7)	Mean difference = 18.9, 95% confidence interval = 15.9 to 21.8%, *p* = 0.0002
[^188^ReO_2_(DP^An^-PSMAt)_2_]^+^	84.7 ± 7.1% (*n* = 4)	—
[^188^ReO_2_(DP^MEP^-PSMAt)_2_]^+^	83.1 ± 6.8% (*n* = 4)	—

**Fig. 4:**
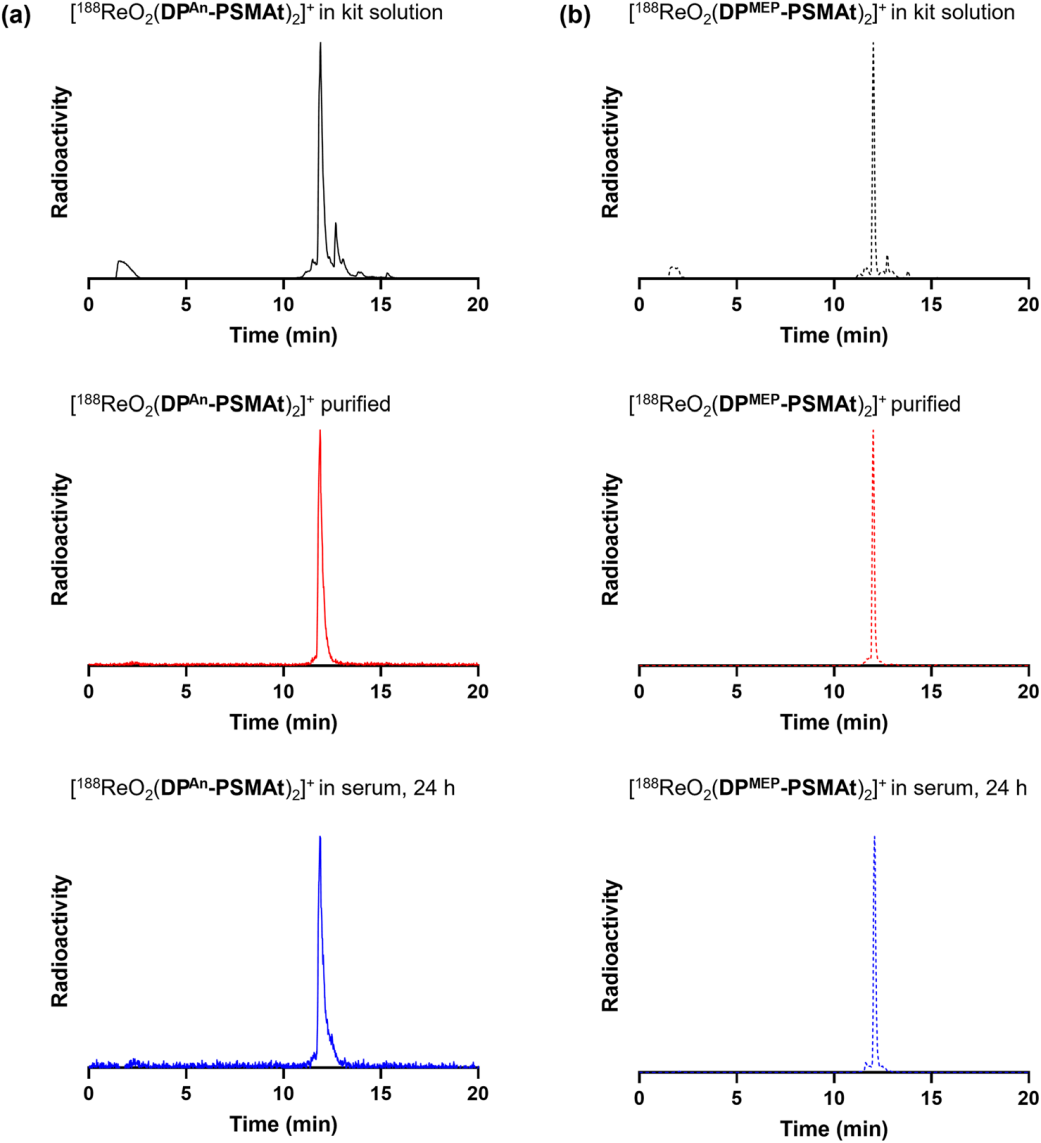
Radiochromatograms of reactions containing (a) [^188^ReO_2_(DP^An^-PSMAt)_2_]^+^ and (b) [^188^ReO_2_(DP^MEP^-PSMAt)_2_]^+^, including radiochromatograms of ^188^Re-labelled samples after purification and then incubation in human serum at 37 °C. Radio-HPLC analyses of serum samples indicate that ^188^Re radiotracers possess high stability in the presence of serum proteins.

For ^188^Re radiosyntheses, generator-produced ^188^ReO_4_^−^ was first reduced to a ^188^Re^V^-citrate precursor, using a mixture of sodium citrate and SnCl_2_, as previously described.^31,32^ Then, aqueous solutions of ^188^Re^V^-citrate (49–130 MBq, 85 μL) were added to the contents of two kits containing DP^An^-PSMAt or DP^MEP^-PSMAt (Table S1), and the mixtures heated to 90 °C for 30 min. The radiochemical yield of [^188^ReO_2_(DP^An^-PSMAt)_2_]^+^ measured 84.7% ± 7.1% (*n* = 4), and [^188^ReO_2_(DP^MEP^-PSMAt)_2_]^+^ measured 83.1% ± 6.8% (*n* = 4). Analytical reverse-phase radio-HPLC chromatograms (Fig. 4) showed that the reaction products, [^188^ReO_2_(DP^An^-PSMAt)_2_]^+^ and [^188^ReO_2_(DP^MEP^-PSMAt)_2_]^+^, formed one major radiochemical species, with the species observed at 2–3 min attributed to unreacted ^188^ReO_4_^−^/^188^Re^V^-citrate precursor(s). Signals attributed to the desired ^188^Re-radiolabelled products were broadened at the baseline, and it is possible that there are minor ^188^Re-radiolabelled side products present in this final kit-based reaction solution.

The log *D*_OCT/PBS_ of the new ^99m^Tc and ^188^Re radiotracers was measured (Table 4), with radiotracers isolated from unreacted ^99m^Tc/^188^Re precursor where required, prior to partition coefficient studies. The log *D*_OCT/PBS_ values of all four new radiotracers were less than −3.0, indicating that all are highly hydrophilic.

**Table 4:** Log *D*_OCT/PBS_ values of [MO_2_(DP-PSMAt)_2_]^+^ complexes, where M = ^99m^Tc or ^188^Re

	log *D*_OCT/PBS_	
	^99m^Tc	^188^Re
[MO_2_(DP^An^-PSMAt)_2_]^+^	−3.22 ± 0.04	−3.47 ± 0.05
[MO_2_(DP^MEP^-PSMAt)_2_]^+^	−3.35 ± 0.12	−3.91 ± 0.10

### ^99g^Tc radiolabelling

To verify that DP^An^-PSMAt and DP^MEP^-PSMAt form analogous complexes, the long-lived Tc radionuclide, ^99g^Tc (*t*_½_ = 2.1 × 10^5^ years), was utilised.

**Fig. 5:**
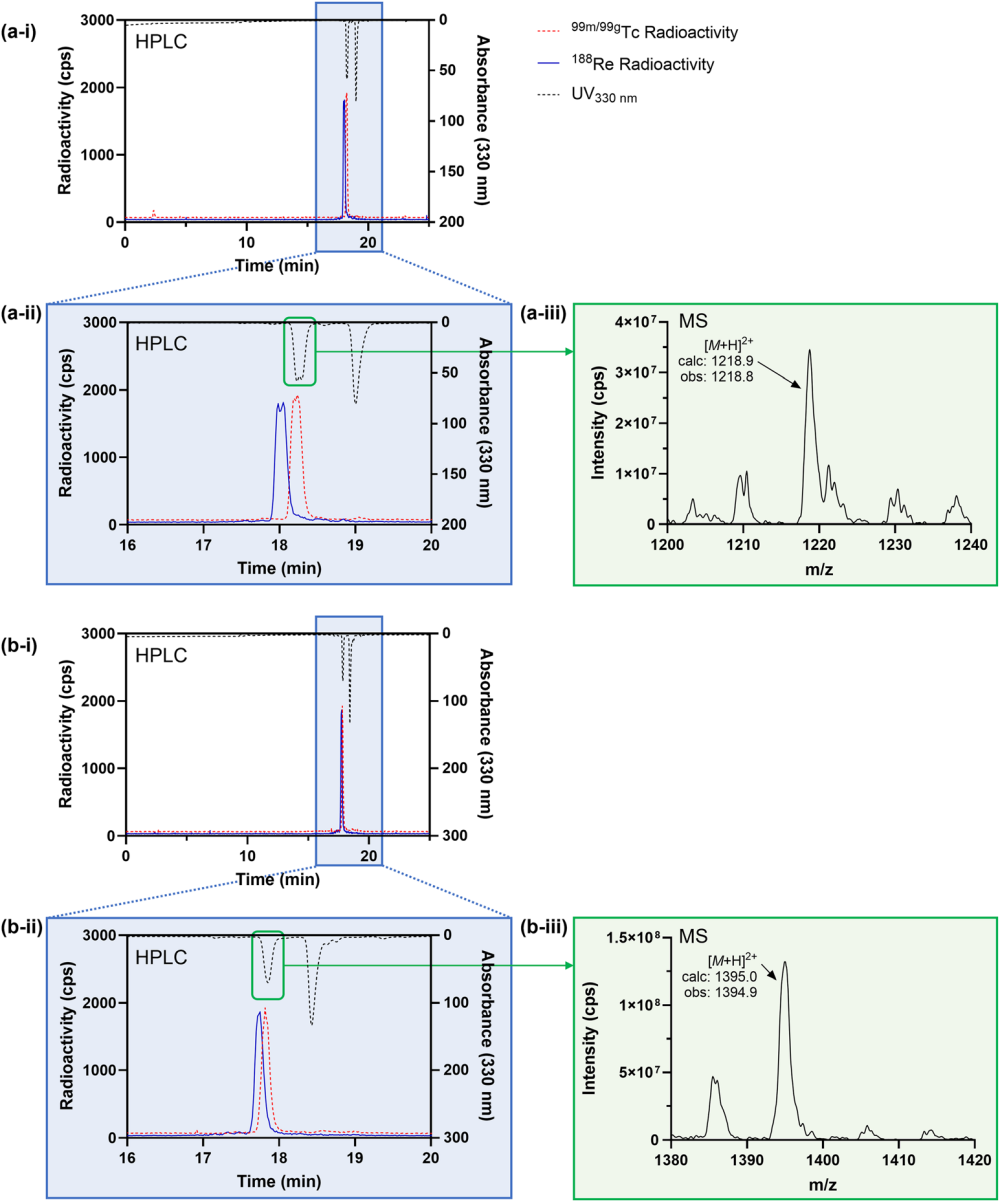
(a-i) and (a-ii) UV chromatogram and radiochromatograms of reactions containing [MO_2_(DP^An^-PSMAt)_2_]^+^ (M = ^99g^Tc, ^99m^Tc or ^188^Re); (a-iii) mass spectrum showing signal corresponding to [^99g^TcO_2_(DP^An^-PSMAt)_2_]^+^. (b-i and b-ii) UV chromatogram and radiochromatograms of reactions containing [MO_2_(DP^MEP^-PSMAt)_2_]^+^ (M = ^99g^Tc, ^99m^Tc or ^188^Re); (b-iii) mass spectrum showing signal corresponding to [^99g^TcO_2_(DP^MEP^-PSMAt)_2_]^+^.

^99g^TcO_4_^−^ (0.06 kBq) was reacted with the contents of either a DP^An^-PSMAt or DP^MEP^-PSMAt kit (Table S1), and analysed by LC-MS. Two prominent signals were observed by UV at 330 nm for each kit-based reaction containing either [^99g^TcO_2_(DP^An^-PSMAt)_2_]^+^ or [^99g^TcO_2_(DP^MEP^-PSMAt)_2_]^+^ (Fig. 5). The first signal corresponded to the desired product, as evidenced by the appearance of a signal in the associated mass spectrum corresponding to species with a formula of either [^99g^TcO_2_(DP^An^-PSMAt)_2_]^+^ ([M + H]^2+^: obs *m*/*z* = 1218.8, calc *m*/*z* = 1218.9) or [^99g^TcO_2_(DP^MEP^-PSMAt)_2_]^+^ ([M + H]^2+^: obs *m*/*z* = 1394.9, calc *m*/*z* = 1394.5). The second signal corresponded to that of unreacted DP^An^-PSMAt or DP^MEP^-PSMAt.

Each of [^99m^TcO_2_(DP^An^-PSMAt)_2_]^+^, [^99m^TcO_2_(DP^MEP^-PSMAt)_2_]^+^, [^188^ReO_2_(DP^An^-PSMAt)_2_]^+^ and [^188^ReO_2_(DP^MEP^-PSMAt)_2_]^+^ was also analysed using the same analytical reverse-phase radio-HPLC (Fig. 5). Analogous ^99g^Tc, ^99m^Tc and ^188^Re compounds exhibited highly similar HPLC retention times, with tracers based on DP^An^-PSMAt giving rise to two closely eluting peaks (for [^99m^TcO_2_(DP^An^-PSMAt)_2_]^+^*t*_R_ = 18.18 and 18.23 min; for [^188^ReO_2_(DP^An^-PSMAt)_2_]^+^*t*_R_ = 17.98 and 18.05 min). We attribute the observation of these two radioactive signals to the formation of *cis*/*trans* isomers (Fig. 1). Tc and Re complexes of DP^MEP^-PSMAt also likely result in formation of *cis*/*trans* isomers, but under these HPLC conditions, these isomers cannot be resolved ([^99m^TcO_2_(DP^MEP^-PSMAt)_2_]^+^*t*_R_ = 17.82 min; for [^188^ReO_2_(DP^MEP^-PSMAt)_2_]^+^*t*_R_ = 17.75 min). The similar HPLC behaviours of analogous ^99m^Tc and ^188^Re radiotracers is consistent with analogous pairs of ^99m^Tc/^188^Re compounds being isostructural.

### ^64^Cu radiolabelling

We and others have previously demonstrated that diphosphines can both (i) reduce ^64^Cu^2+^ to ^64^Cu^+^ in aqueous solution, and (ii) coordinate to the resulting ^64^Cu^+^ radionuclide to yield [^64^Cu(diphosphine)_2_]^+^.^19,22,23^ In these radiochemical reactions, the diphosphine is in large excess compared to the ^64^Cu radiometal.

**Fig. 6:**
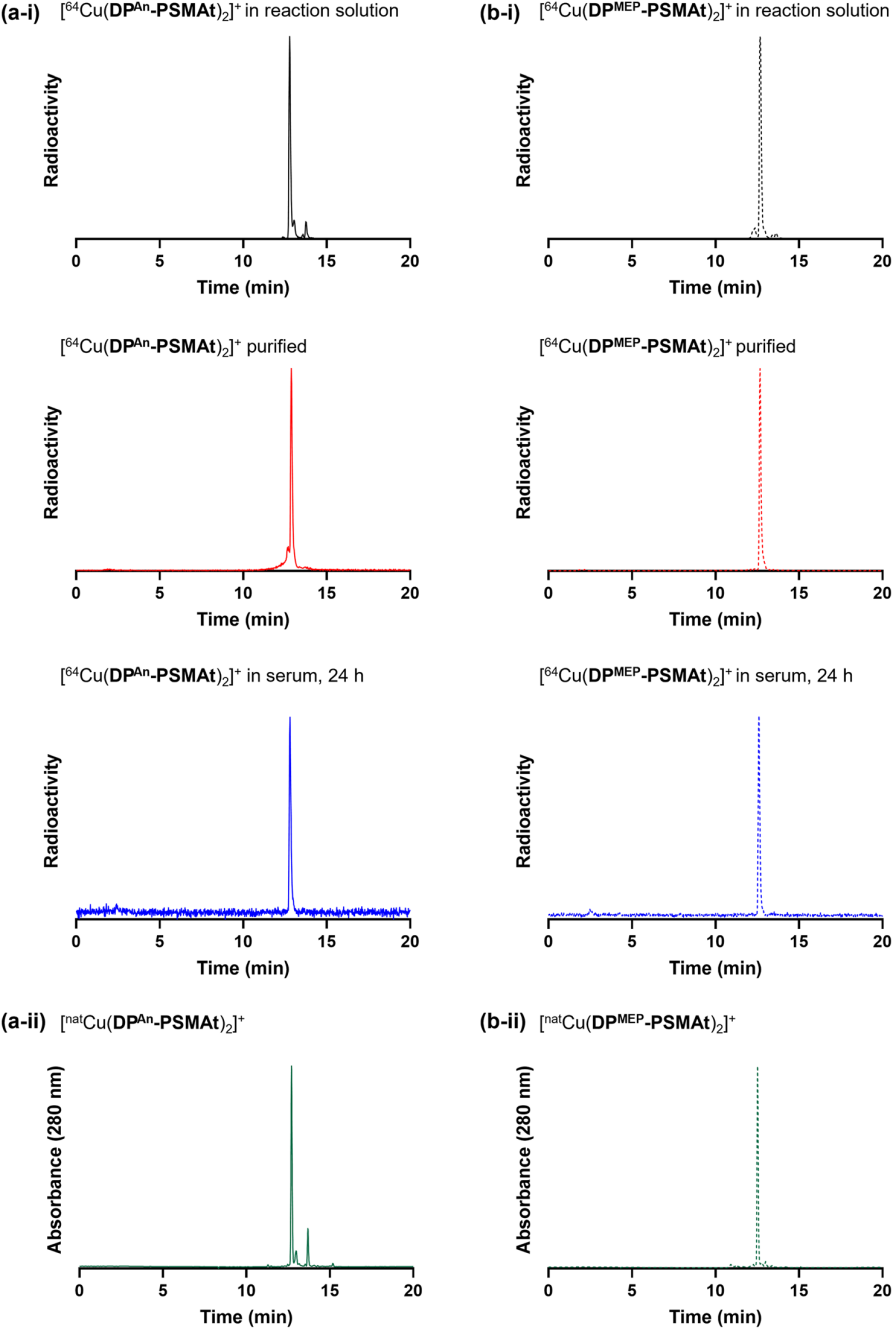
Radiochromatograms of (a-i) [^64^Cu(DP^An^-PSMAt)_2_]^+^ and (b-i) [^64^Cu(DP^MEP^-PSMAt)_2_]^+^, including radiochromatograms of ^64^Cu samples after purification and then incubation in human serum at 37 °C. Radio-HPLC analyses of serum samples indicate that ^64^Cu radiotracers possess high stability in the presence of serum proteins. UV chromatograms of (a-ii) [^nat^Cu(DP^An^-PSMAt)_2_]^+^ and (b-ii) [^nat^Cu(DP^MEP^-PSMAt)_2_]^+^.

To demonstrate the utility of these new compounds for radiolabelling with ^64^Cu for PET, each of DP^An^-PSMAt (50.0 μg, 43.3 μmol) and DP^MEP^-PSMAt (57.7 μg, 43.3 μmol) were reacted with ^64^Cu^2+^ (7–8 MBq) in an aqueous solution (0.1 M ammonium acetate, pH 7) at ambient temperature for 10 min. For each reaction, analyses by analytical reverse-phase radio-HPLC (Fig. 6) showed the formation of a single major product, which we attributed to [^64^Cu(DP^An^-PSMAt)_2_]^+^ (*t*_R_ = 12.78 min, 79% RCY), and [^64^Cu(DP^MEP^-PSMAt)_2_]^+^ (*t*_R_ = 12.67 min, 87% RCY). The radioactive signals of these products were coincident with the UV signal of the non-radioactive species, [^nat^Cu(DP^An^-PSMAt)_2_]^+^ (*t*_R_ = 12.71 min), and [^nat^Cu(DP^MEP^-PSMAt)_2_]^+^ (*t*_R_ = 12.52 min), prepared and characterised as described in the SI.^19^

The new radiotracers, [^64^Cu(DP^An^-PSMAt)_2_]^+^ and [^64^Cu(DP^MEP^-PSMAt)_2_]^+^, were purified using reverse-phase HPLC, reformulated in aqueous PBS (phosphate buffered saline) solution, and incubated with human serum at 37 °C. Radio-HPLC (Fig. 6, and SI) indicated that both [^64^Cu(DP^An^-PSMAt)_2_]^+^ and [^64^Cu(DP^MEP^-PSMAt)_2_]^+^ were present after a 24 h incubation period, with <4.1% dissociated ^64^Cu detected. Both ^64^Cu radiotracers were also stable in aqueous PBS solution (see SI).

### *In vitro* uptake of ^99m^Tc tracers in prostate cancer cells

**Fig. 7:**
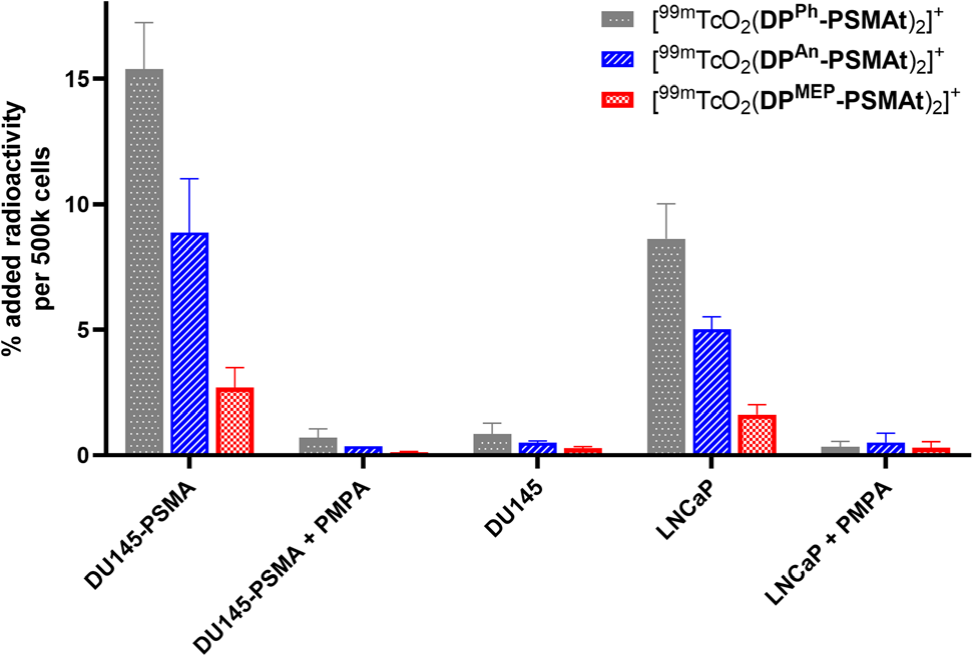
*In vitro* uptake of [^99m^TcO_2_(DP^Ph^-PSMAt)_2_]^+^, [^99m^TcO_2_(DP^An^-PSMAt)_2_]^+^ and [^99m^TcO_2_(DP^MEP^-PSMAt)_2_]^+^ radiotracers in PSMA-expressing cell lines (DU145-PSMA+ and LNCaP), in the presence of an excess of PSMA inhibitor (PMPA) in PSMA-expressing cell lines, and in a cell line that does not express PSMA (DU145).

To compare the relative uptake and specificity of the new radiotracers for PSMA, [^99m^TcO_2_(DP^Ph^-PSMAt)_2_]^+^, [^99m^TcO_2_(DP^An^-PSMAt)_2_]^+^ and [^99m^TcO_2_(DP^MEP^-PSMAt)_2_]^+^ (50 kBq) were purified from unreacted ^99m^Tc precursors and excess DP-PSMAt peptide, and each incubated with PSMA-expressing DU145-PSMA+ prostate cancer cells^33^ or LNCaP prostate cancer cells (5 × 10^5^ cells). After 1 h incubation, uptake of each radiotracer was quantified. Each radiotracer was also (i) co-incubated with the PSMA-inhibitor, PMPA (2-phosphonomethyl pentanedioic acid), in the presence of either DU145-PSMA+ cells or LNCaP cancer cells and (ii) incubated with parental DU145 cells that do not express PSMA. All three radiotracers exhibited uptake in PSMA-expressing prostate cancer cells (Fig. 7). This uptake was specific: DU145-PSMA+ and LNCaP cell uptake of each tracer could be blocked with PMPA, and there was negligible uptake in parental DU145 cells.

The PSMA-specific uptake of [^99m^TcO_2_(DP^Ph^-PSMAt)_2_]^+^ was significantly higher than that of either [^99m^TcO_2_(DP^An^-PSMAt)_2_]^+^ and [^99m^TcO_2_(DP^MEP^-PSMAt)_2_]^+^ in both DU145-PSMA+ cells and LNCaP cancer cells. For example, in DU145-PSMA+ cells, [^99m^TcO_2_(DP^Ph^-PSMAt)_2_]^+^ measured 15.4 ± 1.9 %AR (% added radioactivity), [^99m^TcO_2_(DP^An^-PSMAt)_2_]^+^ measured 8.9 ± 2.1 %AR (mean difference = 6.5 %AR, *p* = 0.02) and [^99m^TcO_2_(DP^MEP^-PSMAt)_2_]^+^ measured 2.7 ± 0.8 %AR (mean difference = 12.7 %AR, *p* = 0.003). The PSMA-specific uptake of [^99m^TcO_2_(DP^An^-PSMAt)_2_]^+^ was significantly higher than that of [^99m^TcO_2_(DP^MEP^-PSMAt)_2_]^+^ in both DU145-PSMA+ cells and LNCaP cancer cells. For example, in DU145-PSMA+ cells, mean difference = 6.2 %AR (*p* = 0.03).

### *In vivo* SPECT/CT and biodistribution studies of ^99m^Tc tracers in mice bearing prostate cancer xenografts

The biodistribution of [^99m^TcO_2_(DP^An^-PSMAt)_2_]^+^ and [^99m^TcO_2_(DP^MEP^-PSMAt)_2_]^+^ was studied using SPECT/CT imaging and *ex vivo* biodistribution methods. In these studies, [^99m^TcO_2_(DP^An^-PSMAt)_2_]^+^ and [^99m^TcO_2_(DP^MEP^-PSMAt)_2_]^+^ were used directly from kit-based formulations, without further purification, mimicking procedures used in clinical radiopharmacies and nuclear medicine departments. Mice were injected with [^99m^TcO_2_(DP^An^-PSMAt)_2_]^+^ or [^99m^TcO_2_(DP^MEP^-PSMAt)_2_]^+^ (4–5 MBq), containing 3 μg (2.6 nmol) of DP^An^-PSMAt or 3.5 μg (2.6 nmol) of (DP^MEP^-PSMAt) *via* the tail-vein, and culled 2 h post-injection, followed by dissection, organ/tissue weighing and counting.

**Fig. 8:**
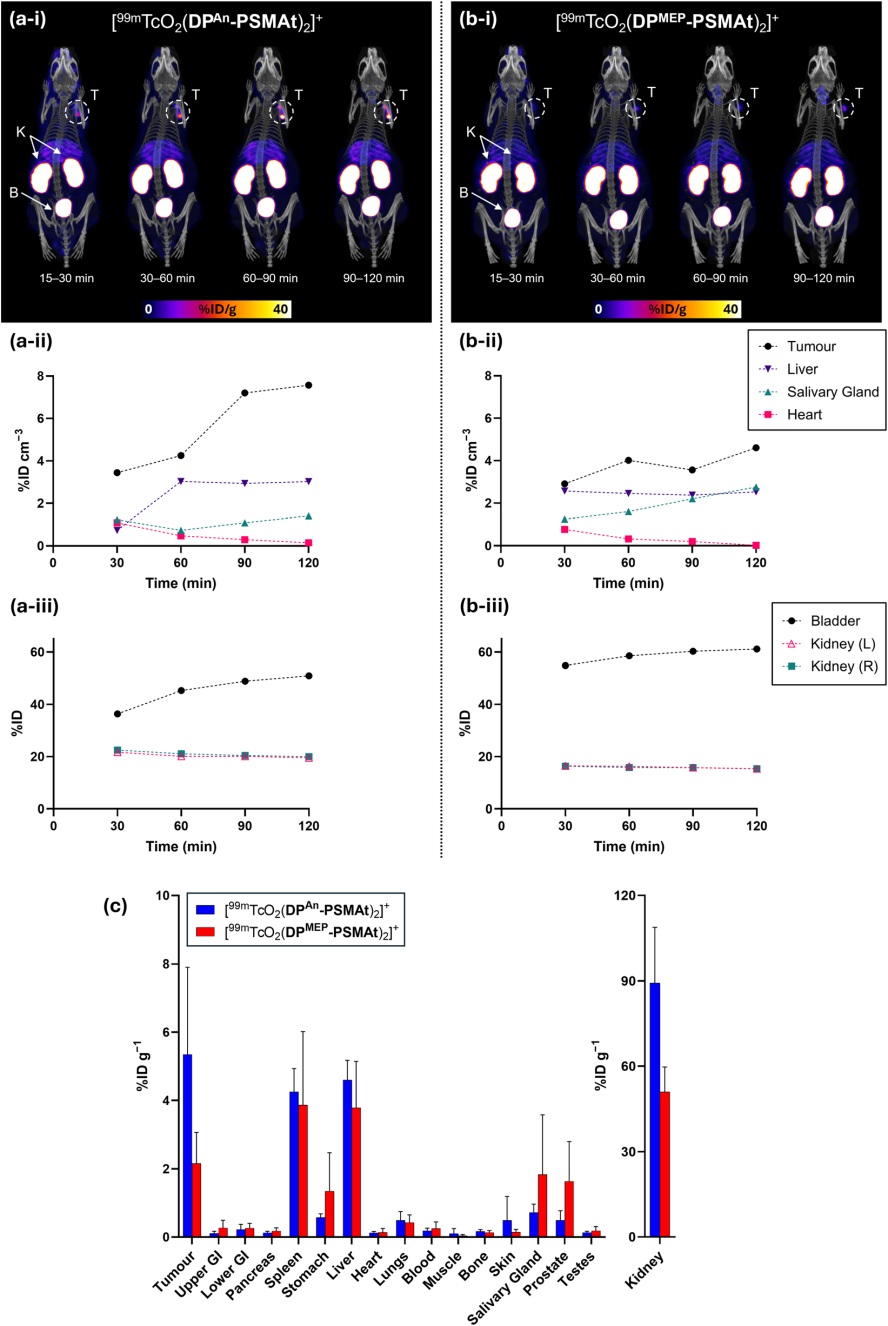
Whole body SPECT/CT maximum intensity projections and image quantification of SCID/Beige mice bearing DU145-PSMA+ tumours administered either (a) [^99m^TcO_2_(DP^An^-PSMAt)_2_]^+^ or (b) [^99m^TcO_2_(DP^MEP^-PSMAt)_2_]^+^. T = tumour, K = kidneys, B = bladder. (c) Biodistribution (2 h post-injection) of [^99m^TcO_2_(DP^An^-PSMAt)_2_]^+^ or [^99m^TcO_2_(DP^MEP^-PSMAt)_2_]^+^ in SCID/Beige mice bearing DU145-PSMA+ prostate cancer xenografts (mean ± SD, *n* = 3–4).

SPECT/CT studies were undertaken in male SCID/Beige mice bearing DU145-PSMA+ prostate cancer xenografts administered [^99m^TcO_2_(DP^An^-PSMAt)_2_]^+^ and [^99m^TcO_2_(DP^MEP^-PSMAt)_2_]^+^. SPECT images were acquired from 15 to 120 min post-injection of radiotracer. In SPECT/CT scans of mice administered either [^99m^TcO_2_(DP^An^-PSMAt)_2_]^+^ or [^99m^TcO_2_(DP^MEP^-PSMAt)_2_]^+^, tumours (T) could be delineated, with higher tumour to background ratios at later time points (Fig. 8). The kidneys (K) and bladder (B) were also clearly visible across all these timepoints with low background levels, indicating that both the radiotracers are rapidly cleared from the blood pool *via* a renal pathway.

Quantification of SPECT/CT images (acquired at 15–30 min, 30–60 min, 60–90 min and 90–120 min post-injection, Fig. 8) indicated that tumour uptake of [^99m^TcO_2_(DP^An^-PSMAt)_2_]^+^ was higher than that of [^99m^TcO_2_(DP^MEP^-PSMAt)_2_]^+^, consistent with *in vitro* uptake studies and *ex vivo* biodistribution data (*vide infra*). Additionally, for the mouse administered [^99m^TcO_2_(DP^An^-PSMAt)_2_]^+^, the ^99m^Tc tumour concentration noticeably increased from 30 min until 2 h post-injection.

SPECT/CT indicated that ^99m^Tc residualised in salivary glands for animals administered either [^99m^TcO_2_(DP^An^-PSMAt)_2_]^+^ or [^99m^TcO_2_(DP^MEP^-PSMAt)_2_]^+^. Two well-documented mechanisms of radiotracer uptake likely account for salivary gland uptake:

(i) PSMA is expressed at low levels in the salivary glands, kidneys and small intestine, with PSMA-targeted radiotracers showing uptake in the salivary glands, in both mice and human patients.^34–36^ Indeed, the salivary glands are considered dose-limiting organs, and uptake of radiotherapeutic PSMA-targeted radiopharmaceuticals can lead to xerostomia in prostate cancer patients.^36^

(ii) TcO_4_^−^, bearing a single negative charge and with a similar polyatomic radius to that of the iodide anion, acts as a substrate for the sodium iodide symporter *in vivo*,^37^ and therefore accumulates in organs expressing the sodium iodide symporter – in mice, this includes the thyroid, salivary glands and stomach.^38^ SPECT imaging quantification (consistent with biodistribution data, *vide infra*) indicated that ^99m^Tc concentrations were higher in the salivary glands for the mouse administered [^99m^TcO_2_(DP^MEP^-PSMAt)_2_]^+^ compared to the mouse administered [^99m^TcO_2_(DP^An^-PSMAt)_2_]^+^. Indeed, ^99m^Tc concentrations in the salivary glands increased over time for the former subject. This suggests that [^99m^TcO_2_(DP^MEP^-PSMAt)_2_]^+^ could possess lower *in vivo* stability than [^99m^TcO_2_(DP^An^-PSMAt)_2_]^+^, with complex dissociation and oxidation of the [^99m^Tc^V^O_2_]^+^ motif resulting in formation of ^99m^TcO_4_^−^*in vivo*, and increased ^99m^Tc accumulation in the salivary glands for animals administered [^99m^TcO_2_(DP^MEP^-PSMAt)_2_]^+^.

The biodistribution of [^99m^TcO_2_(DP^An^-PSMAt)_2_]^+^ and [^99m^TcO_2_(DP^MEP^-PSMAt)_2_]^+^ was further studied in (i) male SCID/Beige mice bearing DU145-PSMA+ prostate cancer xenografts (Fig. 8) and (ii) male nude mice bearing PSMA-expressing LNCaP prostate cancer xenografts (see SI).

For all animals, significant concentrations of ^99m^Tc radioactivity (2–5 %ID g^−1^) were measured in PSMA-expressing tumours 2 h post-injection, and although ^99m^Tc tumour concentrations were consistently higher for animals administered [^99m^TcO_2_(DP^An^-PSMAt)_2_]^+^, there were no statistically significant differences between [^99m^TcO_2_(DP^An^-PSMAt)_2_]^+^ and [^99m^TcO_2_(DP^MEP^-PSMAt)_2_]^+^ in either mouse model. For both tracers, there were also significant concentrations of ^99m^Tc radioactivity in the spleen, which is known to express low levels of PSMA and accumulate PSMA-targeted radiotracers,^9,10^ and the liver. Importantly, clearance from the blood pool and subsequent excretion was largely *via* a renal route, with concentrations of 40–90 %ID g^−1^ measured in kidneys.

We also noted that for both SCID/beige and nude mice studies, higher ^99m^Tc concentrations were observed in the stomach and salivary glands for animals administered [^99m^TcO_2_(DP^MEP^-PSMAt)_2_]^+^ compared to animals administered [^99m^TcO_2_(DP^An^-PSMAt)_2_]^+^, although these differences were not statistically significant. This observation is consistent with observations from SPECT/CT imaging studies, and further supports our conjecture that [^99m^TcO_2_(DP^MEP^-PSMAt)_2_]^+^ has lower *in vivo* stability than [^99m^TcO_2_(DP^An^-PSMAt)_2_]^+^, with *in vivo* formation of ^99m^TcO_4_^−^ from dissociation of [^99m^TcO_2_(DP^MEP^-PSMAt)_2_]^+^ resulting in higher levels of ^99m^Tc activity in organs expressing the sodium iodide symporter.

Overall, the *in vivo* biodistribution demonstrates that both [^99m^TcO_2_(DP^An^-PSMAt)_2_]^+^ and [^99m^TcO_2_(DP^MEP^-PSMAt)_2_]^+^ are useful SPECT or γ-scintigraphy imaging agents for PSMA expression in prostate cancer.

### Stability of [MO_2_(DP^An^-PSMAt)_2_]^+^ and [MO_2_(DP^MEP^-PSMAt)_2_]^+^ (M = ^99m^Tc, ^188^Re)

Both [^99m^TcO_2_(DP^An^-PSMAt)_2_]^+^ and [^99m^TcO_2_(DP^MEP^-PSMAt)_2_]^+^, prepared using kits, were incubated at 37 °C in human serum for up to 24 h. Analytical reverse-phase radio-HPLC (Fig. 3) demonstrated that >98% of both radiotracers remained intact in serum. [^188^ReO_2_(DP^An^-PSMAt)_2_]^+^ and [^188^ReO_2_(DP^MEP^-PSMAt)_2_]^+^, prepared using kits and then isolated using reverse-phase HPLC in >98% radiochemical purity, were also incubated in human serum under the same conditions. Radio-HPLC (Fig. 4) similarly showed that ^188^Re-labelled radiotracers were stable in serum over 24 h, with <1.5% of ^188^Re dissociating over this time. These ^99m^Tc and ^188^Re radiotracers were also stable when left to stand in kit-based reaction solutions, or in PBS solution (see SI for full chromatograms).

Urine was also collected in *in vivo*^99m^Tc biodistribution experiments (from male SCID/Beige mice bearing DU145-PSMA+ prostate cancer xenografts at 2 h post-injection) and analysed using reverse-phase radio-HPLC (Fig. 3). Radio-chromatograms indicated that both [^99m^TcO_2_(DP^An^-PSMAt)_2_]^+^ and [^99m^TcO_2_(DP^MEP^-PSMAt)_2_]^+^ were excreted in urine intact, indicating that both radiotracers have high stability *in vivo*.

## Discussion

We have recently reported DP^Ph^ and DP^Tol^ derivatives of the 2,3-bis(diphenylphosphino)maleic anhydride platform, and shown that the resulting ^99m^Tc and ^188^Re radiotracers have desirable properties as molecular theranostic agents.^19,20^ Here, we have optimised this platform technology through development of DP^An^ and DP^MEP^, and demonstrated that these compounds can be attached to a variety of amine-containing bioactive molecules, including peptides, carbohydrates, vitamins and small molecule inhibitors. The resulting diphosphine conjugates can be radiolabelled with ^99m^Tc, ^188^Re and ^64^Cu, thus enabling formation of radiotracers for molecular SPECT imaging, radiotherapy and molecular PET imaging, respectively.

Significantly, as hypothesised, the increased σ-donating ability of DP^An^ and DP^MEP^ derivatives, due to *para* substituents on aryl rings of the phosphines, leads to further increased RCYs within this family of diphosphine chelators (DP^An^ ∼ DP^MEP^ > DP^Tol^ > DP^Ph^). The kit formulations of DP^An^ and DP^MEP^ conjugates developed here use relatively small amounts of DP^An^-PSMAt and DP^MEP^-PSMAt diphosphine bioconjugates (110 nmol, 113–145 μg), minimising competitive target receptor binding of unreacted ligand present in formulations administered to *in vivo* subjects. The high RCYs (≥95%) of [^99m^TcO_2_(DP^An^-PSMAt)_2_]^+^ and [^99m^TcO_2_(DP^MEP^-PSMAt)_2_]^+^ achieved using our one-step kit formulation negate the need for time-intensive and often complicated purification procedures to remove unreacted ^99m^Tc precursors. In a clinical context, rapid, one-step radiolabelling and formulation protocols using kits are desirable: this simplicity of producing a radiopharmaceutical for immediate patient injection makes widespread clinical adoption feasible. We are yet to optimise ^188^Re radiolabelling protocols including kit formulations for new ^188^Re radiotracers, however, we note that the radiochemical yields (>83%) are significantly improved compared to RCYs of prior DP^Ph^ and DP^Tol^ conjugate derivatives.^20^ In these previous reactions, ^188^Re radiotracer yields were often highly variable, and commonly less than 30%.

The log *D*_OCT/PBS_ values observed here are all lower than those previously reported for [^99m^TcO_2_(DP^Ph/Tol^-PSMAt)_2_]^+^ derivatives, consistent with the more hydrophilic methoxy and methyl ethylene glycol substituents of aryl rings, compared to DP^Ph^ and DP^Tol^ analogues. This also poses potential advantages for receptor-targeted biomolecular vectors that are significantly more hydrophobic than the PSMAt pharmacophore tested here.

We observe decreasing uptake of [^99m^TcO_2_(DP^X^-PSMAt)_2_]^+^ (X = Ph, An, MEP) radiotracer analogues in PSMA-positive prostate cancer cells in the order Ph > An > MEP and we note that this corresponds with increasing size and hydrophilicity of the *para* substituents: H < OCH_3_ < OCH_2_CH_2_OCH_3_. It is well documented that aromatic groups are important in PSMA affinity, due to the hydrophobic binding pocket of PSMA.^39,40^ We postulate that the *para* substituents interfere with binding of the radiotracer to PSMA.

In the biodistribution experiments undertaken for this work, prostate cancer tumour uptake of [^99m^TcO_2_(DP^An^-PSMAt)_2_]^+^ and [^99m^TcO_2_(DP^MEP^-PSMAt)_2_]^+^ was lower than previously reported for [^99m^TcO_2_(DP^Ph^-PSMAt)_2_]^+^ and [^99m^TcO_2_(DP^Tol^-PSMAt)_2_]^+^ in the same murine tumour models.^20^ This was expected: here, kit-based formulations of [^99m^TcO_2_(DP^An^-PSMAt)_2_]^+^ and [^99m^TcO_2_(DP^MEP^-PSMAt)_2_]^+^ were not purified from unreacted, excess DP^An^-PSMAt and DP^MEP^-PSMAt respectively, prior to injection. It is likely that DP^An^-PSMAt and DP^MEP^-PSMAt compete for binding to PSMA receptor *in vivo*.

Liver concentrations of [^99m^TcO_2_(DP^An^-PSMAt)_2_]^+^ and [^99m^TcO_2_(DP^MEP^-PSMAt)_2_]^+^ were observed to be higher than that previously measured for first-generation [^99m^TcO_2_(DP^Ph^-PSMAt)_2_]^+^ and [^99m^TcO_2_(DP^Tol^-PSMAt)_2_]^+^ radiotracers in the same murine models. An increased liver accumulation of a tracer can correlate with increased hydrophobicity of compounds, but we note that log *D*_OCT/PBS_ values indicate that [^99m^TcO_2_(DP^An^-PSMAt)_2_]^+^ and [^99m^TcO_2_(DP^MEP^-PSMAt)_2_]^+^ are both more hydrophilic than their predecessors. We therefore attribute the higher liver accumulation of [^99m^TcO_2_(DP^An^-PSMAt)_2_]^+^ and [^99m^TcO_2_(DP^MEP^-PSMAt)_2_]^+^ to either their increased size, or alternatively, the different formulation of the radiotracer dose, which includes unlabelled bioconjugate precursor, which could affect the biodistribution.

Compared to animals administered [^99m^TcO_2_(DP^An^-PSMAt)_2_]^+^, animals administered [^99m^TcO_2_(DP^MEP^-PSMAt)_2_]^+^ presented higher ^99m^Tc concentrations in salivary glands and the stomach. This could indicate that [^99m^TcO_2_(DP^MEP^-PSMAt)_2_]^+^ exhibits a degree of instability *in vivo*, as both the salivary glands and the stomach express the sodium iodide symporter and are known to take up ^99m^TcO_4_^−^.^38^ It is also possible that some salivary gland uptake is due to known salivary gland PSMA expression.^34,35^ However, except for the possible formation of small amounts of ^99m^TcO_4_^−^*in vivo*, both [^99m^TcO_2_(DP^An^-PSMAt)_2_]^+^ and [^99m^TcO_2_(DP^MEP^-PSMAt)_2_]^+^ demonstrated high metabolic stability, as evidenced by their excretion intact in urine, and their high stability in serum (*ex vivo*) over 24 h.

Our new diphosphine chelator technology has advantages over other ^99m^Tc and ^188^Re radiolabelling platforms. For instance, the radiosynthesis and purification of ^99m^Tc-MIP-1404, which is based on the [^99m^Tc(CO)_3_]^+^ synthon, is time-consuming. It requires (i) formation of an intermediate *fac*-[^99m^Tc(CO)_3_(H_2_O)_3_]^+^ precursor prior to (ii) chelation with the tridentate MIP-1404 chelator-peptide, and finally (iii) purification and formulation before administration.^9^ Other molecular ^99m^Tc-radiopharmaceuticals, including ^99m^Tc-EDDA/HYNIC-octreotide^1^ and ^99m^Tc-EDDA/HYNIC-iPSMA,^8,9^ utilise the 6-hydrazinopyridine-3-carboxylic acid (HYNIC) platform, which coordinates to ^99m^Tc and acts as the attachment point for the bioactive receptor-targeting vectors. The ethylenediamine co-ligand chelates to the remaining coordination sites on the Tc metal centre. Whilst some of these HYNIC-based radiopharmaceuticals can be prepared from a single kit,^13^ their structures remain ill-defined:^41,42^ it is unknown whether HYNIC coordinates to Tc *via* the hydrazine group only, or as a bidentate ligand, *via* the hydrazine and pyridyl groups. Importantly, HYNIC-based bioconjugates are not known to provide isostructural Tc and Re derivatives,^41,42^ and therefore cannot be used to develop dual ^99m^Tc/^188^Re theranostic tracers.

Compared with the existing ^99m^Tc-radiolabeled PSMA-targeted tracers, ^99m^Tc-MIP-1404, ^99m^Tc-PSMA-I&S, and ^99m^Tc-EDDA/HYNIC-iPSMA, the new [^99m^TcO_2_(DP^An^-PSMAt)_2_]^+^ and [^99m^TcO_2_(DP^MEP^-PSMAt)_2_]^+^ radiotracers demonstrate either increased or comparable blood clearance at 1–2 h after administration (Table 5). [^99m^TcO_2_(DP^An^-PSMAt)_2_]^+^ exhibits either decreased or comparable residualisation in murine liver compared to these other PSMA-targeted tracers.

**Table 5:** Comparison of biodistribution of ^99m^Tc-labeled PSMA-targeted radiotracers

Radiotracer (time post-injection)	Blood^a^	Liver^a^	Kidney^a^
[^99m^TcO_2_(DP^An^-PSMAt)_2_]^+^ (athymic nude, 2 h)	0.11 ± 0.04	0.90 ± 0.15	87.6 ± 13.3
[^99m^TcO_2_(DP^MEP^-PSMAt)_2_]^+^ (athymic nude, 2 h)	0.17 ± 0.10	7.38 ± 3.98	46.4 ± 23.2
[^99m^TcO_2_(DP^Ph^-PSMAt)_2_]^+^ (athymic nude, 2 h)^20^	0.23 ± 0.05	0.35 ± 0.05	203.56 ± 13.67
[^99m^TcO_2_(DP^Tol^-PSMAt)_2_]^+^ (athymic nude, 2 h)^20^	0.28 ± 0.05	0.52 ± 0.05	212.23 ± 29.49
^99m^Tc-MIP-1404 (athymic nude, 1 h)^9^	0.13 ± 0.03	0.14 ± 0.03	105 ± 37
^99m^Tc-PSMA-I&S (SCID, 1 h)^10^	1.73 ± 0.50	1.58 ± 0.24	186 ± 23
^99m^Tc-EDDA/HYNIC-iPSMA (athymic, 1 h)^13^	0.18 ± 0.08	2.18 ± 0.19	23.63 ± 3.56

a % ID g^−1^, ± standard deviation.

These diphosphine chelators also enable simple, one-pot preparation of stable molecular radiotracers based on radioactive copper isotopes, such as ^64^Cu for PET imaging, and its “theranostic” companion, ^67^Cu (*t*_½_ = 61.9 h; 100% β^−^, *E*_max_ = 561 keV; 44% γ, 185 keV), which emits β^−^ particles and has utility for systemic radiotherapy.^43^ Critically, this allows application of the same diphosphine-peptide bioconjugate for theranostic radiopharmaceuticals that span both SPECT and PET imaging.

## Concluding remarks

In summary, our new and highly versatile bis(phosphino) maleic anhydride platforms, DP^An^ and DP^MEP^, enable facile preparation of diphosphine bioconjugates that can be simply radiolabelled with ^99m^Tc for SPECT imaging, ^64^Cu for PET imaging and ^188^Re for systemic radiotherapy, leading to the possibility of theranostic radiotracers. Importantly, these ^99m^Tc radiotracers can be prepared in high radiochemical yields (≥95%) using a single step kit. This is a critical advance upon our prior DP^Ph^ and DP^Tol^ technology, as it allows for simple, one-step formulation of peptide-based ^99m^Tc radiotracers, and presents new opportunities for economical molecular imaging using widely available ^99m^Tc production infrastructure and γ-scintigraphy/SPECT cameras. We are currently expanding the application of DP^An^ and DP^MEP^ chemistry to other therapeutically relevant receptor targets, to develop a versatile suite of molecular SPECT, PET and radiotherapeutic tracers.

## Author contributions

REN conceived research, designed experiments, undertook experimental work and drafted the manuscript; MTM and PGP conceived research, designed experiments and drafted the manuscript; INH, OWLC, AR, NP, ZY, JC and JDY undertook experimental work; TTP conceived research and undertook experimental work; JS, GJRC and LL contributed to experimental design; NV and HHT provided a ^188^W/^188^Re Oncobeta generator and provided expertise on its use and potential clinical applications.

## Conflicts of interest

Some of the authors have submitted a patent application describing the intellectual property described herein. Nicholas Vetter is CEO of OncoBeta, who provide ^188^Re generators, including those used in this study. No other potential conflicts of interest relevant to this article exist.

## Supplementary Material

SC-OLF-D5SC02110C-s001

## Data Availability

The data supporting this article, including abbreviations, experimental procedures, characterisation data, additional figures, chromatograms and spectra have been included as part of the SI. See DOI: https://doi.org/10.1039/d5sc02110c.

## References

[cit1] Gabriel M., Decristoforo C., Donnemiller E., Ulmer H., Rychlinski C. W., Mather S. J., Moncayo R. (2003). J. Nucl. Med..

[cit2] Hofman M. S., Kong G., Neels O. C., Eu P., Hong E., Hicks R. J. (2012). J. Med. Imaging Radiat. Oncol..

[cit3] Shinto A. (2017). Curr. Trends Clin. Med. Imaging..

[cit4] Jackson J. A., Hungnes I. N., Ma M. T., Rivas C. (2020). Bioconjugate Chem..

[cit5] Rivas C., Jackson J. A., Hungnes I. N., Ma M. T. (2021). Compr. Coord. Chem. III.

[cit6] Riondato M., Rigamonti D., Martini P., Cittanti C., Boschi A., Urso L., Uccelli L. (2023). J. Med. Chem..

[cit7] Liu S., Chakraborty S. (2011). Dalton Trans..

[cit8] Schmidkonz C., Hollweg C., Beck M., Reinfelder J., Goetz T. I., Sanders J. C., Schmidt D., Prante O., Bäuerle T., Cavallaro A., Uder M., Wullich B., Goebell P., Kuwert T., Ritt P. (2018). Prostate.

[cit9] Hillier S. M., Maresca K. P., Lu G., Merkin R. D., Marquis J. C., Zimmerman C. N., Eckelman W. C., Joyal J. L., Babich J. W. (2013). J. Nucl. Med..

[cit10] Robu S., Schottelius M., Eiber M., Maurer T., Gschwend J., Schwaiger M., Wester H. J. (2017). J. Nucl. Med..

[cit11] Maurer T., Robu S., Schottelius M., Schwamborn K., Rauscher I., van den Berg N. S., van Leeuwen F. W. B., Haller B., Horn T., Heck M. M., Gschwend J. E., Schwaiger M., Wester H. J., Eiber M. (2019). Eur. Urol..

[cit12] Lawal I. O., Ankrah A. O., Mokgoro N. P., Vorster M., Maes A., Sathekge M. M. (2017). Prostate.

[cit13] Ferro-Flores G., Luna-Gutiérrez M., Ocampo-García B., Santos-Cuevas C., Azorín-Vega E., Jiménez-Mancilla N., Orocio-Rodríguez E., Davanzo J., García-Pérez F. O. (2017). Nucl. Med. Biol..

[cit14] Bernal P., Raoul J. L., Stare J., Sereegotov E., Sundram F. X., Kumar A., Jeong J. M., Pusuwan P., Divgi C., Zanzonico P., Vidmar G., Buscombe J., Chau T. T. M., Saw M. M., Chen S., Ogbac R., Dondi M., Padhy A. K. (2008). Semin. Nucl. Med..

[cit15] Shinto A. (2017). World J. Nucl. Med..

[cit16] Shinto A., Mallia M., Kameswaran M., Kamaleshwaran K., Joseph J., Radhakrishnan E., Upadhyay I., Subramaniam R., Sairam M., Banerjee S., Dash A. (2018). World J. Nucl. Med..

[cit17] Cardinale J., Giesel F. L., Wensky C., Rathke H. G., Haberkorn U., Kratochwil C. (2023). J. Nucl. Med..

[cit18] Hungnes I. N., Al-Salemee F., Gawne P. J., Eykyn T., Atkinson R. A., Terry S. Y. A., Clarke F., Blower P. J., Pringle P. G., Ma M. T. (2021). Dalton Trans..

[cit19] Hungnes I. N., Pham T. T., Rivas C., Jarvis J. A., Nuttall R. E., Cooper S. M., Young J. D., Blower P. J., Pringle P. G., Ma M. T. (2023). Inorg. Chem..

[cit20] Pham T. T., Hungnes I. N., Rivas C., Cleaver J., Firth G., Blower P. J., Sosabowski J., Cook G. J. R., Livieratos L., Young J. D., Pringle P. G., Ma M. T. (2024). J. Nucl. Med..

[cit21] Nuttall R. E., Pham T. T., Chadwick A. C., Hungnes I. N., Firth G., Heckenast M. A., Sparkes H. A., Galan M. C., Ma M. T., Pringle P. G. (2023). Inorg. Chem..

[cit22] Lewis J. S., Zweit J., Dearling J. L. J., Rooney B. C., Blower P. J. (1996). Chem. Commun..

[cit23] Lewis J. S., Heath S. L., Powell A. K., Zweit J., Blower P. J. (1997). J. Chem. Soc., Dalton Trans..

[cit24] Dundas C. M., Demonte D., Park S. (2013). Appl. Microbiol. Biotechnol..

[cit25] Bongarzone S., Sementa T., Dunn J., Bordoloi J., Sunassee K., Blower P. J., Gee A. (2020). J. Med. Chem..

[cit26] Müller C., Schibli R. (2011). J. Nucl. Med..

[cit27] Fernández M., Javaid F., Chudasama V. (2018). Chem. Sci..

[cit28] Imberti C., Terry S. Y. A., Cullinane C., Clarke F., Cornish G. H., Ramakrishnan N. K., Roselt P., Cope A. P., Hicks R. J., Blower P. J., Ma M. T. (2017). Bioconjugate Chem..

[cit29] Kratochwil C., Flechsig P., Lindner T., Abderrahim L., Altmann A., Mier W., Adeberg S., Rathke H., Röhrich M., Winter H., Plinkert P. K., Marme F., Lang M., Kauczor H. U., Jäger D., Debus J., Haberkorn U., Giesel F. L. (2019). J. Nucl. Med..

[cit30] Anton D. R., Crabtree R. H. (1983). Organometallics.

[cit31] Gali H., Hoffman T. J., Sieckman G. L., Owen N. K., Katti K. V., Volkert W. A. (2001). Bioconjugate Chem..

[cit32] Kothari K. K., Gali H., Prabhu K. R., Pillarsetty N., Owen N. K., Katti K. V., Hoffman T. J., Volkert W. A. (2002). Nucl. Med. Biol..

[cit33] Kampmeier F., Williams J. D., Maher J., Mullen G. E., Blower P. J. (2014). EJNMMI Res..

[cit34] Roy J., Warner B. M., Basuli F., Zhang X., Wong K., Pranzatelli T., Ton A. T., Chiorini J. A., Choyke P. L., Lin F. I., Jagoda E. M. (2020). Cancer Biother. Radiopharm..

[cit35] Piron S., Verhoeven J., De Coster E., Descamps B., Kersemans K., Pieters L., Vral A., Vanhove C., De Vos F. (2021). Sci. Rep..

[cit36] Heynickx N., Herrmann K., Vermeulen K., Baatout S., Aerts A. (2021). Nucl. Med. Biol..

[cit37] Chung J.-K. (2002). J. Nucl. Med..

[cit38] Man F., Lim L., Volpe A., Gabizon A., Shmeeda H., Draper B., Parente-Pereira A. C., Maher J., Blower P. J., Fruhwirth G. O., de Rosales R. T. M. (2019). Mol. Ther..

[cit39] Benešová M., Bauder-Wüst U., Schäfer M., Klika K. D., Mier W., Haberkorn U., Kopka K., Eder M. (2016). J. Med. Chem..

[cit40] Benesová M., Schäfer M., Bauder-Wüst U., Afshar-Oromieh A., Kratochwil C., Mier W., Haberkorn U., Kopka K., Eder M. (2015). J. Nucl. Med..

[cit41] King R. C., Surfraz M. B. U., Biagini S. C. G., Blower P. J., Mather S. J. (2007). Dalton Trans..

[cit42] Meszaros L. K., Dose A., Biagini S. C. G., Blower P. J. (2010). Inorg. Chim. Acta.

[cit43] Rudd S. E., Van Zuylekom J., Cullinane C., Blyth B. J., Donnelly P. S. (2025). Chem. Sci..

