# Supplementary material for: Maleic anhydride derived diphosphines: adaptable chelators for receptor-targeted 99mTc, 64Cu and 188Re radiotracers
Source: Chem Sci. 2025 Aug 26;16(37):17112–26. doi: 10.1039/d5sc02110c (PMC12435366; doi:10.1039/d5sc02110c)
Supplement: SC-016-D5SC02110C-s001 [file SC-016-D5SC02110C-s001.pdf]

Supplementary Information (SI) for Chemical Science.
This journal is © The Royal Society of Chemistry 2025

Supporting Information

Maleic anhydride derived diphosphines: adaptable chelators for receptor-targeted ^{99m}Tc, ^{64}Cu and ^{188}Re radiotracers

Rachel E. Nuttall,[1*] Ingebjørg N. Hungnes,[1] Truc T. Pham,[1] Oliver W. L. Carter,[1] Alex Rigby,[1] Natasha Patel,[1] Zilin Yu,[1] Julie Cleaver,[2] Jennifer D. Young,[1,2] Gary J. R. Cook,[1] Lefteris Livieratos,[1,3] Jane Sosabowski,[2] Hong Hoi Ting,[4] Nicholas Vetter,[4] Paul G. Pringle,[5] and Michelle T. Ma[1*]

[1]School of Bioengineering and Imaging Sciences, King's College London, 4th Floor Lambeth Wing, St Thomas' Hospital, London, SE1 7EH, UK

[2]Centre for Cancer Biomarkers and Biotherapeutics, Barts Cancer Institute, Queen Mary University of London, John Vane Science Centre, Charterhouse Square, London, EC1M 6BQ, UK

[3]Department of Nuclear Medicine, Guy's and St Thomas' Hospitals NHS Foundation Trust, Guy's Hospital, London, SE1 9RT, UK

[4]Oncobeta GmbH, 85748 Garching, Munich, Germany

[5]School of Chemistry, University of Bristol, Cantock's Close, Bristol, BS8 1TS, UK

*Corresponding authors: rachel.nuttall@kcl.ac.uk, (ORCID: 0000-0002-3945-3096), michelle.ma@kcl.ac.uk (ORCID: 0000-0002-3349-7346)

Table of Contents

1 Abbreviations 4

2 General experimental 5

3 Experimental procedures 8

3.1 Bis(*para*-methoxyphenyl)phosphine 8

3.2 2,3-Bis(bis(*p*-MeOC_6H_4)phosphino)maleic anhydride, **DP^{An}** 8

3.3 4-Bromo-(2-methoxyethoxy)benzene 8

3.4 (Diethylamino)bis(*para*-(2-methoxyethoxy)phenyl)phosphine 9

3.5 Bis(*para*-(2-methoxyethoxy)phenyl)chlorophosphine 9

3.6 Bis(*para*-(2-methoxyethoxy)phenyl)phosphine 10

3.7 2,3-Bis(bis(*p*-MeO$CH_2CH_2OC_6H_4$)phosphino)maleic anhydride, **DP^{MEP}** 10

3.8 DP^{An}-PSMAt 11

3.9 DP^{MEP}-PSMAt 11

3.10 Conjugation screen general procedure 12

3.11 **DP^{An}** thiol reaction general procedure 12

3.12 Mo(0) coordination general procedure 12

3.12.1 *cis*-[Mo(CO)$_4$(**DP^{Ph}**)] 12

3.12.2 *cis*-[Mo(CO)$_4$(**DP^{An}**)] 13

3.12.3 *cis*-[Mo(CO)$_4$(**DP^{MEP}**)] 13

3.13 Ring-opening of Mo(0) anhydride complexes general procedure 13

3.13.1 [MOE-NH_3][Mo(CO)$_4$(**DP^{Ph}-NH-MOE**)] 14

3.13.2 [MOE-NH_3][Mo(CO)$_4$(**DP^{An}-NH-MOE**)] 14

3.13.3 [MOE-NH_3][Mo(CO)$_4$(**DP^{MEP}-NH-MOE**)] 15

3.14 [Cu(**DP^{An}-PSMAt**)]$^+$ 15

3.15 [Cu(**DP^{MEP}-PSMAt**)]$^+$ 16

4 Radiolabelling procedures and radiotracer characterisation 17

4.1 Kit preparation 17

4.2 Radiolabelling with ^{99m}Tc 17

4.3 Log$_{7.4}$*D* – ^{99m}Tc 18

4.4 Radiolabelling with ^{188}Re 18

4.5 Log$_{7.4}$*D* – ^{188}Re 19

4.6 Preparation of ^{99g}Tc complexes using $^{99g}TcO_4^-$ 19

4.7 Radiolabelling with ^{64}Cu 19

4.8 Stability studies 20

4.8.1 Kit stability with ^{99m}Tc and ^{188}Re 20

4.8.2 PBS stability with ^{99m}Tc, ^{188}Re and ^{64}Cu 20
4.8.3 Serum stability with ^{99m}Tc, ^{188}Re and ^{64}Cu 21
4.9 ^{99m}Tc uptake in DU145, DU145-PSMA+ and LNCaP cells 21
4.10 *In vivo* imaging and biodistribution (DU145-PSMA+ and LNCaP) 22
5 Stability studies – HPLC chromatograms and analysis 25
6 Conjugation screen – LC-MS chromatograms 33
7 Additional data from **DP^{An}** thiol reactions 37
8 NMR spectra 40
9 References 58

1 Abbreviations

**%AR**	percentage added radioactivity
**%ID**	percentage injected dose
**DP^{An}**	2,3-bis(di-*p*-anisylphosphino)maleic anhydride
**DP^{MEP}**	2,3-bis(di-*p*-(methoxyethoxy)phenylphosphino)maleic anhydride
**DP^{Ph}**	2,3-bis(di-*p*-phenylphosphino)maleic anhydride
**DP^{Tol}**	2,3-bis(di-*p*-tolylphosphino)maleic anhydride
**DTPA**	Diethylenetriaminepentaacetic acid
**EDDA**	ethylenediamine-*N*,*N*'-diacetic acid
**FAPi**	fibroblast activation protein inhibitor
**GlcN**	glucosamine
**GSH**	glutathione
**HYNIC**	6-hydrazinonicotinic acid
**MOE**	methoxyethyl
**PBS**	phosphate buffered saline
**PET**	positron emission tomography
**PET/CT**	positron emission tomography computed tomography
**PMPA**	2-phosphonomethyl pentanedioic acid
**PSMA**	prostate specific membrane antigen
**PSMAt**	prostate specific membrane antigen targeting
**RCP**	radiochemical purity
**RCY**	radiochemical yield
**SPECT**	single photon emission computed tomography
**SPECT/CT**	single photon emission computed tomography computed tomography

2 General experimental

All synthetic reactions of non-peptidic phosphines and molybdenum complexes were carried out under a pre-purified nitrogen atmosphere, where the solvents are stated as anhydrous or deoxygenated, using standard Schlenk line techniques. These air and moisture sensitive compounds were manipulated and stored in an Ar-atmosphere glovebox. DCM, Et_2O, hexane and THF were dried by Grubbs-type solvent purification system with activated alumina columns and deoxygenated by bubbling with N_2 for 30 min. Bottled EtOAc, $CDCl_3$ and CD_2Cl_2 were purchased from Sigma-Aldrich, deoxygenated by bubbling with N_2 for 30 min and dried over 4 Å molecular sieves. Other commercial reagents and chemicals were used without further purification unless otherwise stated. TLC visualisation was performed by examination under UV light (254 nm), potassium permanganate solution and/or ninhydrin solution. The PSMAt-NH_2 motif was purchased from Peptide Synthetics (Hampshire, UK). Both FAPi[1,2] and **DP^{Ph}-PSMAt**[3] were synthesised according to previously published procedures. Dichloromaleic anhydride was sublimated under reduced pressure (10^{-1} mbar) at 120 ˚C prior to use. [^{99m}Tc]pertechnetate in saline, eluted from a UTK generator (Curium), was supplied by the Guy's and St Thomas' Hospital Nuclear Medicine Services, London. Ammonium [^{99g}Tc]pertechnetate was supplied by Amersham International plc (Amersham UK, 1991).The ^{99g}Tc(V) precursor [N^{t}Bu$_4$][^{99g}TcOCl$_4$][4] was synthesised according to previously published procedures. [^{188}Re]perrhenate in saline, was eluted from a ^{188}W/^{188}Re OncoBeta generator. ^{64}Cu was produced by a ^{64}Ni(p,n)^{64}Cu nuclear reaction on a CTI RDS 112 11 MeV cyclotron and purified to give $^{64}Cu^{2+}$ in 0.1 M HCl solutions used for radiolabelling.[5,6]

NMR data were recorded on Jeol ECS300, Jeol ECS400, Jeol ECZ400 (Jastec or Varian magnet), Bruker Avance III 400, Varian VNMRS 500 or Bruker cryo500 MHz spectrometers. Chemical shifts (δ) are reported in parts per million (ppm) and coupling constants (*J*) in Hz. Chemical shifts for ^{1}H and ^{13}C spectra are referenced to residual solvent peaks, while ^{31}P NMR spectra are reported relative to 85% H_3PO_4 as an external standard. Two-dimensional NMR experiments were routinely used to confirm NMR assignments. Infrared spectra were recorded on a PerkinElmer FT-IR. High-resolution mass spectra were obtained by either: (a) the University of Bristol Mass Spectrometry Service using an ESI (Bruker Daltonics micrOTOF II), Nanospray (Waters Synapt G2S), or MALDI (Bruker ultrafleXtreme 2) spectrometer or (b) the King's College London, Department of Chemistry Mass Spectrometry Service, using a high resolution Thermo Exactive mass spectrometer in positive electrospray mode.

Purification by **preparative and semi-preparative reverse-phase HPLC** was performed on an Agilent Prostar HPLC with prepLC software (Agilent) or an Agilent 1260 Infinity II HPLC system with OpenLab (Agilent) software, a Rheodyne sample loop (2 or 5 mL) and monitoring *via* UV detection at 220, 254

and/or 280 nm wavelengths. Semi-preparative HPLC was performed using an Agilent Eclipse XDB-C_{18} column (9.4 x 250 mm, 5 µM particle size), with flow rate of 3 mL min^{-1}. Preparative HPLC was performed using an Agilent ZORBAX XDB-C_{18} column (21.2 x 150 mm, 5 µM particle size), with flow rate of 5 mL min^{-1}. **Analytical HPLC** was performed on an Agilent 1200 LC system with Laura software or an Agilent 1260 Infinity II HPLC system with Laura software, a Rheodyne sample loop (200 µL or 1 mL), using an analytical Agilent Zorbax Eclipse XDB-C_{18} column (4.6 x 150 mm, 5 µm particle size), with flow rate 1 mL min^{-1} and monitoring by UV detection at 220, 254 and/or 280 nm. Size-exclusion (SEC) HPLC was performed using a BioSep^{TM} SEC-s2000 LC Column (300 x 7.8 mm, 5 µm, 145 Å, Phenomenex®). For radioactivity detection, a LabLogic Flow-Count detector with a NaI probe (B-FC-3200) or a Raytest Gabi detector with a NaI probe was attached to the HPLC. **LC-MS** was performed using an Agilent 1260 Infinity II HPLC coupled to an Advion Expression LC-MS Mass Spectrometer with Advion Data and Mass Express software, using an analytical Agilent Zorbax Eclipse XDB-C_{18} column (4.6 x 150 mm, 5 µm particle size), with flow rate 1 mL min^{-1} and monitoring by a low-resolution MS-ESI probe and UV detection at 200–330 nm. Specific methods are described below with mobile phases for reverse-phase chromatography using solvent A (H_2O) and solvent B (MeCN), both containing trifluoroacetic acid (TFA), acetic acid (AA) or formic acid (FA). Size-exclusion chromatography used isocratic phosphate-buffered saline (PBS) as the mobile phase.

Method 1 (C_{18}-semi-preparative, 0.01% AA, 254 nm, 3 mL min^{-1} flow rate): 50 min, 0–5 min at 5% B, linear increase to 75% B at 40 min, linear increase to 95% B at 50 min. **Method 2** (C_{18}-analytical, 0.1% TFA, 220 nm, 1 mL min^{-1} flow rate): 20 min, 0–20 min linear increase from 0–100% B. **Method 3** (SEC, PBS, 280 nm, 1 mL min^{-1} flow rate) 30 min, isocratic gradient. **Method 4** (C_{18}-analytical, 0.1% TFA, 220 nm, 1 mL min^{-1} flow rate): 30 min, 0–30 min linear increase from 0–100% B. **Method 5** (C_{18}-analytical, 0.1% TFA, 220 nm, 1 mL min^{-1} flow rate): 0–60 min linear increase from 0–60% B.

LC-MS 15 min (C_{18}-analytical, 0.1% FA, 220, 254 and 280 nm, 1 mL min^{-1} flow rate): 5% solvent B at 0–2 min, linear increase to 95% solvent B at 11 min, linear decrease back to 5% solvent B at 12 min, then 5% solvent B at 12–15 min. **LC-MS 20 min** (C_{18}-analytical, 0.1% FA, 220, 254 and 280 nm, 1 mL min^{-1} flow rate): 20 min, 0–20 min linear increase from 0–100% B. **LC-MS 40 min** (C_{18}-analytical, 0.1% FA, 220, 254 and 280 nm, 1 mL min^{-1} flow rate): 0–5 min at 0% B, linear increase to 100% B at 35 min, 35–40 min at 100% B.

iTLC: Instant thin layer chromatography (iTLC) used iTLC SGI0001 strips (Varian Medical Systems, Crawley, UK). The iTLC plates were scanned with a LabLogic miniScan TLC reader equipped with Laura software. Two separate iTLC analyses were used to enable quantification of ^{99m}Tc-colloids, unreacted $^{99m}TcO_4^-$ and [$^{99m}TcO_2$(**DP-peptide**)$_2$]$^+$. To quantify amounts of unreacted $^{99m}TcO_4^-$, acetone was used

as a mobile phase with R_f values: $^{99m}TcO_4^-$ >0.9, ^{99m}Tc-colloids <0.1, [$^{99m}TcO_2$(**DP-peptide**)$_2$]$^+$ <0.1. To quantify ^{99m}Tc-colloid formation, a 1:1 mixture of methanol and 2 M aqueous ammonium acetate solution was used as a mobile phase with R_f values: $^{99m}TcO_4^-$ >0.9, [$^{99m}TcO_2$(**DP-peptide**)$_2$]$^+$ >0.9, ^{99m}Tc-colloids <0.1. Analogous iTLC conditions were also used for [$^{188}ReO_2$(**DP-peptide**)$_2$]$^+$ radiotracers. **^{188}Re-citrate:** To quantify the RCY of ^{188}Re-citrate intermediate, acetone was used as a mobile phase with R_f values: $^{188}ReO_4^-$ >0.9, ^{188}Re-colloids <0.1, ^{188}Re-citrate <0.1. To quantify ^{188}Re-colloid formation, 0.9% saline solution was used as a mobile phase with R_f values: $^{99m}TcO_4^-$ >0.9, ^{188}Re-citrate >0.9, ^{99m}Tc-colloids <0.1.

Throughout this work, complexes of the general type [MO_2(**DP-peptide**)$_2$]$^+$, where M = Re or Tc, *cis* and *trans* refers to the geometry of the diphosphine ligands, not the *trans*-MO_2 dioxo core.

3 Experimental procedures

3.1 Bis(*para*-methoxyphenyl)phosphine

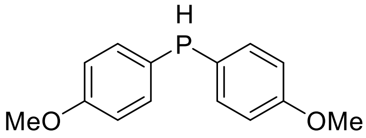

Under an inert atmosphere, a solution of bis(4-methoxyphenyl)chlorophosphine (1.00 g, 3.56 mmol) in anhydrous Et_2O (4.5 mL) was added dropwise to a suspension of $LiAlH_4$ (1.24 g, 11.4 mmol, 3.2 equiv.) in anhydrous Et_2O (18 mL) at 0 °C. The solution was stirred for a further 30 min at 0 °C before being allowed to warm to ambient temperature and then stirred overnight. The reaction mixture was cooled to 0 °C and quenched by the careful addition of deoxygenated: H_2O (0.5 mL), 15% NaOH in H_2O (0.5 mL) then H_2O (2.5 mL). After stirring for 1 h, the solution was isolated by filtration and then concentrated *in vacuo* to give the title compound (744 mg, 3.02 mmol, 85%) as a white solid. **$^{31}P\{^1H\}$ NMR** (162 MHz, $CDCl_3$): δ_P (ppm) –44.2 (s). **^1H NMR** (400 MHz, $CDCl_3$): δ_H (ppm) 7.46–7.28 (m, 4H, Ar-*H*), 6.90–6.82 (m, 4H, Ar-*H*), 5.38–4.98 (br. s, P*H*), 3.80 (s, 6H, OMe). The spectroscopic data are in accordance with the literature.[7]

3.2 2,3-Bis(bis(*p*-$MeOC_6H_4$)phosphino)maleic anhydride, DP^{An}

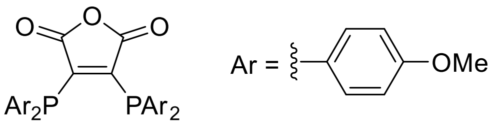

Under an inert atmosphere, NEt_3 (30.0 µL, 0.218 mmol, 2.2 equiv.) was added to a solution of Ar_2PH (50.0 mg, 0.203 mmol, 2.05 equiv.) in anhydrous Et_2O (0.5 mL). A solution of 2,3-dichloromaleic anhydride (16.5 mg, 98.8 µmol) in anhydrous Et_2O (0.5 mL) was added dropwise, which resulted in an immediate colour change from colourless to deep red solution. Once the reaction had reached completion, as monitored by ^{31}P NMR spectroscopy, the crude product was passed through a silica plug eluting with anhydrous EtOAc and concentrated *in vacuo* to give the title compound (51.4 mg, 87.7 µmol, 89%) as an orange solid. Any residual secondary phosphine was removed under high vacuum (*ca.* $\times 10^{-7}$ Torr). **$^{31}P\{^1H\}$ NMR** (122 MHz, $CDCl_3$): δ_P (ppm) –22.4 (s). **^1H NMR** (400 MHz, $CDCl_3$): δ_H (ppm) 7.28–7.21 (m, 8H, Ar-*H*), 6.83–6.78 (m, 8H, Ar-*H*), 3.80 (s, 12H, OMe). **^{13}C NMR** (101 MHz, $CDCl_3$): δ_C (ppm) 163.0 (m, *C*=O), 161.1 (s, *p*-Ar*C*), 154.2 (m, *C*=*C*), 135.9 (t, $^2J_{P,C}$ = 12.2, *o*-ArCH), 123.4 (s, Ar*C*), 114.5 (t, $^3J_{P,C}$ = 4.9 Hz, *m*-Ar*C*H), 55.3 (s, OMe). **HR-MS** (Nanospray): *m/z* calcd. for $C_{32}H_{29}O_7P_2$ $[M+H]^+$ = 587.1389; obs. = 587.1395.

3.3 4-Bromo-(2-methoxyethoxy)benzene

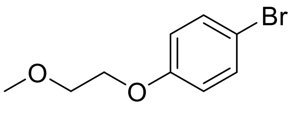

The title compound (6.01 g, 26.0 mmol, 90%) was synthesised as a colourless oil according to the literature procedure.[8] **^1H NMR** (400 MHz, $CDCl_3$): δ_H (ppm) 7.45–7.29 (m, 2H, Ar-*H*), 6.96–6.59 (m, 2H, Ar-*H*), 4.21–4.01 (m, 2H, CH_2), 3.90–3.67 (m, 2H, CH_2), 3.80 (s, 12H, OMe). **^{13}C NMR** (101 MHz, $CDCl_3$): δ_C (ppm)

158.0, 132.3, 116.5, 113.2, 71.0, 67.6, 59.3. The spectroscopic data are in accordance with the literature.[9]

3.4 (Diethylamino)bis(*para*-(2-methoxyethoxy)phenyl)phosphine

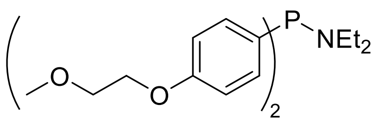

Under an inert atmosphere, 1-bromo-4-(2-methoxyethoxy)benzene (approx. 10% of 1.39 g, 6.03 mmol, 2.1 eq), anhydrous THF (5 mL) and a crystal of iodine were added to magnesium turnings (157 mg, 6.32 mmol, 2.2 equiv.) and the mixture was heated to reflux. A solution of the remaining 1-bromo-4-(2-methoxyethoxy)benzene in anhydrous THF (5 mL) was added dropwise whilst maintaining reflux followed by further addition of anhydrous THF (18 mL). After 2 h, the reaction mixture was allowed to cool to room temperature and then added dropwise over 1 h to a cooled solution of $P(NEt_2)Cl_2$ (500 mg, 2.87 mmol) in anhydrous THF (5 mL) at 0 °C. After stirring overnight, the reaction mixture was concentrated and dissolved in anhydrous hexane (approx. 10 mL). The product was then isolated by cannula filtration followed by concentration *in vacuo* to give the title compound (785 mg, 80% purity, 1.55 mmol, 54% yield) as a colourless oil with 2-methoxyethoxybenzene contaminant (20%). **$^{31}P\{^1H\}$ NMR** (162 MHz, $CDCl_3$): δ_P (ppm) +60.1 (s). **^1H NMR** (400 MHz, $CDCl_3$): δ_H (ppm) 7.36–7.20 (m, 4H, Ar-*H*), 6.99–6.81 (m, 4H, Ar-*H*), 4.25–3.99 (m, 4H, C*H*$_2$CH$_2$OMe), 3.81–3.60 (m, 4H, C*H*$_2$OMe), 3.44 (s, 6H, OMe), 3.02 (dq, 4H, $^3J_{P,H}$ = 9.7 Hz, $J_{H,H}$ = 7.0 Hz, NC*H*$_2$CH$_3$), 0.91 (t, 6H, $J_{H,H}$ = 7.0 Hz, NCH$_2$C*H*$_3$). **^{13}C NMR** (101 MHz, $CDCl_3$): δ_C (ppm) 159.1 (s, *p*-Ar*C*), 133.5 (d, $^2J_{P,C}$ = 20.8 Hz, *o*-Ar*C*H), 132.3 (d, $^1J_{P,C}$ = 12.0 Hz, Ar*C*), 114.4 (d, $^3J_{P,C}$ = 6.5 Hz, *m*-Ar*C*H), 71.1 (s, *C*H$_2$OMe), 67.3 (s, *C*H$_2$CH$_2$OMe), 59.3 (s, OMe), 44.1 (d, $^2J_{P,C}$ = 15.5 Hz, N*C*H$_2$CH$_3$), 14.6 (d, $^3J_{P,C}$ = 3.1 Hz, NCH$_2$*C*H$_3$). **HR-MS** (Nanospray): *m/z* calcd. for $C_{22}H_{33}NO_5P$ $[M+O+H]^+$ = 422.2096; obs. = 422.2095.

2-methoxyethoxybenzene: ^1H NMR (400 MHz, $CDCl_3$): δ_H (ppm) 7.36–7.20 (m, 2H), 6.99–6.81 (m, 3H), 4.25–3.99 (m, 2H), 3.81–3.60 (m, 2H), 3.44 (s, 3H). **^{13}C NMR** (101 MHz, $CDCl_3$): δ_C (ppm) 158.9 (s), 129.5 (s), 121.0 (s), 114.7 (s), 71.2 (s), 67.3 (s), 59.3 (s). The spectroscopic data are in accordance with the literature.[10]

3.5 Bis(*para*-(2-methoxyethoxy)phenyl)chlorophosphine

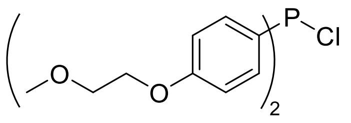

HCl (3.08 mL, 2 M in Et_2O) was added dropwise to a solution of Ar_2PNEt_2 (1.00 g, 2.46 mmol) in anhydrous Et_2O (40 mL) at −78 °C. The reaction mixture was then allowed to warm to RT for 30 min and the title compound was then isolated by cannula filtration and concentrated *in vacuo*. This material was then used directly in the reduction to the secondary phosphine. **$^{31}P\{^1H\}$ NMR** (122 MHz, Et_2O): δ_P (ppm) +84.6 (s).

3.6 Bis(*para*-(2-methoxyethoxy)phenyl)phosphine

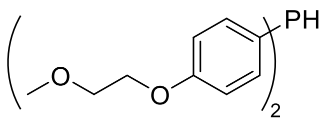

A solution of Ar_2PCl (assumed 2.46 mmol) in anhydrous Et_2O (5 mL) was added dropwise to a suspension of $LiAlH_4$ (94.0 mg, 2.46 mmol) in anhydrous Et_2O (20 mL) at 0 °C. After stirring at RT for 2.5 h, the reaction mixture was cooled to 0 °C, quenched by careful addition of deoxygenated H_2O (8 mL) and stirred for a further 30 min. The product was isolated by cannula filtration and concentrated *in vacuo* to give the title compound (762 mg, 90% purity, 2.28 mmol, 83% yield over two steps) as a colourless oil. Both contaminants (δ_P 21.1 ppm and δ_P −124.5 ppm) were removed upon purification at the next step. **$^{31}P\{^1H\}$ NMR** (162 MHz, $CDCl_3$): δ_P (ppm) −44.2 (s). **^1H NMR** (400 MHz, $CDCl_3$): δ_H (ppm) 7.36–7.30 (m, 4H, Ar-*H*), 6.87–6.81 (m, 4H, Ar-*H*), 5.13 (d, 1H, $^1J_{P,H}$ = 218.6 Hz, P*H*), 4.08–4.04 (m, 4H, C*H*$_2$CH$_2$OMe), 3.72–3.67 (m, 4H, C*H*$_2$OMe), 3.40 (s, 6H, OMe). **^{13}C NMR** (101 MHz, $CDCl_3$): δ_C (ppm) 159.4 (s, *p*-Ar*C*), 135.51 (d, $J_{P,C}$ = 18.3 Hz, Ar*C*H), 126.1 (d, $^1J_{P,C}$ = 7.2 Hz, Ar*C*), 115.0 (d, $J_{P,C}$ = 7.0 Hz, Ar*C*H), 71.0 (s, *C*H$_2$OMe), 67.3 (s, *C*H$_2$CH$_2$OMe), 59.3 (s, OMe). **HR-MS** (Nanospray): *m/z* calcd. for $C_{18}H_{24}O_5P$ $[M+O+H]^+$ = 351.1361; obs. = 351.1352.

3.7 2,3-Bis(bis(*p*-MeOCH$_2$CH$_2$OC$_6$H$_4$)phosphino)maleic anhydride, DP^{MEP}

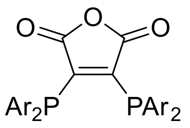

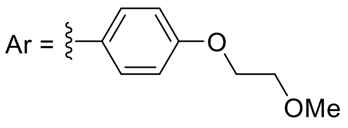

Under an inert atmosphere, NEt_3 (190 µL, 1.39 mmol, 2.2 equiv.) was added to a solution of Ar_2PH (678 mg at 90% purity, 1.83 mmol, 2.9 equiv.) in anhydrous Et_2O (6 mL). A solution of 2,3-dichloromaleic anhydride (105.8 mg, 634 µmol) in anhydrous Et_2O (0.5 mL) was added dropwise, which resulted in an immediate colour change from colourless to deep red solution. After 2 h, the reaction mixture was passed through a silica plug eluting with anhydrous EtOAc, concentrated to *ca.* 1 mL and precipitated over hexane (*ca.* 30 mL) to give the title compound (294 mg, 385 µmol, 61%) as an orange solid. **$^{31}P\{^1H\}$ NMR** (162 MHz, $CDCl_3$): δ_P (ppm) –22.3 (s). **^1H NMR** (400 MHz, $CDCl_3$): δ_H (ppm) 7.25–7.19 (m, 4H, *m*-Ar-*H*), 6.85–6.80 (m, 4H, *o*-Ar-*H*), 4.12–4.08 (m, 4H, C*H*$_2$CH$_2$OMe), 3.76–3.72 (m, 4H, C*H*$_2$OMe), 3.44 (s, 6H, OMe). **^{13}C NMR** (101 MHz, $CDCl_3$): δ_C (ppm) 163.0 (m, *C*=O), 160.4 (s, *p*-Ar*C*), 154.1 (m, *C*=*C*), 135.9 (t, $^3J_{P,C}$ = 12.2 Hz, *o*-Ar*C*H), 123.7 (s, Ar*C*), 115.1 (t, $^2J_{P,C}$ = 4.8 Hz, *m*-Ar*C*H), 71.0 (s, *C*H$_2$OMe), 67.4 (s, *C*H$_2$CH$_2$OMe), 59.4 (s, OMe). **HR-MS** (Nanospray): *m/z* calcd. for $C_{40}H_{45}O_{11}P_2$ $[M+H]^+$ = 763.2437; obs. = 763.2450.

3.8 DP^{An}-PSMAt

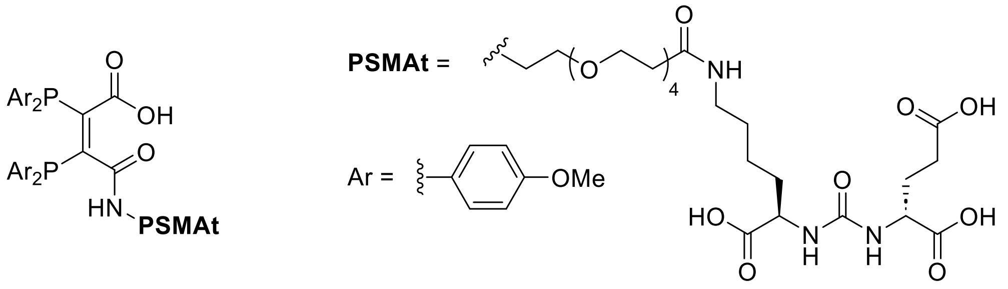

A solution of PSMAt-NH$_2$ (10.0 mg, 17.6 µmol) in dry, deoxygenated DMF (200 µL) was added to **DP^{An}** (10.4 mg, 17.6 µmol) in dry, deoxygenated DMF (200 µL) and mixed well. DIPEA (10.0 µL) was then added in two portions, the solution flushed with N_2 and agitated at room temperature for 15–20 min. The product was then isolated by semi-preparative HPLC (Method 1, t_R = 32 min), neutralising product fractions upon elution with 0.125 M ammonium bicarbonate buffer (15 µL per mL eluant). Purified fractions were lyophilised to give the title compound (7.0 mg, 6.07 µmol, 34%) as an off-white solid. **^{31}P{^{1}H} NMR** (162 MHz, DMF-d_7): δ_P (ppm) −16.9 (d, $^3J_{P,P}$ = 139.8 Hz), −18.9 (d, $^3J_{P,P}$ = 139.8 Hz). **^{1}H NMR** (400 MHz, DMF-d_7 + DIPEA): δ_H (ppm) 7.99 (m, 1H), 7.77 (t, 1H, $J_{H,H}$ = 5.7 Hz), 7.48–7.33 (m, 8H), 6.85–6.71 (m, 8H), 6.63 (d, 1H, $J_{H,H}$ = 7.9 Hz), 6.58 (d, 1H, $J_{H,H}$ = 7.4 Hz), 4.35–4.22 (m, 2H), 3.79 (s, 6H), 3.77 (s, 6H), 3.69 (t, 2H, $J_{H,H}$ = 6.2 Hz), 3.61–3.51 (m, 10H), 3.43–3.39 (m, 2H), ≈3.14 (hidden, 2H), 2.99–2.95 (m, 4H), 2.52–2.36 (m, 4H), 2.05–1.97 (m, 2H), 1.82–1.74 (m, 1H), 1.71–1.61 (m, 1H), 1.53–1.37 (m, 4H). **LC-MS** (ESI+): 15 min method, t_R = 9.6 min; *m/z* calcd. for $C_{55}H_{71}N_4O_{19}P_2$ [*M*+H]$^+$ = 1153.4; obs. = 1153.6. **HR-MS** (ESI+): *m/z* calcd. for $C_{55}H_{71}N_4O_{20}P_2$ [*M*+O+H]$^+$ = 1169.4137; obs. = 1169.4110.

3.9 DP^{MEP}-PSMAt

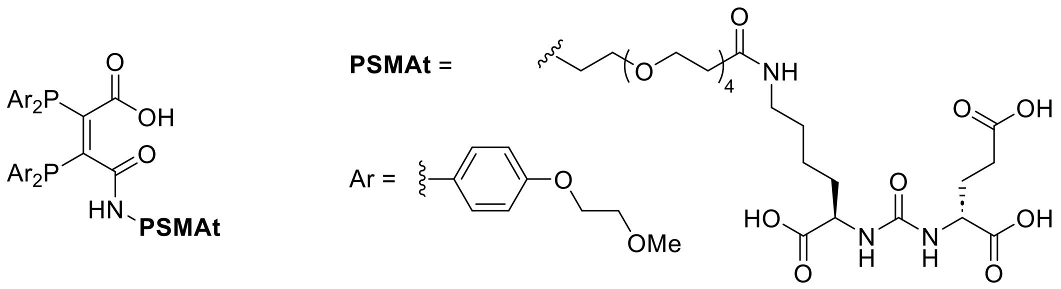

A solution of PSMAt-NH$_2$ (4.83 mg, 8.52 µmol) in dry, deoxygenated DMF (200 µL) was added to **DP^{MEP}** (6.50 mg, 8.52 µmol) in dry, doxygenated DMF (200 µL) and mixed well. DIPEA (5.0 µL) was then added in two portions, the solution flushed with N_2 and agitated at room temperature for 15–20 min. The product was then isolated by semi-preparative HPLC (Method 1, t_R = 31 min), neutralising product fractions upon elution with 0.125 M ammonium bicarbonate buffer (15 µL per mL eluant). Purified fractions were lyophilised to give the title compound (4.2 mg, 3.16 µmol,37%) as an off-white solid.

$^{31}P\{^1H\}$ NMR (162 MHz, DMF-d_7): δ_P (ppm) −16.7 (d, $^3J_{P,P}$ = 142.6 Hz), −18.5 (d, $^3J_{P,P}$ = 142.6 Hz). **^{1}H NMR** (400 MHz, DMF-d_7 + DIPEA): δ_H (ppm) 7.87 (m, 1H), 7.78 (t, 1H, $J_{H,H}$ = 5.5 Hz), 7.46–7.40 (m, 4H), 7.39–7.33 (m, 4H), 6.83–6.74 (m, 8H), 6.61 (d, 1H, $J_{H,H}$ = 6.6 Hz), 6.55 (d, 1H, $J_{H,H}$ = 7.3 Hz), 4.33–4.21 (m, 2H), 4.15–4.07 (m, 8H), 3.75–3.66 (m, 10H), 3.59–3.50 (m, 10H), 3.43–3.39 (m, 2H), 3.37 (2 x s, 12H), ≈3.16 (hidden, 2H), 3.00–2.95 (m, 4H), 2.50–2.36 (m, 4H), 2.06–1.93 (m, 2H), 1.85–1.73 (m, 1H), 1.71–1.60 (m, 1H), 1.53–1.38 (m, 4H). **LC-MS** (ESI+): 15 min method, t_R = 9.8 min; *m/z* calcd. for $C_{63}H_{87}N_4O_{23}P_2$ $[M+H]^+$ = 1329.5; obs. = 1329.0. **HR-MS** (ESI): *m/z* calcd. for $C_{63}H_{87}N_4O_{24}P_2$ $[M+O+H]^+$ = 1345.5180; obs. = 1345.5160.

3.10 Conjugation screen general procedure

DIPEA (2.5 µL) was added to amine (1.70 µmol) in anhydrous DMSO (100 µL). **DP^{An}** or **DP^{MEP}** (1.70 µmol) in anhydrous DMSO (100 µL) was then added, and the mixture was agitated for 10 min at ambient temperature before analysing an aliquot by LC-MS.

3.11 DP^{An} thiol reaction general procedure

The thiol (8.52 µmol) in anhydrous DMF-d$_7$ (500 µL) was added to a solution of **DP^{An}** (5 mg, 8.52 µmol) in anhydrous DMF-d$_7$ (500 µL) with/without anhydrous DIPEA (2.2 µL). The solution was then de-gassed with argon, and then 700 µL was transferred to an NMR tube under an argon atmosphere. An aliquot was also taken for analysis by LC-MS (15 min method).

3.12 Mo(0) coordination general procedure

Diphosphine (42.6 µmol, 1 equiv.) was dissolved in anhydrous DCM (0.5 mL) under N_2 and added dropwise to a solution of $[Mo(CO)_4(nbd)]$ (1 equiv.) in anhydrous DCM (0.5 mL). After the complexation had reached completion, as monitored by $^{31}P\{^1H\}$ NMR spectroscopy (approx. 2 h), hexane (2 mL) was added to afford a precipitate. The supernatant was removed, the precipitate washed with hexane (3 x 1 mL) and then dried *in vacuo*.

3.12.1 *cis*-[Mo(CO)$_4$(**DP^{Ph}**)]

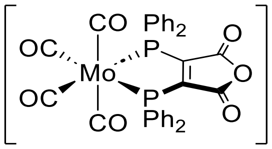

The title compound (29.9 mg, 44.3 µmol, 69%) was isolated as a purple solid. **$^{31}P\{^1H\}$ NMR** (162 MHz, $CDCl_3$): δ_P (ppm) 51.7 (s). **^{1}H NMR** (400 MHz, $CDCl_3$): δ_H (ppm) 7.63–7.55 (m, 8H, Ar-*H*), 7.53–7.43 (m, 12H, Ar-*H*). **IR** (CH_2Cl_2, 1 mg mL^{-1}): ν(CO) = 2031, ~1939, 1920 cm^{-1}, ν(C=O anhydride) = 1775 cm^{-1}. **HR-MS was unable to be obtained, however, it has been obtained for the subsequent ring-opened*

*complex. *Shoulder peak at 1939 cm^{-1} was calculated manually.* The spectroscopic data are in accordance with the literature.[3]

3.12.2 *cis*-[Mo(CO)$_4$(**DP**^{An})]

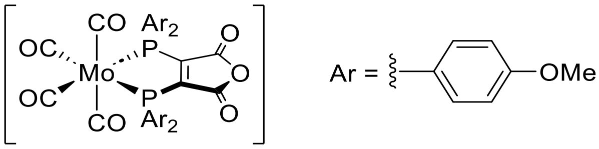

The title compound (29.7 mg, 37.4 µmol, 88%) was isolated as a dark brown solid. **^{31}P{^{1}H} NMR** (162 MHz, CDCl$_3$): δ_P (ppm) 49.0 (s). **^{1}H NMR** (400 MHz, CDCl$_3$): δ_H (ppm) 7.55–7.48 (m, 8H, Ar-*H*), 6.99–6.94 (m, 8H, Ar-*H*), 3.85 (s, 12H, OMe). **^{13}C{^{1}H} NMR** (126 MHz, CD$_2$Cl$_2$): δ_C (ppm) 215.9–215.5 (m, Mo-*C*O$_{eq}$), 209.1 (t, $^2J_{P,C}$ = 8.6 Hz, Mo-*C*O$_{ax}$), 165.5–165.1 (m, *C*=O), 162.4 (s, *p*-Ar*C*), 159.6–159.1 (m, *C*=*C*), 134.8–134.7 (t, $^2J_{P,C}$ = 7.7 Hz, *o*-Ar*C*H), 123.3–122.9 (m, Ar*C*), 114.9 (t, $^3J_{P,C}$ = 5.7 Hz, *m*-Ar*C*H), 55.8 (s, OMe). **HR-MS** (Nanospray): *m/z* calcd. for $C_{36}H_{28}O_{11}P_2{}^{98}Mo$ [*M*+O$_2$+H]$^+$ = 829.0137; obs. = 829.0147. **IR** (CH$_2$Cl$_2$, 1 mg mL^{-1}): ν(CO) = 2028, ~1932, 1915 cm^{-1}, ν(C=O anhydride) = 1773 cm^{-1}. **Shoulder peak at 1932 cm^{-1} was calculated manually.*

3.12.3 *cis*-[Mo(CO)$_4$(**DP**^{MEP})]

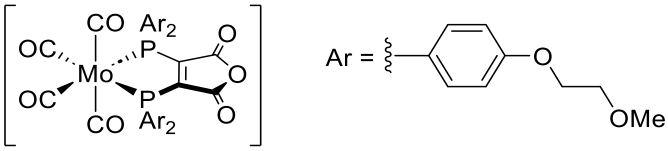

The title compound (45.3 mg, 46.7 µmol, 89%) was isolated as a dark brown solid. **^{31}P{^{1}H} NMR** (162 MHz, CDCl$_3$): δ_P (ppm) 48.9 (s). **^{1}H NMR** (400 MHz, CDCl$_3$): δ_H (ppm) 7.52–7.44 (m, 8H, Ar-*H*), 7.00–6.94 (m, 8H, Ar-*H*), 4.18–4.12 (m, 8H, C*H*$_2$), 3.79–3.72 (m, 8H, C*H*$_2$), 3.45 (s, 12H, OMe). **^{13}C{^{1}H} NMR** (126 MHz, CD$_2$Cl$_2$): δ_C (ppm) 215.8–215.4 (m, Mo-*C*O$_{eq}$), 209.0 (t, $^2J_{P,C}$ = 8.6 Hz, Mo-*C*O$_{ax}$), 165.4–165.1 (m, *C*=O), 161.6 (s, *p*-Ar*C*), 159.5–159.1 (m, *C*=*C*), 134.7 (t, $^2J_{P,C}$ = 7.7 Hz, *o*-Ar*C*H), 123.5–123.1 (m, Ar*C*), 115.4 (t, $^3J_{P,C}$ = 5.7 Hz, *m*-Ar*C*H), 71.1 (s, *C*H$_2$OMe), 68.0 (s, *C*H$_2$CH$_2$OMe), 59.3 (s, OMe). **HR-MS** (Nanospray): *m/z* calcd. for $C_{44}H_{44}O_{15}P_2MoNa$ [*M*+Na]$^+$ = 995.1107; obs. = 995.1132. **IR** (CH$_2$Cl$_2$, 1 mg mL^{-1}): ν(CO) = 2028, ~1932, 1915 cm^{-1}, ν(C=O anhydride) = 1773 cm^{-1}. **Shoulder peak at 1932 cm^{-1} was calculated manually.*

3.13 Ring-opening of Mo(0) anhydride complexes general procedure

2-Methoxyethylamine (MOE-NH$_2$, 25.2 µmol, 1 equiv.) was added to [Mo(CO)$_4$(**DP**)] (1 equiv.) in anhydrous DCM (1 mL). After 1 h hexane (2 mL) was added, affording a precipitate. The supernatant was removed, the precipitate washed with hexane (3 x 1 mL) and then dried *in vacuo*.

3.13.1 [MOE-NH$_3$][Mo(CO)$_4$(DP^{Ph}-NH-MOE)]

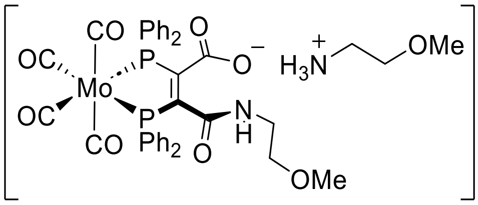

The title compound (16.7 mg, 20.2 µmol, 91%) was isolated as a yellow solid. **^{31}P{^{1}H} NMR** (162 MHz, CD_2Cl_2): δ_P (ppm) 72.6 (d, $J_{P,P}$ = 3.8 Hz), 70.2 (d, $J_{P,P}$ = 3.8 Hz). **^{1}H NMR** (400 MHz, $CDCl_3$): δ_H (ppm) 7.73–7.67 (m, 8H, Ar-*H*), 7.39–7.29 (m, 12H, Ar-*H*), 6.16 (br. s, 1H, NH), 3.33–3.28 (m, 2H, C*H*$_2$), 3.24 (s, 3H, OMe), 3.07 (s, 3H, OMe), 3.00–2.94 (m, 4H, C*H*$_2$), 2.71–2.66 (m, 2H, C*H*$_2$). **HR-MS** (Nanospray): *m/z* calcd. for $C_{35}H_{28}NO_8P_2{}^{98}Mo$ [*M*−H]$^-$ = 750.0344; obs. = 750.0355. **IR** (CH_2Cl_2, 1 mg mL^{-1}): ν(CO) = 2024, ~1931, 1902 cm^{-1}. **Shoulder peak at 1931 cm^{-1} was calculated manually.* The spectroscopic data were in accordance with the literature.[3]

3.13.2 [MOE-NH$_3$][Mo(CO)$_4$(DP^{An}-NH-MOE)]

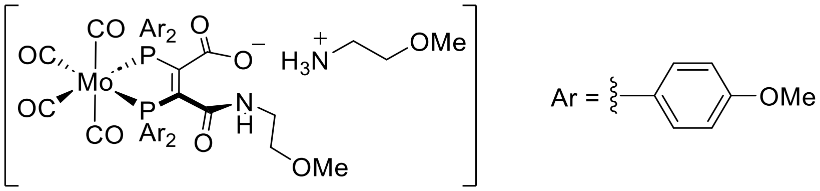

The title compound (21.9 mg, 23.2 µmol, 92%) was isolated as a pale brown solid. **^{31}P{^{1}H} NMR** (162 MHz, CD_2Cl_2): δ_P (ppm) 69.3 (s), 67.4 (s). **^{1}H NMR** (400 MHz, CD_2Cl_2): δ_H (ppm) 7.63–7.47 (m, 8H, Ar-*H*), 6.92–6.77 (m, 8H, Ar-*H*), 5.88 (br. s, 1H, NH), 3.80 (s, 6H, *p*-OMe), 3.77 (s, 6H, *p*-OMe), 3.34 (broad m, 2H, C*H*$_2$), 3.23 (br. s, 3H, OMe), 3.07 (s, 3H, OMe), 3.03–2.97 (m, 4H, C*H*$_2$), 2.77 (broad m, 2H, C*H*$_2$). **^{13}C{^{1}H} NMR** (126 MHz, CD_2Cl_2): δ_C (ppm) 218.0–217.6 (m, Mo-*C*O$_{eq}$), 209.7 (t, $^2J_{P,C}$ = 8.6 Hz, Mo-*C*O$_{ax}$), 171.4 (d, $J_{P,C}$ = 20.2 Hz, *C*=O), 167.7 (d, $J_{P,C}$ = 20.6 Hz, *C*=O′), 161.5 (d, $^4J_{P,C}$ = 1.6 Hz, *p*-Ar*C*), 161.3 (d, $^4J_{P,C}$ = 1.6 Hz, *p*-Ar*C*′), 135.0 (d, $^2J_{P,C}$ = 15.1 Hz, *o*-Ar*C*H), 134.7 (d, $^2J_{P,C}$ = 14.9 Hz, *o*-Ar*C*H′), 133.0 (d, $J_{P,C}$ = 13.1 Hz, *C*=C), 130.2 (d, $J_{P,C}$ = 24.7 Hz, *C*=C′), 127.5 (dd, $J_{P,C}$ = 40.7, 2.1 Hz, Ar*C*), 126.4 (dd, $J_{P,C}$ = 39.9, 2.3 Hz, Ar*C*′), 114.0 (d, $^3J_{P,C}$ = 10.7 Hz, *m*-Ar*C*H), 113.8 (d, $^3J_{P,C}$ = 10.9 Hz, *m*-Ar*C*H′), 70.7 (s, *C*H$_2$OMe), 68.3 (s, *C*H$_2$OMe′), 59.0 (s, OMe$_{alkyl}$), 58.5 (s, OMe$_{alkyl}$′), 55.7 (s, OMe$_{aryl}$), 55.6 (s, OMe$_{aryl}$′), 39.6 (s, *C*H$_2$CH$_2$OMe), 39.6 (s, *C*H$_2$CH$_2$OMe′). **HR-MS** (Nanospray): *m/z* calcd. for $C_{39}H_{36}NO_{12}P_2{}^{98}Mo$ [*M*−H]$^-$ = 870.0782; obs. = 870.0767. **IR** (CH_2Cl_2, 1 mg mL^{-1}): ν(CO) = 2023, ~1928, 1899 cm^{-1}. **Shoulder peak at 1928 cm^{-1} was calculated manually.*

3.13.3 [MOE-NH$_3$][Mo(CO)$_4$(DP^{MEP}-NH-MOE)]

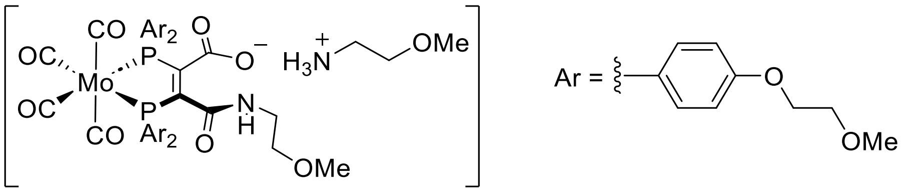

The title compound (20.7 mg, 18.5 µmol, 95%) was isolated as a pale brown solid. **^{31}P{^{1}H} NMR** (162 MHz, CD_2Cl_2): δ_P (ppm) 69.1 (d, $J_{P,P}$ = 2.7 Hz), 66.4 (d, $J_{P,P}$ = 2.7 Hz). **^{1}H NMR** (400 MHz, CD_2Cl_2): δ_H (ppm) 7.65–7.47 (m, 8H, Ar-*H*), 6.90–6.81 (m, 8H, Ar-*H*), 6.10 (br. s, 1H, NH), 4.12–4.03 (m, 8H, C*H*$_2$), 3.74–3.65 (m, 8H, C*H*$_2$), 3.40 (s, 6H, *p*-OMe), 3.38 (s, 6H, *p*-OMe), 3.31 (broad m, 2H, C*H*$_2$), 3.22 (br. s, 3H, OMe), 3.08 (s, 3H, OMe), 3.04–2.96 (m, 4H, C*H*$_2$), 2.71 (broad m, 2H, C*H*$_2$). **^{13}C{^{1}H} NMR** (126 MHz, CD_2Cl_2): δ_C (ppm) 218.3–217.8 (m, Mo-*C*O$_{eq}$), 209.8k (t, $^2J_{P,C}$ = 8.6 Hz, Mo-*C*O$_{ax}$), 172.0 (d, $J_{P,C}$ = 19.1 Hz, *C*=O), 168.0 (d, $J_{P,C}$ = 21.5 Hz, *C*=O′), 160.5 (s, *p*-Ar*C*), 160.4 (s, *p*-Ar*C*′), 135.2 (d, $^2J_{P,C}$ = 14.9 Hz, *o*-Ar*C*H), 134.8 (d, $^2J_{P,C}$ = 14.8 Hz, *o*-Ar*C*H′), 132.9 (d, $J_{P,C}$ = 12.9 Hz, *C*=C), 130.0 (s, *C*=C′), 128.2 (d, $J_{P,C}$ = 40.3 Hz, Ar*C*), 127.5 (d, $J_{P,C}$ = 39.4 Hz, Ar*C*′), 114.4 (d, $^3J_{P,C}$ = 10.7 Hz, *m*-Ar*C*H), 114.2 (d, $^3J_{P,C}$ = 10.8 Hz, *m*-Ar*C*H′), 71.2 (2 x s, *C*H$_2$OMe$_{aryl}$), 70.9 (s, *C*H$_2$OMe$_{alkyl}$), 68.4 (s, *C*H$_2$OMe$_{alkyl}$′), 67.7 (2 x s, *C*H$_2$CH$_2$OMe$_{aryl}$), 59.3 (s, OMe$_{aryl}$), 59.2 (s, OMe$_{aryl}$′), 58.9 (s, OMe$_{alkyl}$), 58.5 (s, OMe$_{alkyl}$′), 39.5 (s, *C*H$_2$CH$_2$OMe$_{alkyl}$), 39.4 (s, *C*H$_2$CH$_2$OMe$_{alkyl}$′). **HR-MS** (Nanospray): *m/z* calcd. for $C_{47}H_{52}NO_{16}P_2Mo$ [*M*−H]$^-$ = 1046.1815; obs. = 1046.1846. **IR** (CH_2Cl_2, 1 mg mL^{-1}): ν(CO) = 2021, 1927, 1897 cm^{-1}.

3.14 [Cu(DP^{An}-PSMAt)]$^+$

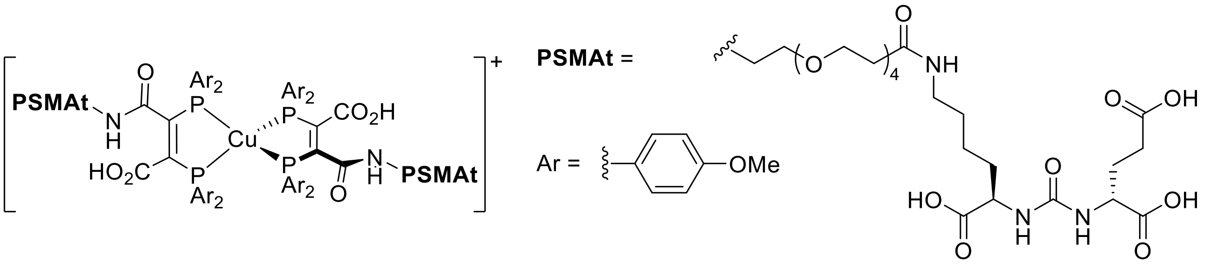

A solution of **DP^{An}-PSMAt** (~200 µg, 2 equiv.) in saline (200 µL) was added to a solution of [Cu(MeCN)$_4$][PF$_6$] (32.0 µg, 1 equiv.) in dry MeCN (32 µL), and the mixture was agitated at ambient temperature for 1 h. The crude reaction mixture was then analysed by analytical HPLC, LC-MS and HR-MS. **HPLC** (method 2): t_R = 12.75 min. **LC-MS** (ESI): 20 min method, t_R = 11.37 min; *m/z* calcd. for $C_{110}H_{141}N_8O_{38}P_4Cu$ [*M*+H]$^{2+}$ = 1184.4; obs. = 1184.5. **HR-MS** (ESI): *m/z* calcd. for $C_{110}H_{141}N_8O_{38}P_4Cu$ [*M*+H]$^{2+}$ = 1184.3791; obs. = 1184.3827.

3.15 [Cu(DP^{MEP}-PSMAt)]$^+$

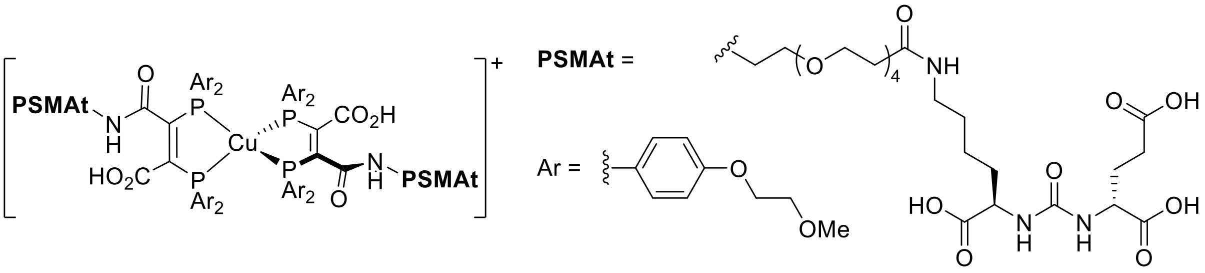

A solution of **DP^{MEP}-PSMAt** (~230 µg, 2 equiv.) in saline (200 µL) was added to a solution of [Cu(MeCN)$_4$][PF$_6$] (32.0 µg, 1 equiv.) in dry MeCN (32 µL), and the mixture was agitated at ambient temperature for 1 h. The crude reaction mixture was then analysed by analytical HPLC, LC-MS and HR-MS. **HPLC** (method 2): t_R = 12.52 min. **LC-MS** (ESI): 20 min method, t_R = 10.03 min; *m/z* calcd. for $C_{126}H_{172}N_8O_{46}P_4Cu$ [*M*+H]$^{2+}$ = 1360.5; obs. = 1360.5. **HR-MS** (ESI): *m/z* calcd. for $C_{126}H_{173}N_8O_{46}P_4Cu$ [*M*+H]$^{2+}$ = 1360.4845; obs. = 1360.4854.

4 Radiolabelling procedures and radiotracer characterisation

4.1 Kit preparation

An aqueous stock solution was prepared containing the required amounts of sodium bicarbonate, tin chloride and sodium tartrate (see Table S1). The stock solution was adjusted to pH 8–8.5 by addition of 0.1 M HCl or 0.1 M NaOH solution and then deoxygenated. Each kit was prepared by combining an aliquot of the stock solution with the required amount of diphosphine ligand (**DP^{Ph}-PSMAt**, **DP^{An}-PSMAt** or **DP^{MEP}-PSMAt** dissolved in deoxygenated 25% EtOH in H_2O) to form solutions containing the kit amounts outlined in Table S1. The kit solutions were immediately frozen and lyophilised using a freeze dryer. The lyophilised kits were stored in a freezer prior to use.

Table S1 – ^{99m}Tc kit compositions for DP-PSMAt ligands.

Components	DP^{Ph}-PSMAt		DP^{An}-PSMAt		DP^{MEP}-PSMAt	
	µmol	mg	µmol	mg	µmol	mg
**Ligand**	0.109	0.113	0.109	0.124	0.109	0.145
Tin(II) chloride dihydrate	0.110	0.025	0.110	0.025	0.110	0.025
Sodium tartrate dihydrate	1.15	0.264	1.15	0.264	1.15	0.264
Sodium bicarbonate	10.7	0.900	10.7	0.900	10.7	0.900

4.2 Radiolabelling with ^{99m}Tc

Diphosphine kit (containing **DP^{Ph}-PSMAt**, **DP^{An}-PSMAt** or **DP^{MEP}-PSMAt**) was radiolabelled with generator-produced $^{99m}TcO_4^-$ (200 MBq) in saline solution (500 µL, 0.9% NaCl in water, w/v), using the lyophilised kit described in Section 4.1. The radiolabelling reaction mixture was heated at 100 °C for 5–15 min. Aliquots were analysed by iTLC and analytical C$_{18}$-HPLC to determine the radiochemical yield.

[$^{99m}TcO_2$(DP^{Ph}-PSMAt)$_2$]$^+$: HPLC method 2, t_R = 11.8–13.1 min. RCY 76.9 ±1.9% (n = 4).

[$^{99m}TcO_2$(DP^{An}-PSMAt)$_2$]$^+$: HPLC method 2, t_R = 12.4–13.9 min. RCY 95.1 ±0.7% (n = 6).

[$^{99m}TcO_2$(DP^{MEP}-PSMAt)$_2$]$^+$: HPLC method 2, t_R = 12.0–13.4 min. RCY 95.5 ±1.3% (n = 7).

For comparative studies of **DP^{An}-PSMAt** and **DP^{MEP}-PSMAt** with **DP^{Ph}-PSMAt**, kit solutions of each [$^{99m}TcO_2$(DP-PSMAt)$_2$]$^+$ radiotracer were purified to >95% RCP by size-exclusion HPLC (Method 3):

[$^{99m}TcO_2$(DP^{Ph}-PSMAt)$_2$]$^+$: t_R = 9.7–10.9 min; **DP^{Ph}-PSMAt**: t_R = 15.1–16.5 min.

[$^{99m}TcO_2$(DP^{An}-PSMAt)$_2$]$^+$: t_R = 10.0–11.5 min; **DP^{An}-PSMAt**: t_R = 19.0–20.3 min.

[$^{99m}TcO_2(DP^{MEP}$-PSMAt$)_2]^+$: t_R = 9.7–11.1 min; **DP^{MEP}-PSMAt**: t_R = 15.6–16.7 min.

4.3 Log$_{7.4}D$ – ^{99m}Tc

The following procedure was performed in triplicate or greater repeats. A solution of either [$^{99m}TcO_2(DP^{An}$-PSMAt$)_2]^+$ or [$^{99m}TcO_2(DP^{MEP}$-PSMAt$)_2]^+$ from the radiolabelling reaction was diluted with PBS (pH 7.4) to 1 MBq in 200 µL. An aliquot (0.1 MBq, 20 µL) of either [$^{99m}TcO_2(DP^{An}$-PSMAt$)_2]^+$ or [$^{99m}TcO_2(DP^{MEP}$-PSMAt$)_2]^+$ was combined with PBS (pH 7.4, 480 µL) and octanol (500 µL) and the mixture was agitated for 30 min. The mixture was then centrifuged (10,000 rpm, 10 min) and aliquots of both the PBS and octanol layers were analysed for radioactivity using a gamma counter.

[$^{99m}TcO_2(DP^{An}$-PSMAt$)_2]^+$ log$_{7.4}D$ = −3.22 ±0.04

[$^{99m}TcO_2(DP^{MEP}$-PSMAt$)_2]^+$ log$_{7.4}D$ = −3.35 ±0.12

4.4 Radiolabelling with ^{188}Re

$^{188}ReO_4^-$ eluted from an OncoBeta ^{188}W/^{188}Re generator and concentrated as previously described.[11]

Aqueous saline solution containing $^{188}ReO_4^-$ (125 µL, 108–341 MBq) was added to an aqueous solution of sodium citrate (1 M, 50 µL) and stannous chloride (1.88 mg), and heated at 90 °C for 30 min. An aliquot of the ^{188}Re-citrate solution (85 µL, 49–130 MBq) was then immediately added to the contents of two ^{99m}Tc kits containing either DP^{An}-PSMAt or DP^{MEP}-PSMAt (double that described in Table S1), which was then heated at 90 °C for 30 min. The reaction was allowed to cool, and then PBS (420 µL) was added, the mixture vortexed and centrifuged (6 min, 13000 rpm). The supernatant was isolated and typically contained ~90% of the radioactivity. Aliquots of the supernatent were then analysed by iTLC and reverse-phase C_{18} radio-HPLC (method 2).

[$^{188}ReO_2(DP^{An}$-PSMAt$)_2]^+$: HPLC method 2, t_R = 11.7–12.1 min. RCY 84.7 ±7.1% (n = 4).

[$^{188}ReO_2(DP^{MEP}$-PSMAt$)_2]^+$: HPLC method 2, t_R = 11.9–12.2 min. RCY 83.1 ±6.8% (n = 4).

Purification: Crude reaction mixture containing either [$^{188}ReO_2(DP^{An}$-PSMAt$)_2]^+$ or [$^{188}ReO_2(DP^{MEP}$-PSMAt$)_2]^+$, was applied to a reverse-phase C_{18} analytical HPLC column and isolated using HPLC method 5. Fractions containing either [$^{188}ReO_2(DP^{An}$-PSMAt$)_2]^+$ (eluted at 41.4–43.4 min as a double peak) or [$^{188}ReO_2(DP^{MEP}$-PSMAt$)_2]^+$ (eluted at 41.0–42.5 min as a single peak) were either immediately frozen and lyophilized or neutralised upon elution with 0.125 M ammonium bicarbonate buffer (100 µL per mL eluant) and dried under a stream of N_2. The resulting samples of [$^{188}ReO_2(DP^{An}$-PSMAt$)_2]^+$ or [$^{188}ReO_2(DP^{MEP}$-PSMAt$)_2]^+$ were dissolved in PBS and measured >95% radiochemical purity (by analytical C_{18} radio-HPLC and iTLC).

4.5 $\mathrm{Log}_{7.4}D$ – ^{188}Re

The following procedure was performed in triplicate or greater repeats. A solution of purified [^{188}ReO$_2$(DP^{An}-PSMAt)$_2$]$^+$ or [^{188}ReO$_2$(DP^{MEP}-PSMAt)$_2$]$^+$ was diluted with PBS (pH 7.4) to 1 MBq in 200 µL. An aliquot (0.1 MBq, 20 µL) of either [^{188}ReO$_2$(DP^{An}-PSMAt)$_2$]$^+$ or [^{188}ReO$_2$(DP^{MEP}-PSMAt)$_2$]$^+$ was combined with PBS (pH 7.4, 480 µL) and octanol (500 µL) and the mixture was agitated for 30 min. The mixture was then centrifuged (10,000 rpm, 10 min) and aliquots of both the PBS and octanol layers were analysed for radioactivity using a gamma counter.

[^{188}ReO$_2$(DP^{An}-PSMAt)$_2$]$^+$ $\log_{7.4}D = -3.47 \pm 0.05$

[^{188}ReO$_2$(DP^{MEP}-PSMAt)$_2$]$^+$ $\log_{7.4}D = -3.91 \pm 0.10$

4.6 Preparation of ^{99g}Tc complexes using $^{99g}TcO_4^-$

Diphosphine kit (containing **DP^{An}-PSMAt** or **DP^{MEP}-PSMAt**) was radiolabelled with generator-produced $^{99m}TcO_4^-$ (5 MBq) in saline solution (500 µL, 0.9% NaCl in water, w/v) and spiked with [NH$_4$][^{99g}TcO$_4$] (0.06 kBq), using the lyophilised kit described in Section 4.1. The radiolabelling reaction mixture was heated at 100 °C for 10 min. The product was isolated by analytical HPLC (Method 4) with [$^{99m/99g}$TcO$_2$(DP^{An}-PSMAt)$_2$]$^+$ eluting at t_R = 16.4–17.4 min and [$^{99m/99g}$TcO$_2$(DP^{MEP}-PSMAt)$_2$]$^+$ eluting at t_R = 16.1–17.2 min. Purified fractions were then concentrated by lyophilisation and analysed by LC-MS (40 min method).

[$^{99m/99g}$TcO$_2$(DP^{An}-PSMAt)$_2$]$^+$ **LC-MS** t_R = 18.1–18.4 min; **MS** (ESI): *m/z* calcd. for $C_{110}H_{141}N_8O_{40}P_4{}^{99g}Tc$ $[M+H]^{2+}$ = 1218.9; obs. = 1218.8 and *m/z* calcd. for $C_{112}H_{145}N_8O_{44}P_4{}^{99g}Tc$ $[M+H+(HCO_2H)_2]^{2+}$ = 1264.9; obs. = 1265.3.

[$^{99m/99g}$TcO$_2$(DP^{MEP}-PSMAt)$_2$]$^+$ **LC-MS** t_R = 17.7–18.0 min; **MS** (ESI): *m/z* calcd. for $C_{126}H_{173}N_8O_{48}P_4{}^{99g}Tc$ $[M+H]^{2+}$ = 1395.0; obs. = 1394.9 and *m/z* calcd. for $C_{128}H_{177}N_8O_{52}P_4{}^{99g}Tc$ $[M+H+(HCO_2H)_2]^{2+}$ = 1441.0; obs. = 1441.3.

4.7 Radiolabelling with ^{64}Cu

The ^{64}Cu radiolabelling procedure was adapted from that previously described for DP^{Ph}-PSMAt.[3]

The $^{64}Cu^{2+}$ solutions (in 0.1 M HCl) were dried under a flow of N_2 with heating at 100 °C, and the residue was re-dissolved in ammonium acetate solution (0.1 M, pH 7). An aliquot of the $^{64}Cu^{2+}$ solution (7–8 MBq, 84 µL) was added to a solution of **DP^{X}-PSMAt** (43.3 µmol; 50 µg when X = An; 57.5 µg when X = MEP) in ammonium acetate (0.1 M, pH 7, 50 µL). The radiolabelling mixture was left to react at ambient temperature (~22 °C) for 20 min. Aliquots were analysed by iTLC and analytical radio-HPLC to determine the RCYs. By radio-HPLC, unreacted $^{64}Cu^{2+}$ would be expected to elute with the solvent

front at 2.0–3.5 min. iTLC analysis was also undertaken to enable the quantification of unreacted $^{64}Cu^{2+}$ and $[^{64}Cu(DP\text{-}PSMAt)_2]^+$, using citrate buffer (0.1 M, pH 5) as the mobile phase. R_f values: unreacted $^{64}Cu^{2+}$ > 0.9, and $[^{64}Cu(DP\text{-}PSMAt)_2]^+$ < 0.1.

$[^{64}Cu(DP^{An}\text{-}PSMAt)_2]^+$: HPLC method 2, t_R = 12.78 min. RCY 79% by radio-HPLC. Unreacted $^{64}Cu^{2+}$ was not observed by iTLC or HPLC.

$[^{64}Cu(DP^{MEP}\text{-}PSMAt)_2]^+$: HPLC method 2, t_R = 12.67 min. RCY 87% by radio-HPLC. Unreacted $^{64}Cu^{2+}$ was not observed by iTLC or HPLC.

Purification: Crude reaction mixture containing either $[^{64}Cu(DP^{An}\text{-}PSMAt)_2]^+$ or $[^{64}Cu(DP^{MEP}\text{-}PSMAt)_2]^+$, was applied to a reverse-phase C_{18} analytical HPLC column and isolated using HPLC method 2. Fractions containing either $[^{64}Cu(DP^{An}\text{-}PSMAt)_2]^+$ (eluted at 12.6–13.0 min) or $[^{64}Cu(DP^{MEP}\text{-}PSMAt)_2]^+$ (eluted at 12.5–12.9 min) were neutralised upon elution with 0.125 M ammonium bicarbonate buffer (100 µL per mL eluant) and dried under a stream of N_2. The resulting samples of $[^{64}Cu(DP^{An}\text{-}PSMAt)_2]^+$ or $[^{64}Cu(DP^{MEP}\text{-}PSMAt)_2]^+$ were dissolved in PBS and measured >98.5% radiochemical purity (by analytical C_{18} radio-HPLC).

4.8 Stability studies

4.8.1 Kit stability with ^{99m}Tc and ^{188}Re

Radiotracers $[MO_2(DP^{An}\text{-}PSMAt)_2]^+$ or $[MO_2(DP^{MEP}\text{-}PSMAt)_2]^+$ (M = ^{99m}Tc, ^{188}Re) were synthesised as described previously (see Sections 4.2 and 4.4) and left at ambient temperature (~25 °C). At timepoints (0 h, 4 h and 24 h for ^{99m}Tc; 0 h and 24 h for ^{188}Re), the kits were vortexed and aliquots were analysed by analytical C_{18} radio-HPLC (Method 2) and iTLC. Results are shown in Figure S2 andFigure S3.

4.8.2 PBS stability with ^{99m}Tc, ^{188}Re and ^{64}Cu

^{99m}Tc: Three solutions of either $[^{99m}TcO_2(DP^{An}\text{-}PSMAt)_2]^+$ or $[^{99m}TcO_2(DP^{MEP}\text{-}PSMAt)_2]^+$ (unpurified kit solution, >95% RCP, 7–9 MBq in 25 µL) were each added to PBS (475 µL) and incubated at 37 °C. A sample was removed at 1 h, 4 h and 24 h timepoints and analysed by analytical C_{18} radio-HPLC (Method 2). Results are shown in Figure S4.

^{188}Re: A solution of either $[^{188}ReO_2(DP^{An}\text{-}PSMAt)_2]^+$ or $[^{188}ReO_2(DP^{MEP}\text{-}PSMAt)_2]^+$ (purified, >98% RCP, 3.9–5.5 MBq in 25–35 µL PBS) was diluted with PBS (350 µL total volume) and incubated at 37 °C. Aliquots were taken at 4 h and 24 h timepoints and analysed by analytical C_{18} radio-HPLC (Method 2). Results are shown in Figure S5.

^{64}Cu: A solution of either [^{64}Cu(DP^{An}-PSMAt)$_2$]$^+$ or [^{64}Cu(DP^{MEP}-PSMAt)$_2$]$^+$ (purified, >98% RCP, 0.6–0.8 MBq in 8–20 µL PBS) was diluted with PBS (350 µL total volume) and incubated at 37 °C. After 24 h, the sample was analysed by analytical C$_{18}$ radio-HPLC (Method 2). Results are shown in Figure S6.

4.8.3 Serum stability with ^{99m}Tc, ^{188}Re and ^{64}Cu

^{99m}Tc: A solution of either [^{99m}TcO$_2$(DP^{An}-PSMAt)$_2$]$^+$ or [^{99m}TcO$_2$(DP^{MEP}-PSMAt)$_2$]$^+$ (unpurified kit solution, >95% RCP, 7 MBq in 21–23 µL) was added to filtered human serum (900 µL, Sigma-Aldrich) and incubated at 37 °C. Aliquots were taken at 1 h (≈0.7 MBq), 4 h (≈0.7 MBq) and 24 h (≈0.35 MBq). Each aliquot was treated with an equal amount of ice-cold MeCN to precipitate serum proteins and centrifuged (10,000 rpm, 10 min). The supernatant was isolated, MeCN evaporated under a stream of $N_{2,}$ and the resultant solution analysed by analytical C$_{18}$ radio-HPLC (Method 2). Results are shown in Figure S7.

^{188}Re: Three solutions of either [^{188}ReO$_2$(DP^{An}-PSMAt)$_2$]$^+$ or [^{188}ReO$_2$(DP^{MEP}-PSMAt)$_2$]$^+$ (purified, >98% RCP, 3.9–5.9 MBq in 15–35 µL PBS) were added to filtered human serum (350 µL total volume, Sigma-Aldrich) and incubated at 37 °C. Samples were removed at 1 h, 4 h and 24 h timepoints and treated with ice-cold MeCN (350 µL) to precipitate serum proteins and centrifuged (10,000 rpm, 10 min). The supernatant was isolated, MeCN evaporated under a stream of $N_{2,}$ and the resultant solution analysed by analytical C$_{18}$ radio-HPLC (Method 2). Results are shown in Figure S8.

^{64}Cu: A solution of either purified [^{64}Cu(DP^{An}-PSMAt)$_2$]$^+$ (2.3 MBq in 80 µL PBS) or [^{64}Cu(DP^{MEP}-PSMAt)$_2$]$^+$ (4.0 MBq in 100 µL PBS) was added to filtered human serum (900 and 1000 µL, respectively; Sigma-Aldrich) and incubated at 37 °C. Aliquots were taken at 1 h, 4 h and 24 h (≈0.40 MBq for [^{64}Cu(DP^{An}-PSMAt)$_2$]$^+$ and ≈0.65 MBq for [^{64}Cu(DP^{MEP}-PSMAt)$_2$]$^+$ removed at each timepoint). Each aliquot was treated with an equal amount of ice-cold MeCN to precipitate serum proteins and centrifuged (10,000 rpm, 10 min). The supernatant was isolated, MeCN evaporated under a stream of $N_{2,}$ and the resultant solution analysed by analytical C$_{18}$ radio-HPLC (Method 2). Results are shown in Figure S9.

4.9 ^{99m}Tc uptake in DU145, DU145-PSMA+ and LNCaP cells

The following experiment was performed in biological triplicate.

A panel of cell lines were selected that either expressed GCP(II)/PSMA (DU145-PSMA+ (genetically modified to express PSMA),[12] and LNCaP (CRL-1740)), or had low GCP(II)/PSMA expression (DU145 (HTB-81)). All cell lines were cultured in RPMI 1640 medium (R0883, Sigma) containing 10% foetal bovine serum, 2 mM L-glutamine, and 100 U mL^{-1} penicillin and 100 µg mL^{-1} streptomycin. Cells were maintained at 37 °C and 5% CO$_2$. Cells were seeded in 6-well plates at a density of 5 × 10^5 cells per well

in 2 mL complete media to achieve 70–80% confluency the following day. Prior to treating cells, cell medium (1 mL/well) was replaced. Solutions containing either $[^{99m}TcO_2(DP^{Ph}\text{-}PSMAt)_2]^+$, $[^{99m}TcO_2(DP^{An}\text{-}PSMAt)_2]^+$ or $[^{99m}TcO_2(DP^{MEP}\text{-}PSMAt)_2]^+$ (50 kBq in 10 µL of PBS, >95% radiochemical purity) were added to each well, and the cells incubated at 37 °C for 1 h. Uptake studies were also performed after a 2 min incubation with the PSMA inhibitor 2-(phosphonomethyl)pentane-1,5-dioic acid (PMPA; 30 µL of 750 µM PMPA solution/well). After 60 min incubation, the supernatant was removed, and the cells were washed with PBS solution (2 × 1 mL). The cells were lysed with ice cold radioimmunoprecipitation assay buffer (RIPA buffer, 500 µL; 150 mM sodium chloride, 0.1% w/w sodium dodecyl sulfate (SDS), 0.5% w/w sodium deoxycholate (NaDOC), 1% w/w Triton-X) and samples were collected for radioactivity counting. Results are depicted as means ± SD of independent biological experiments (performed on different days with different radiotracer preparations).

4.10 *In vivo* imaging and biodistribution (DU145-PSMA+ and LNCaP)

Preparation of tumour-bearing mice: All animal experiments were ethically reviewed by an Animal Welfare & Ethical Review Board at either King's College London or Bart's Cancer Institute and carried out in accordance with the Animals (Scientific Procedures) Act 1986 UK Home Office regulations governing animal experimentation. Subcutaneous prostate cancer xenografts were produced in SCID/beige mice (male, 7–12 weeks old) by injecting 4×10^6 DU145-PSMA+ or DU145 cells suspended in PBS (100 µL) on the right shoulder. Imaging and biodistribution studies were performed once a tumour had reached 5–10 mm in diameter (3–4 weeks after injection). Subcutaneous LNCaP prostate cancer xenografts were produced in athymic nude (Crl:NU(NCr)-*Foxn1^{nu}*) mice (male, 6–7 weeks old) by injecting 5×10^6 LNCaP cells suspended in PBS:matrigel (1:1, 200 µL) on the right flank. Biodistribution studies using ^{99m}Tc radiotracers were performed once a tumour had reached 150–300 mm^3 in volume (2–7 weeks after injection).

SCID/beige mice bearing DU145-PSMA+ prostate cancer tumour xenografts

SPECT/CT scanning and biodistribution studies: The ^{99m}Tc radiotracers (3.9–4.5 MBq, 3 mice per group, containing DP^{An}-PSMAt = 3.0 µg or DP^{MEP}-PSMAt = 3.5 µg) were administered via tail vein injection under isoflurane anaesthesia. Following this, SPECT/CT scanning was carried out in one of two methods outlined below, to maintain 2 h biodistribution timepoints:

i. The mouse was positioned on a single heated bed in a nanoScan SPECT/CT 80W scanner (Mediso Ltd., Budapest, Hungary) calibrated for ^{99m}Tc. A helical CT scan was acquired (50 kV X-ray source, 170 ms exposure time in 360 projections over 3 min). At 15 min post-injection, whole body SPECT scans were acquired (1 × 15 min, 3 × 30 min, conducted sequentially) with a frame time of 13 s and 26 s (using a 4-head scanner with 4 × 9 [1.4 mm] pinhole collimators

in helical scanning mode). At 2 h post-injection, the animals were euthanized by cervical dislocation, organs/tissues harvested and weighed, and radioactivity counted using a gamma counter.

ii. The mouse remained under isoflurane anaesthesia on a heated bed, followed by euthanasia by pentobarbital injection at 2 h post-injection. The mouse was then scanned using a nanoScan SPECT/CT 80W scanner (Mediso Ltd., Budapest, Hungary) calibrated for ^{99m}Tc. A helical CT scan was acquired (50 kV X-ray source, 170 ms exposure time in 360 projections over 3 min). Subsequently, a 30 min whole-body SPECT scan was acquired with a frame time of 45 s (using a 4-head scanner with 4 × 9 [1.4 mm] pinhole collimators in helical scanning mode). After scanning, the organs/tissues were harvested, weighed and radioactivity counted using a gamma counter.

Urine was collected and directly analysed by reverse-phase radio-HPLC (method 2). Data were analysed in GraphPad Prism 9 (version 9.1.1) and expressed as mean ± standard deviation (SD). Student *t* tests were used to determine statistical significance.

SPECT/CT images were reconstructed in a 256 × 256 matrix using HiSPECT (ScivisGmbH) (a reconstruction software package) and visualized and quantified using VivoQuant v.3.5 software (InVicro LLC., Boston, USA).

Athymic nude mice bearing LNCaP prostate cancer tumour xenografts

Biodistribution studies: The ^{99m}Tc radiotracers (4.0–4.9 MBq, containing DP^{An}-PSMAt = 3.0 µg or DP^{MEP}-PSMAt = 3.5 µg) were administered via tail vein injection. The animals were euthanized by cervical dislocation 2 h post-injection, organs/tissues harvested and weighed, and radioactivity counted using a gamma counter. For [^{99m}TcO$_2$(DP^{An}-PSMAt)$_2$]$^+$, four mice per group were utilised, and for [^{99m}TcO$_2$(DP^{MEP}-PSMAt)$_2$]$^+$, three mice per group were utilised. Data were analysed in GraphPad Prism 9 (ver 9.1.1) and expressed as mean ± standard deviation (SD). Student *t* tests were used to determine statistical significance.

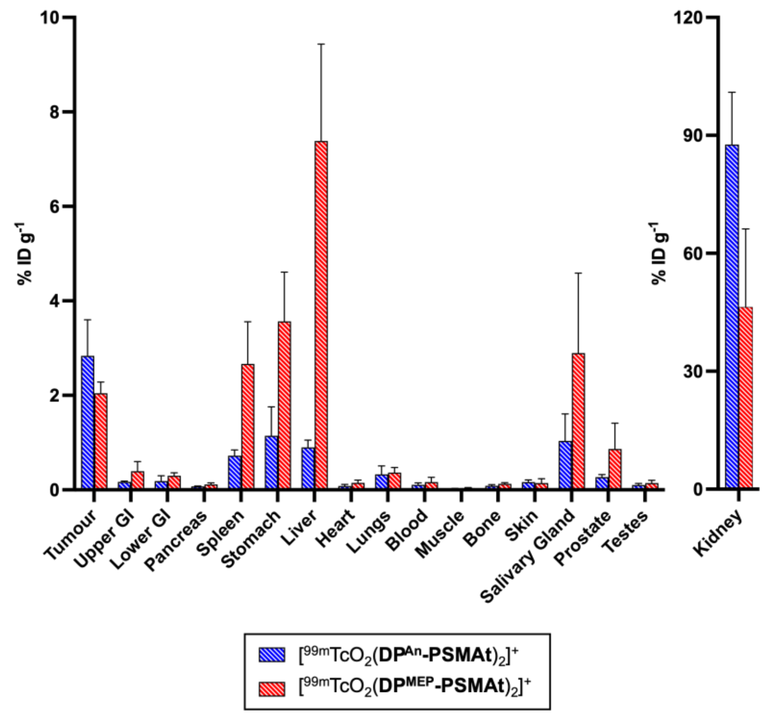

*Figure S1 – Biodistribution (2 h post-injection) of [$^{99m}TcO_2$(**DP^{An}-PSMAt**)$_2$]$^+$ or [$^{99m}TcO_2$(**DP^{MEP}-PSMAt**)$_2$]$^+$ in nude mice bearing LNCaP xenografts (mean ± SD, n = 3–4).*

5 Stability studies – HPLC chromatograms and analysis

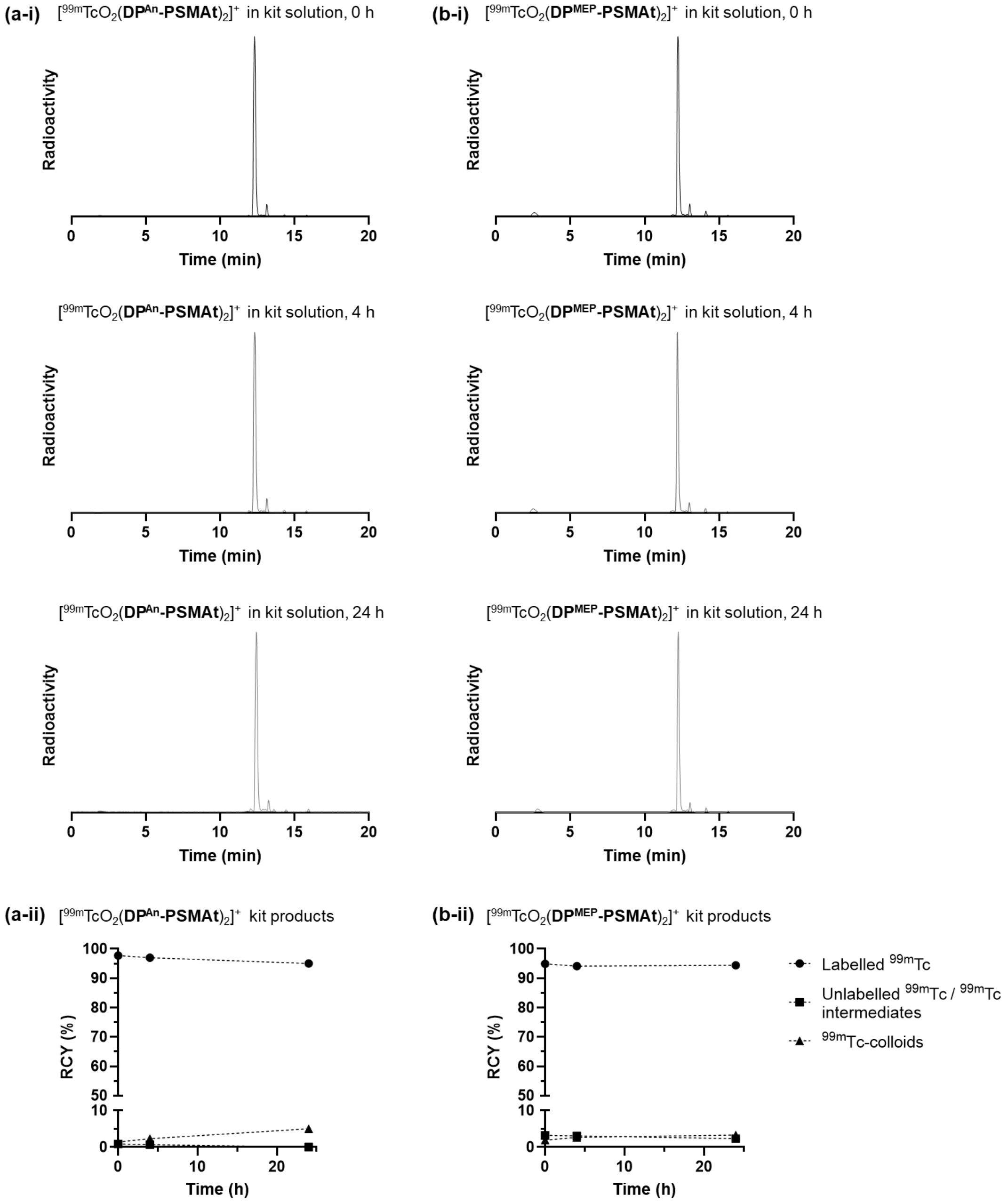

*Figure S2 – HPLC radiochromatograms of (a-i) [$^{99m}TcO_2$(**DP^{An}-PSMAt**)$_2$]$^+$ and (b-i) [$^{99m}TcO_2$(**DP^{MEP}-PSMAt**)$_2$]$^+$ radiolabelling kit solutions at 0 h, 4 h and 24 h. RCYs of radiolabelling products of (a-ii) [$^{99m}TcO_2$(**DP^{An}-PSMAt**)$_2$]$^+$ and (b-ii) [$^{99m}TcO_2$(**DP^{MEP}-PSMAt**)$_2$]$^+$ from radio-HPLC and radio-iTLC analysis. Both radiotracer kit solutions show high stability over 24 h.*

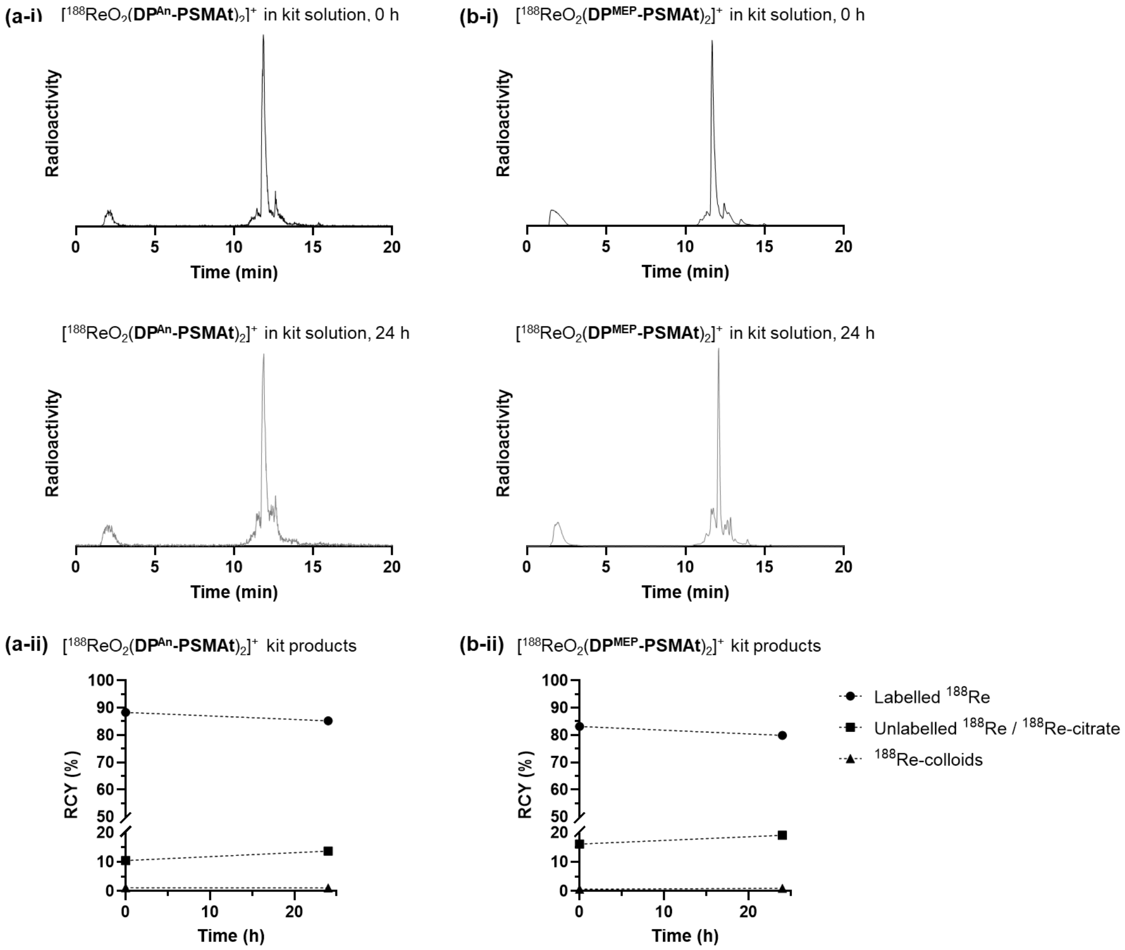

*Figure S3 – HPLC radiochromatograms of (a-i) [$^{188}ReO_2$(**DP^{An}-PSMAt**)$_2$]$^+$ and (b-i) [$^{188}ReO_2$(**DP^{MEP}-PSMAt**)$_2$]$^+$ radiolabelling kit solutions at 0 h and 24 h. RCYs of radiolabelling products of (a-ii) [$^{188}ReO_2$(**DP^{An}-PSMAt**)$_2$]$^+$ and (b-ii) [$^{188}ReO_2$(**DP^{MEP}-PSMAt**)$_2$]$^+$ from radio-HPLC and radio-iTLC analysis. Both radiotracer kit solutions show high stability over 24 h.*

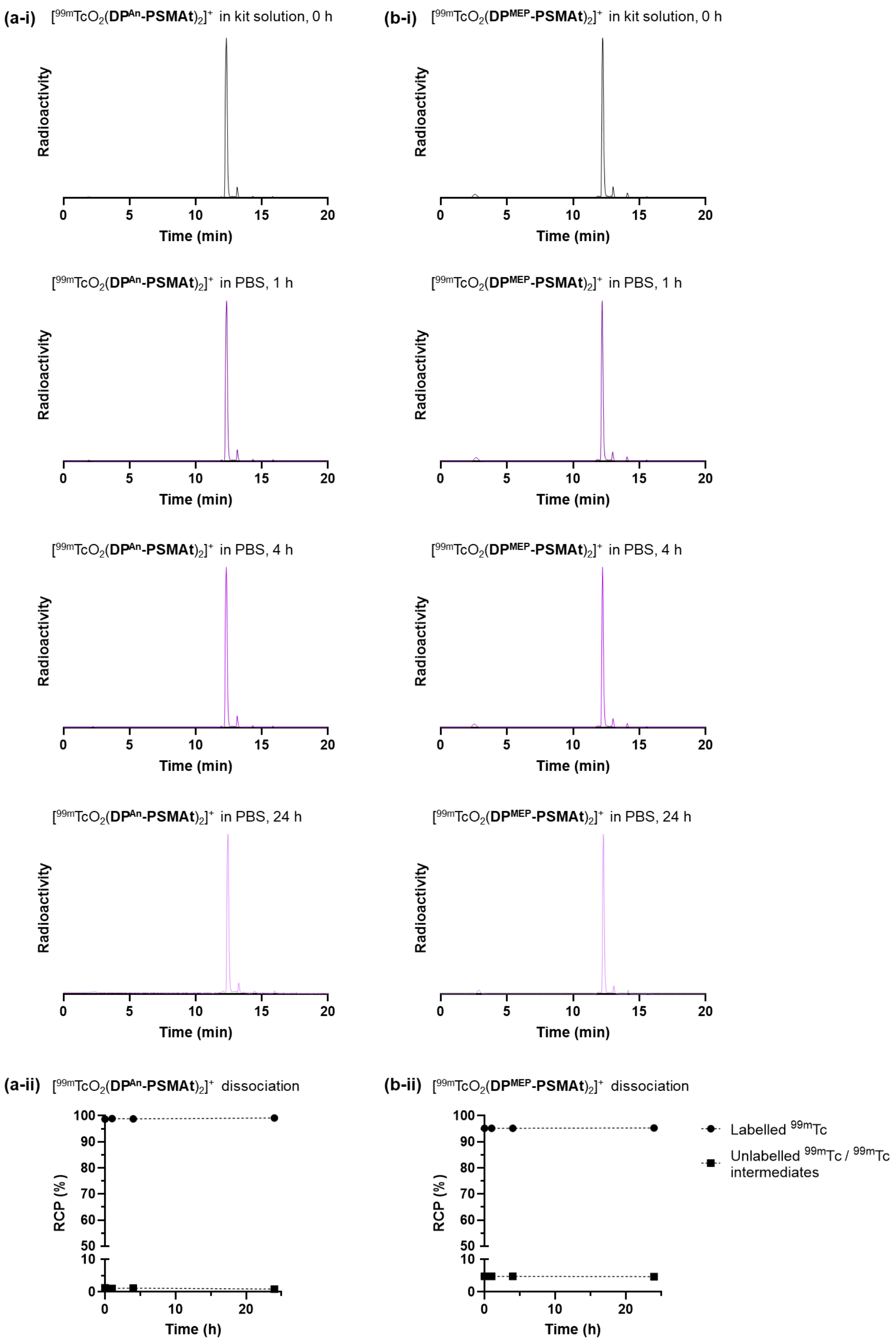

*Figure S4 – HPLC radiochromatograms of (a-i) [$^{99m}TcO_2$(**DP^{An}-PSMAt**)$_2$]$^+$ and (b-i) [$^{99m}TcO_2$(**DP^{MEP}-PSMAt**)$_2$]$^+$ incubated at 37 °C in PBS at 1 h, 4 h and 24 h. RCPs of (a-ii) [$^{99m}TcO_2$(**DP^{An}-PSMAt**)$_2$]$^+$ and (b-ii) [$^{99m}TcO_2$(**DP^{MEP}-PSMAt**)$_2$]$^+$ from radio-HPLC analysis show minimal dissociation to 'free ^{99m}Tc' over 24 h.*

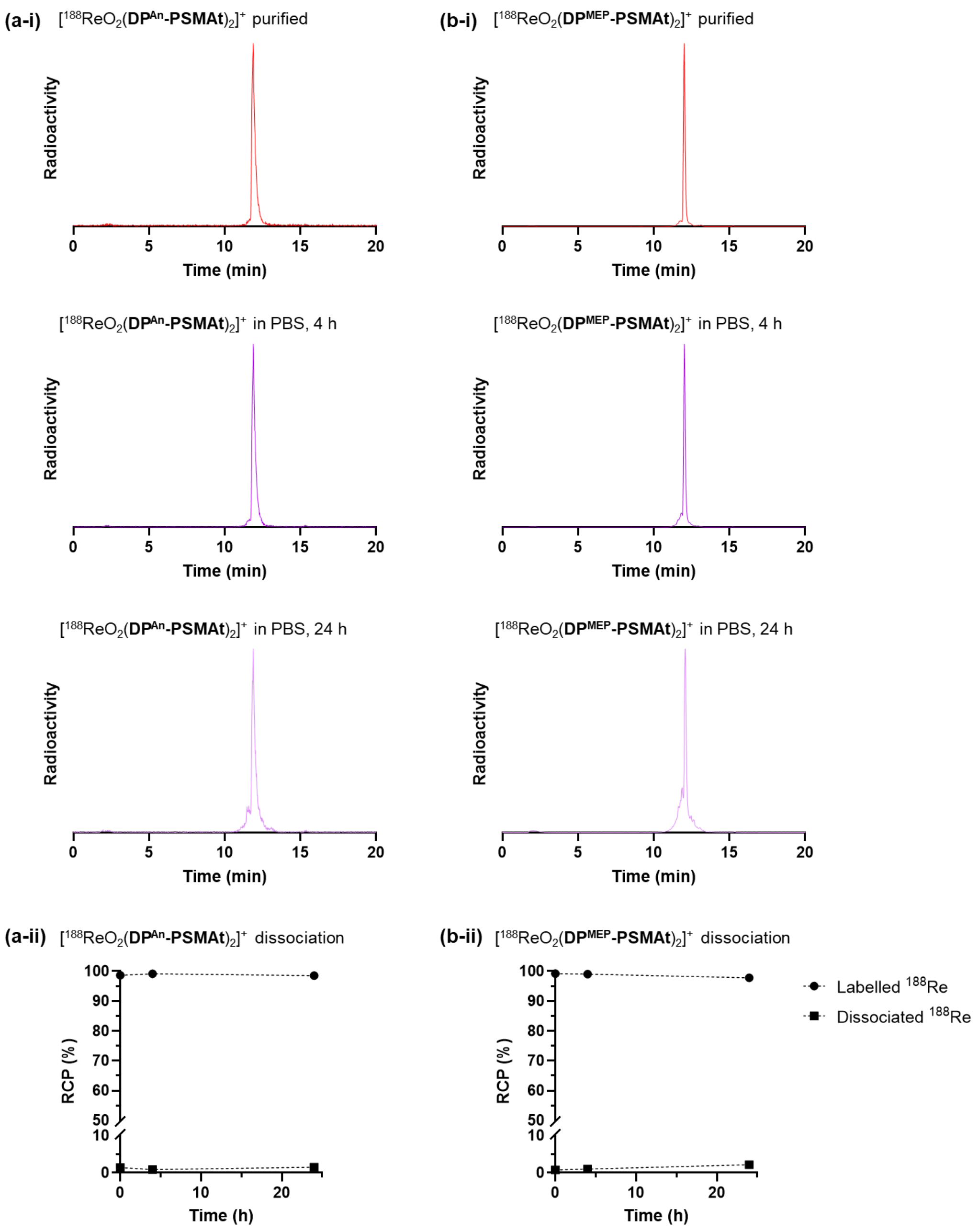

*Figure S5 – HPLC radiochromatograms of (a-i) [^{188}ReO$_2$(**DP^{An}-PSMAt**)$_2$]$^+$ and (b-i) [^{188}ReO$_2$(**DP^{MEP}-PSMAt**)$_2$]$^+$ incubated at 37 °C in PBS at 4 h and 24 h. RCPs of (a-ii) [^{188}ReO$_2$(**DP^{An}-PSMAt**)$_2$]$^+$ and (b-ii) [^{188}ReO$_2$(**DP^{MEP}-PSMAt**)$_2$]$^+$ from radio-HPLC analysis show minimal dissociation to 'free ^{188}Re' over 24 h.*

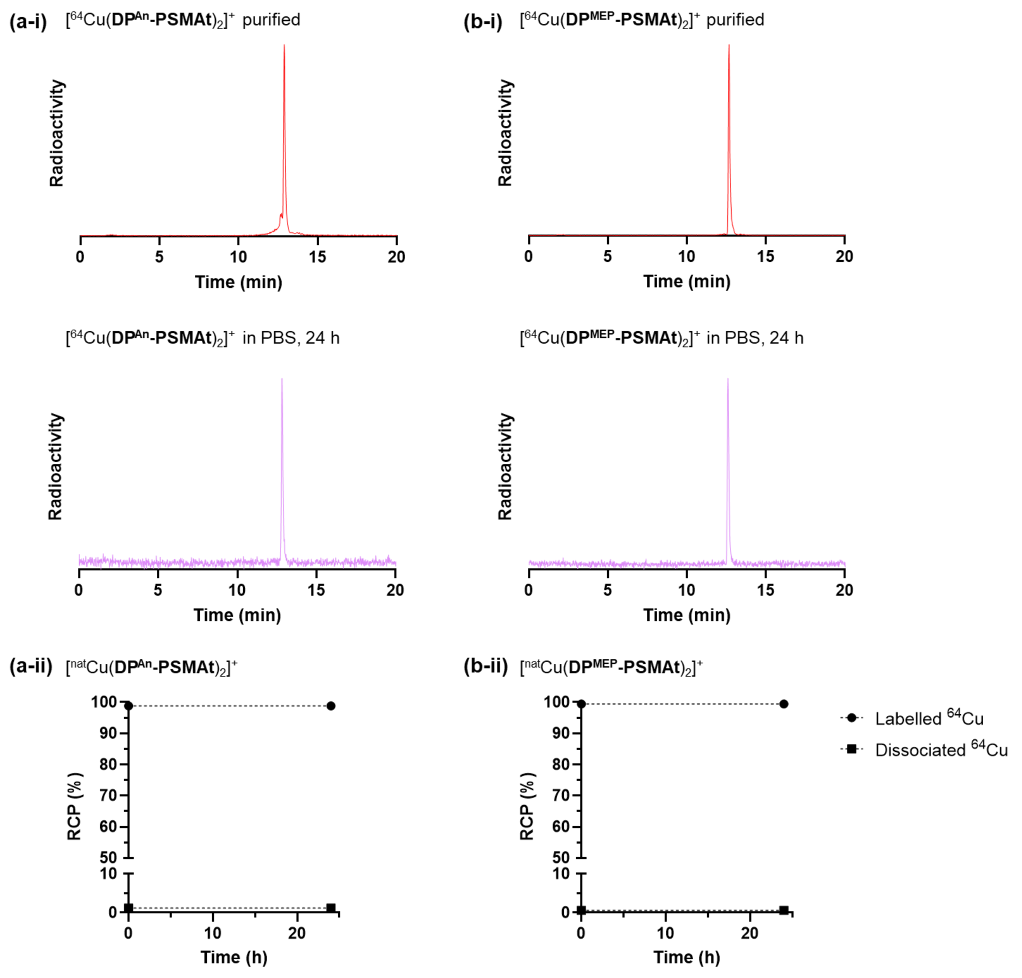

*Figure S6 – HPLC radiochromatograms of (a-i) [^{64}Cu(**DP^{An}-PSMAt**)$_2$]$^+$ and (b-i) [^{64}Cu(**DP^{MEP}-PSMAt**)$_2$]$^+$ incubated at 37 °C in PBS at 24 h. RCPs of (a-ii) [^{64}Cu(**DP^{An}-PSMAt**)$_2$]$^+$ and (b-ii) [^{64}Cu(**DP^{MEP}-PSMAt**)$_2$]$^+$ from radio-HPLC analysis show minimal dissociation to 'free ^{64}Cu' over 24 h.*

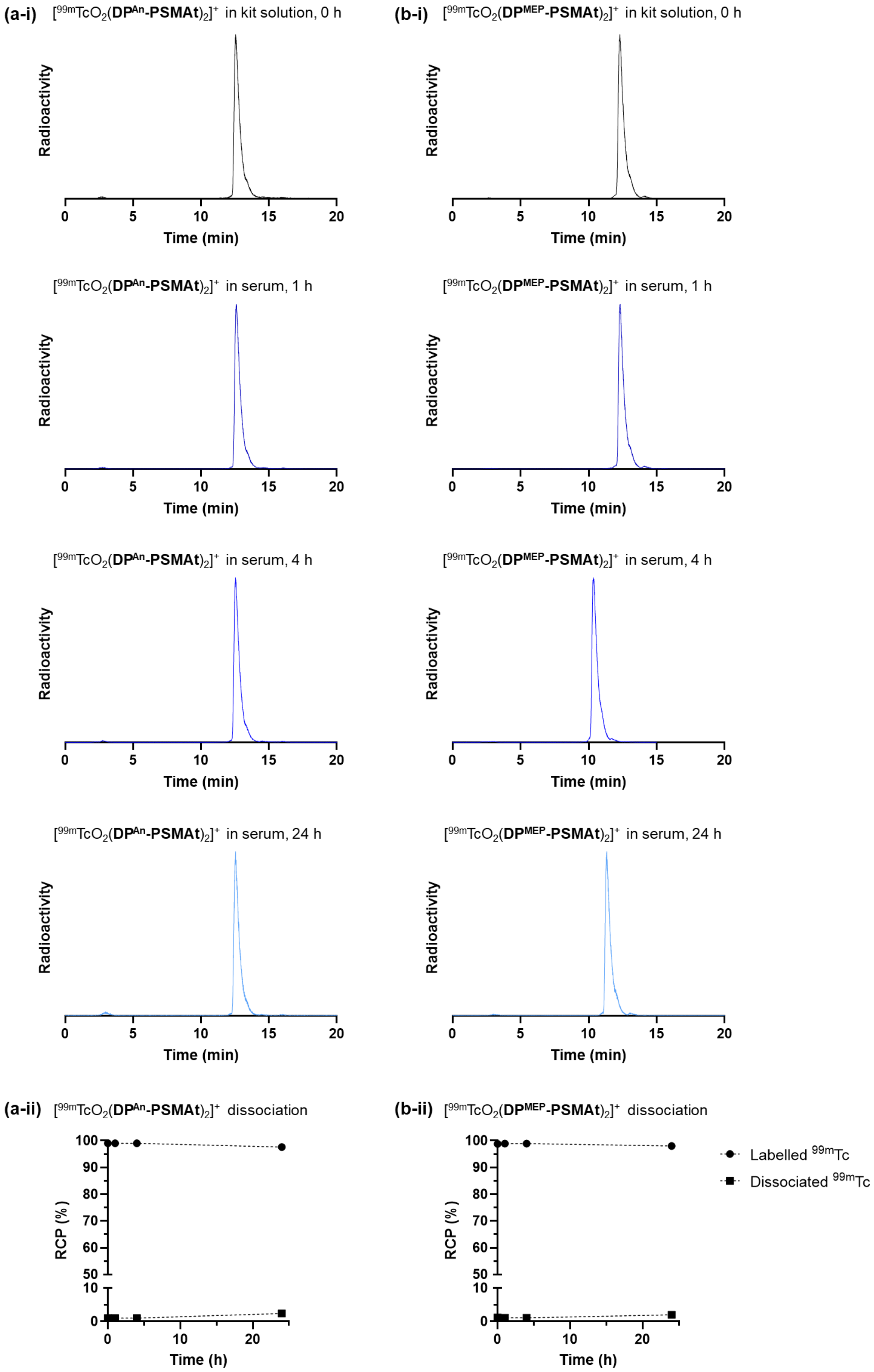

*Figure S7 – HPLC radiochromatograms of (a-i) [$^{99m}TcO_2$(**DP^{An}-PSMAt**)$_2$]$^+$ and (b-i) [$^{99m}TcO_2$(**DP^{MEP}-PSMAt**)$_2$]$^+$ incubated at 37 °C in human serum at 1 h, 4 h and 24 h. RCPs of (a-ii) [$^{99m}TcO_2$(**DP^{An}-PSMAt**)$_2$]$^+$ and (b-ii) [$^{99m}TcO_2$(**DP^{MEP}-PSMAt**)$_2$]$^+$ from radio-HPLC analysis show minimal dissociation to 'free ^{99m}Tc' over 24 h.*

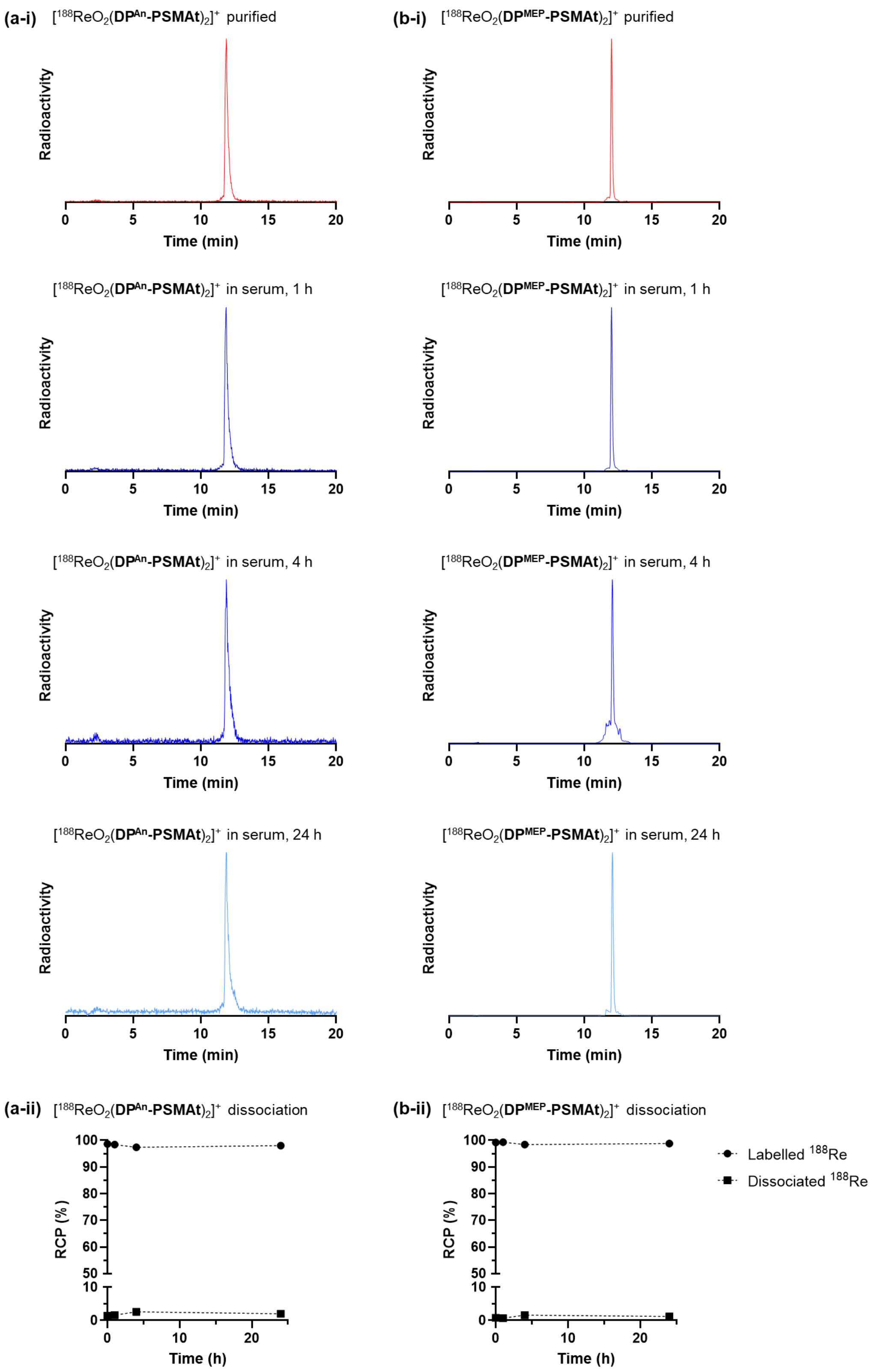

*Figure S8 – HPLC radiochromatograms of (a-i) [^{188}ReO$_2$(**DP^{An}-PSMAt**)$_2$]$^+$ and (b-i) [^{188}ReO$_2$(**DP^{MEP}-PSMAt**)$_2$]$^+$ incubated at 37 °C in human serum at 1 h, 4 h and 24 h. RCPs of (a-ii) [^{188}ReO$_2$(**DP^{An}-PSMAt**)$_2$]$^+$ and (b-ii) [^{188}ReO$_2$(**DP^{MEP}-PSMAt**)$_2$]$^+$ from radio-HPLC analysis show minimal dissociation to 'free ^{188}Re' over 24 h.*

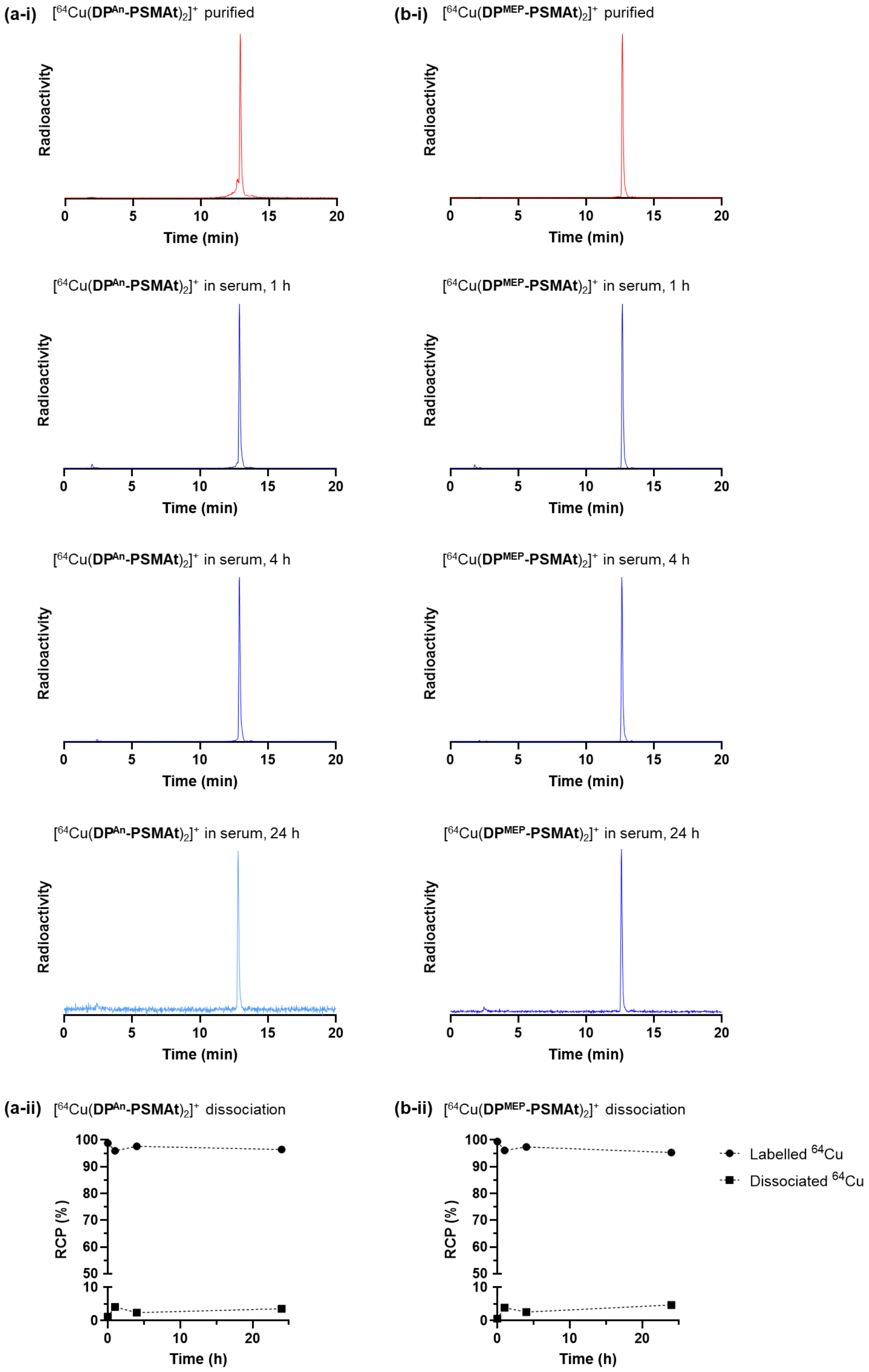

*Figure S9 – HPLC radiochromatograms of (a-i) [^{64}Cu(**DP^{An}-PSMAt**)$_2$]$^+$ and (b-i) [^{64}Cu(**DP^{MEP}-PSMAt**)$_2$]$^+$ incubated at 37 °C in human serum at 1 h, 4 24 h. RCPs of (a-ii) [^{64}Cu(**DP^{An}-PSMAt**)$_2$]$^+$ and (b-ii) [^{64}Cu(**DP^{MEP}-PSMAt**)$_2$]$^+$ from radio-HPLC analysis show minimal dissociation to 'free ^{64}Cu'.*

6 Conjugation screen – LC-MS chromatograms

The general experimental procedure is outlined in Section 3.10. Where precursor amines showed little to no UV absorption, the total ion chromatogram (TIC) or expected ion chromatogram (XIC) has been used to demonstrate their retention times. DP^{An}-OH/DP^{MEP}-OH refers to hydrolysed DP anhydrides (ring-opened with H_2O). Data were obtained using an analytical HPLC-LRMS system.

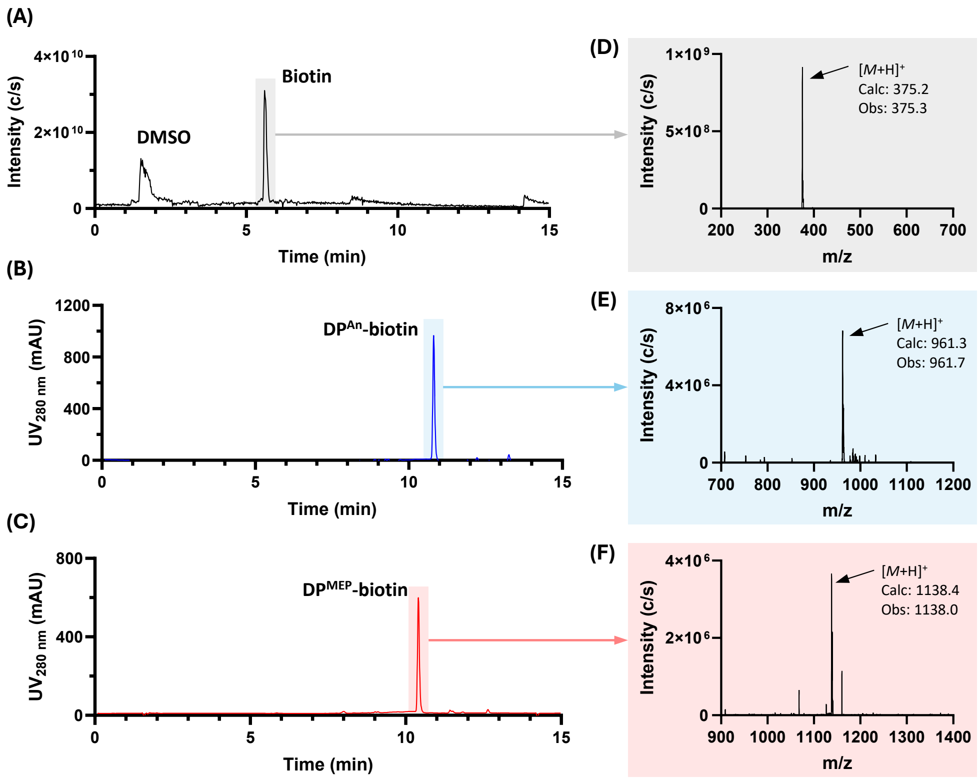

*Figure S10 – Analytical reverse-phase chromatograms showing (A) TIC of biotin-(PEG)-NH$_2$; (B) UV$_{280\,nm}$ of **DP^{An}** and biotin reaction mixture; (C) UV$_{280\,nm}$ of **DP^{MEP}** and biotin reaction mixture. MS of (D) biotin-(PEG)$_2$-NH$_2$; (E) **DP^{An}-biotin**; (F) **DP^{MEP}-biotin**.*

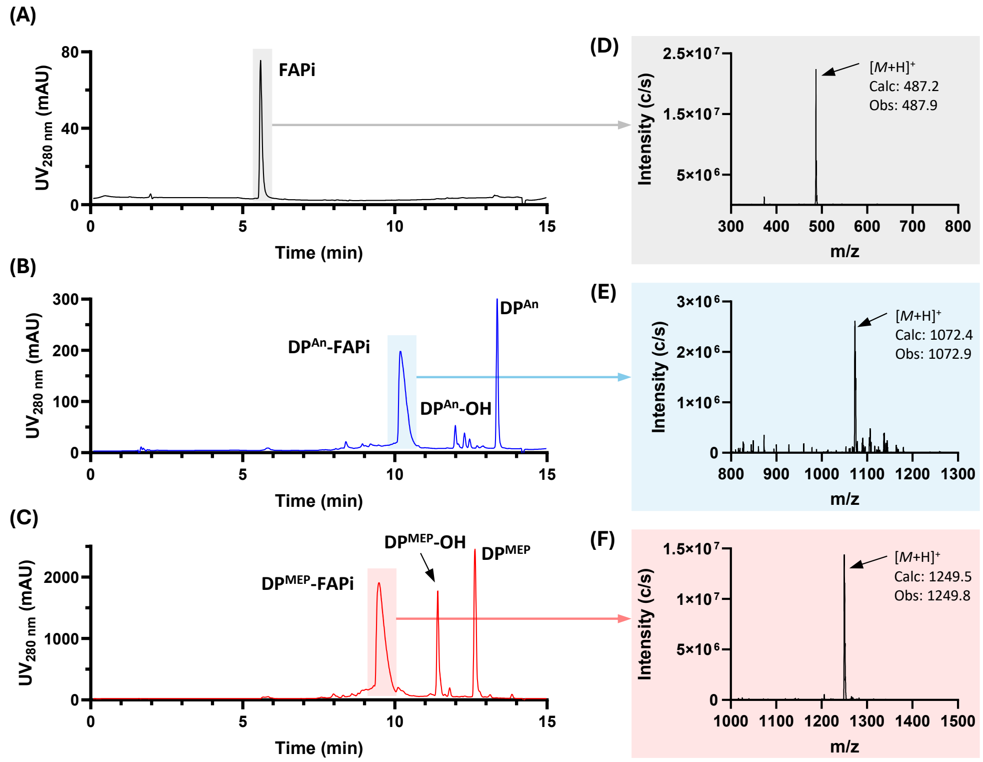

Figure S11 – Analytical reverse-phase chromatograms showing (A) UV$_{280\ nm}$ of FAPi; (B) UV$_{280\ nm}$ of ***DP^{An}*** *and FAPi reaction mixture; (C) UV$_{280\ nm}$ of* ***DP^{MEP}*** *and FAPi reaction mixture. MS of (D) FAPi; (E)* ***DP^{An}-FAPi****; (F)* ***DP^{MEP}-FAPi****.*

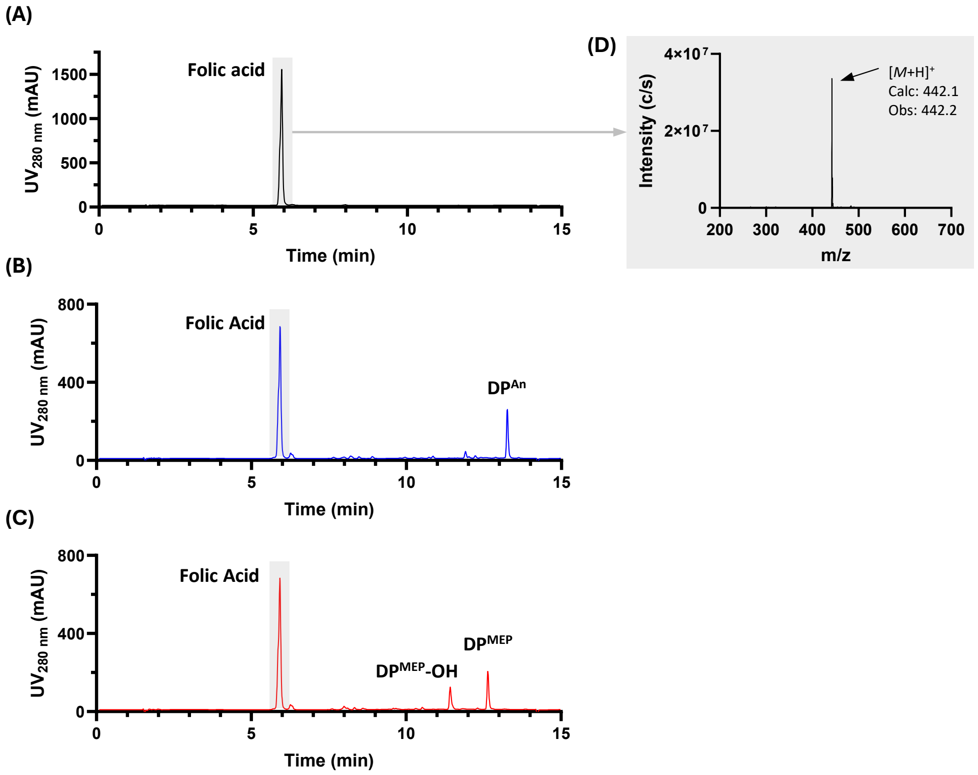

Figure S12 – Analytical reverse-phase chromatograms showing (A) UV$_{280\ nm}$ of folic acid; (B) UV$_{280\ nm}$ of ***DP^{An}*** *and folic acid reaction mixture; (C) UV$_{280\ nm}$ of* ***DP^{MEP}*** *and folic acid reaction mixture. (D) MS of folic acid.*

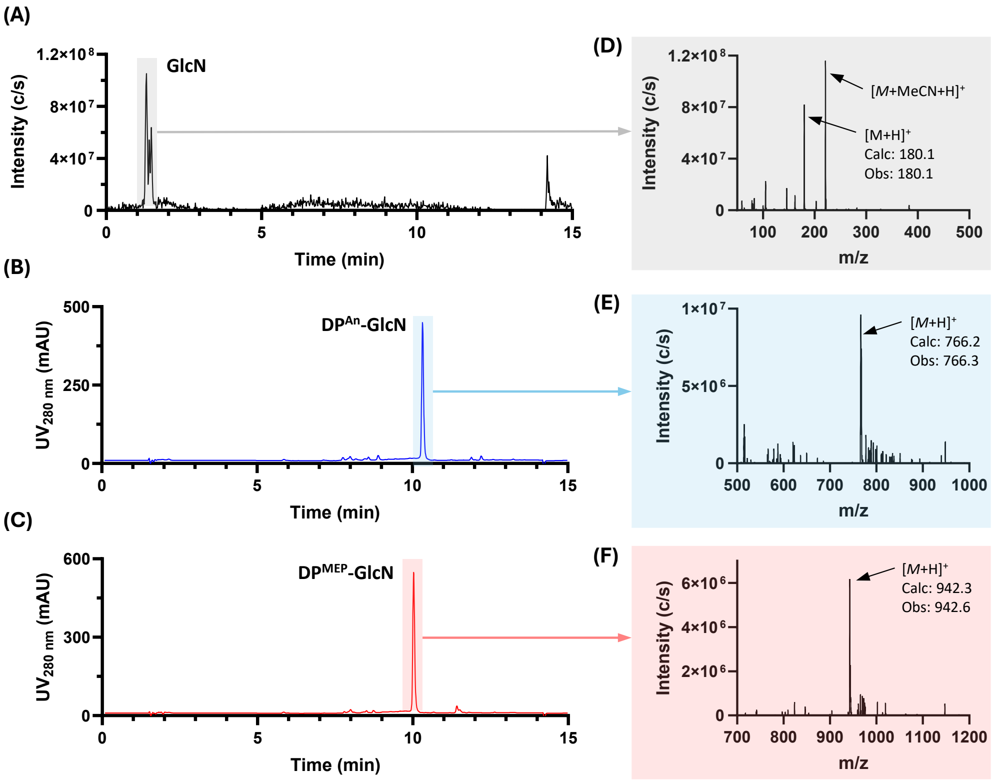

*Figure S13 – Analytical reverse-phase chromatograms showing (A) XIC (180) of GlcN; (B) UV$_{280\,nm}$ of **DP^{An}** and GlcN reaction mixture; (C) UV$_{280\,nm}$ of **DP^{MEP}** and GlcN reaction mixture. MS of (D) GlcN; (E) **DP^{An}-GlcN**; (F) **DP^{MEP}-GlcN**.*

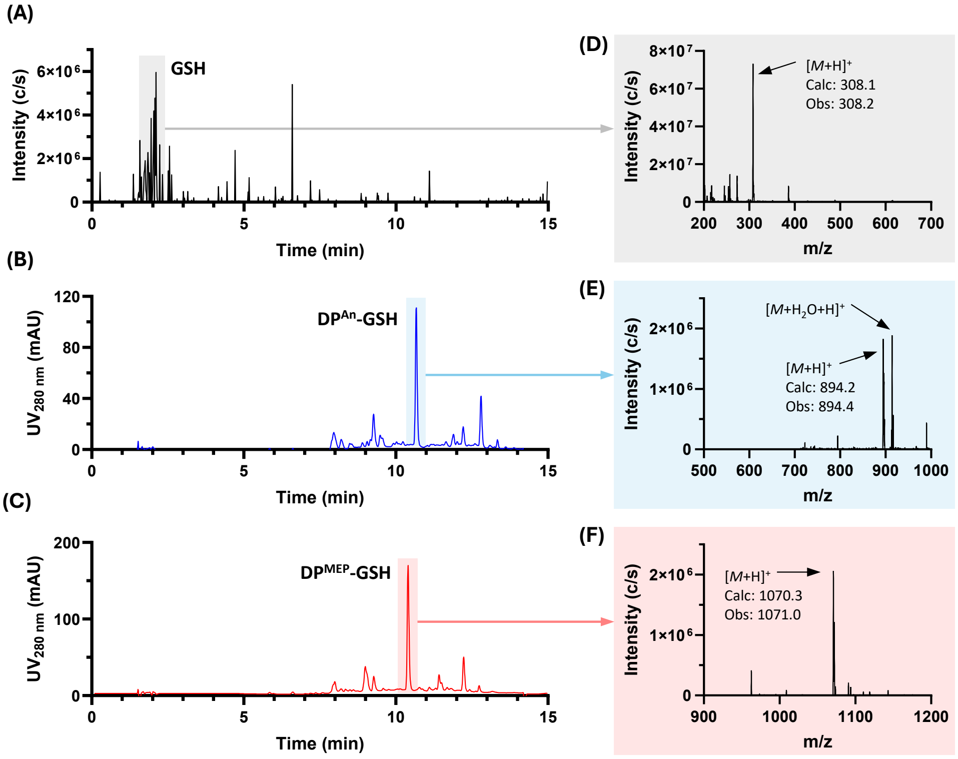

*Figure S14 – Analytical reverse-phase chromatograms showing (A) XIC (308) of GSH; (B) UV$_{280\,nm}$ of **DP^{An}** and GSH reaction mixture; (C) UV$_{280\,nm}$ of **DP^{MEP}** and GSH reaction mixture. MS of (D) GSH; (E) **DP^{An}-GSH**; (F) **DP^{MEP}-GSH**.*

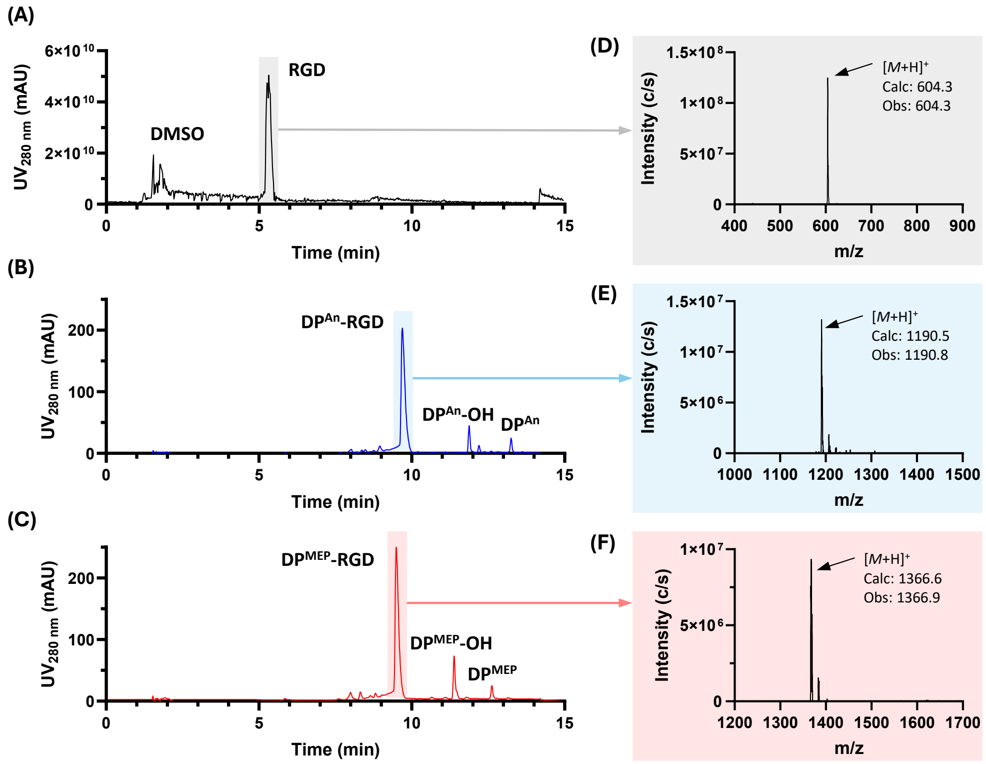

Figure S15 – Analytical reverse-phase chromatograms showing (A) TIC of RGD; (B) UV$_{280\,nm}$ of ***DP^{An}*** *and RGD reaction mixture; (C) UV$_{280\,nm}$ of* ***DP^{MEP}*** *and RGD reaction mixture. MS of (D) RGD; (E)* ***DP^{An}-RGD****; (F)* ***DP^{MEP}-RGD****.*

7 Additional data from **DP^{An}** thiol reactions

Three reactions were undertaken where: **DP^{An}** was combined with 1-propanethiol without DIPEA to ascertain the role that the base plays in reactivity; then **DP^{An}** was combined with 1-propanethiol *or* glutathione in the presence of DIPEA. The general experimental procedure is outlined in Section 3.11. The NMR spectroscopy samples were prepared under an argon atmosphere in young's NMR tubes to minimise phosphine oxidation over time. The $^{31}P\{^1H\}$ NMR spectra show no observable reactivity between thiol and **DP^{An}** without the presence of DIPEA (Figure S16). When DIPEA is present, multiple species are observed (Figure S17), including remaining **DP^{An}** and a major contaminant with doublets at δ_P +32.7 and −3.3 ppm ($J_{P,P}$ = 4.8 Hz). In the presence of glutathione (GSH), many P-containing species were observed by $^{31}P\{^1H\}$ NMR spectroscopy. Notably, two species were observed in the expected region for the desired conjugated product **DP^{An}-GSH** (Figure S18, δ_P −15 to −21 ppm), the two species could be explained by some dimerisation/oxidation of GSH. Resonances in the downfield region of δ_P +15 to 35 ppm indicate the presence of P^V species, possibly owing to radical phosphorus-sulfur reactions forming $R_3P{=}S$ or R_3P^+ species.

In short, both new **DP** compounds react with thiols to give multiple products: consideration should be taken when a biomolecule with a free thiol group is reacted with these diphosphine compounds.

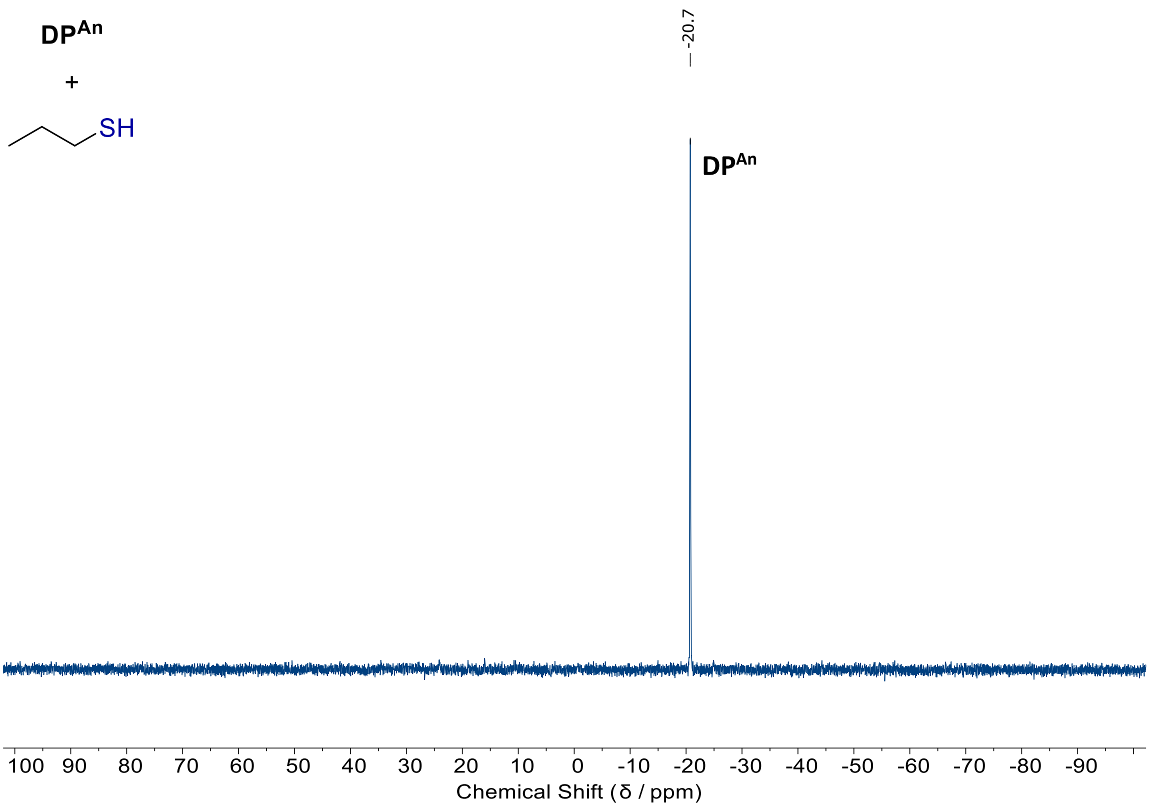

*Figure S16 – $^{31}P\{^{1}H\}$ NMR spectrum in DMF-d_7 of **DP^{An}** and 1-propanethiol without the presence of DIPEA, showing no reactivity between the phosphine and thiol in this case.*

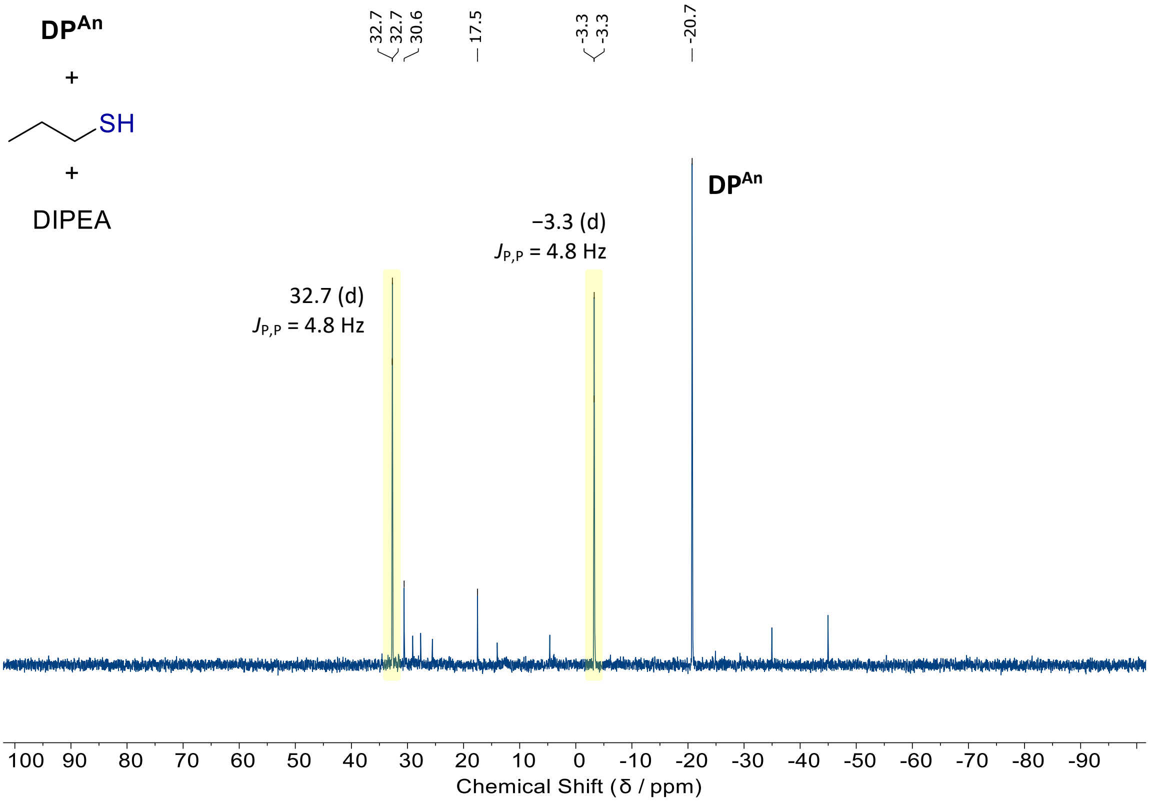

*Figure S17 – $^{31}P\{^{1}H\}$ NMR spectrum in DMF-d_7 of **DP^{An}** and 1-propanethiol in the presence of DIPEA. Some reactivity is evident, with the major side-product being highlighted.*

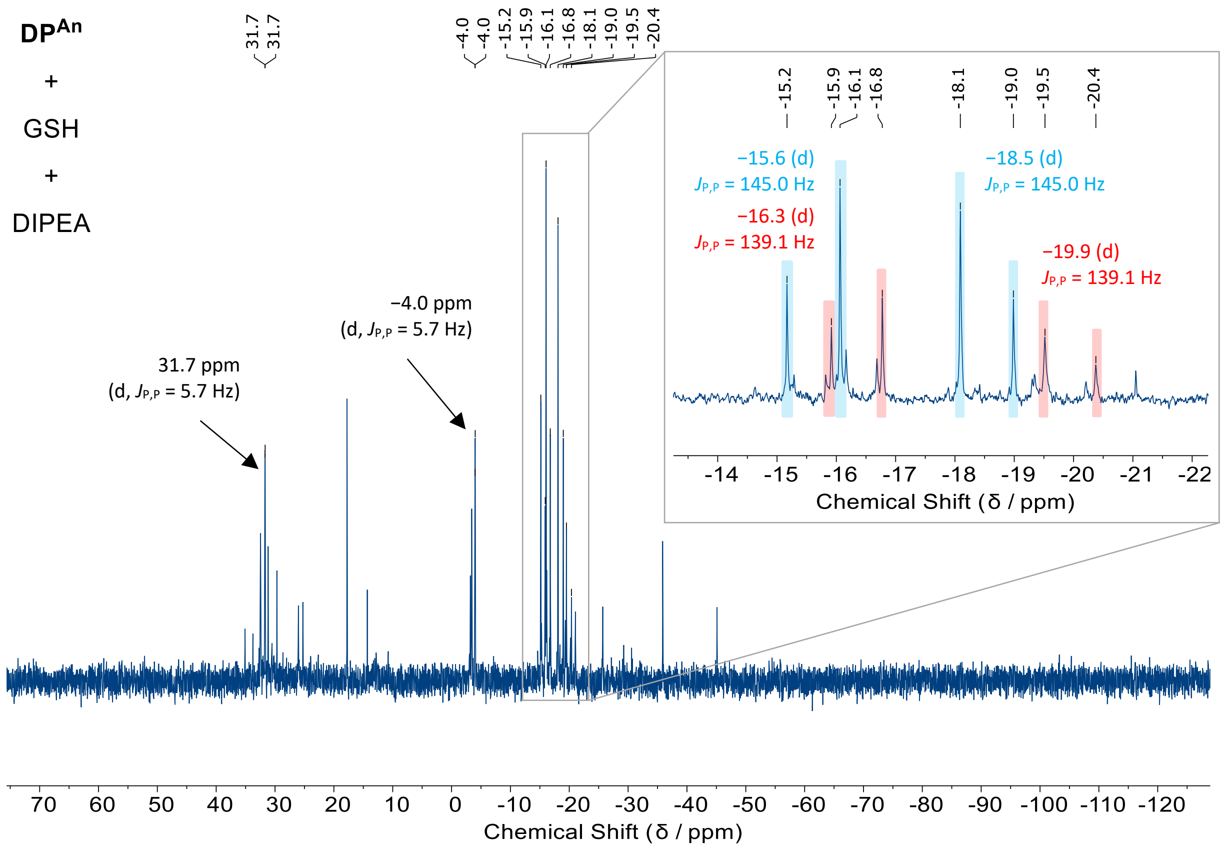

*Figure S18 – $^{31}P\{^{1}H\}$ NMR spectrum in DMF-d_7 of **DP^{An}** and GSH in the presence of DIPEA. Reactivity is evident, with the notable species highlighted.*

8 NMR spectra

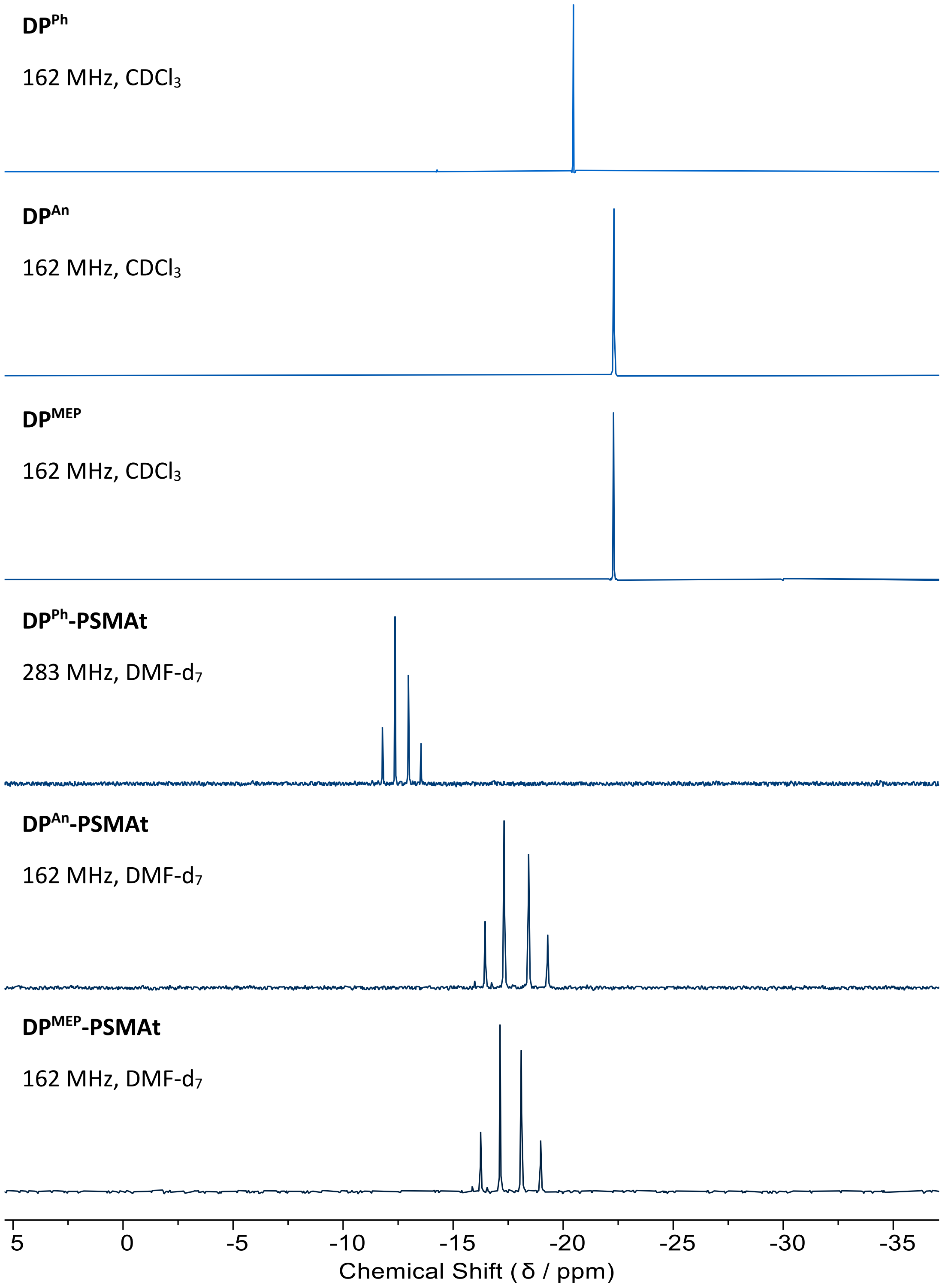

*Figure S19 – $^{31}P\{^{1}H\}$ NMR spectra of **DP**^{X} diphosphine ligands and their respective **DP**^{X}**-PSMAt** bioconjugate derivatives.*

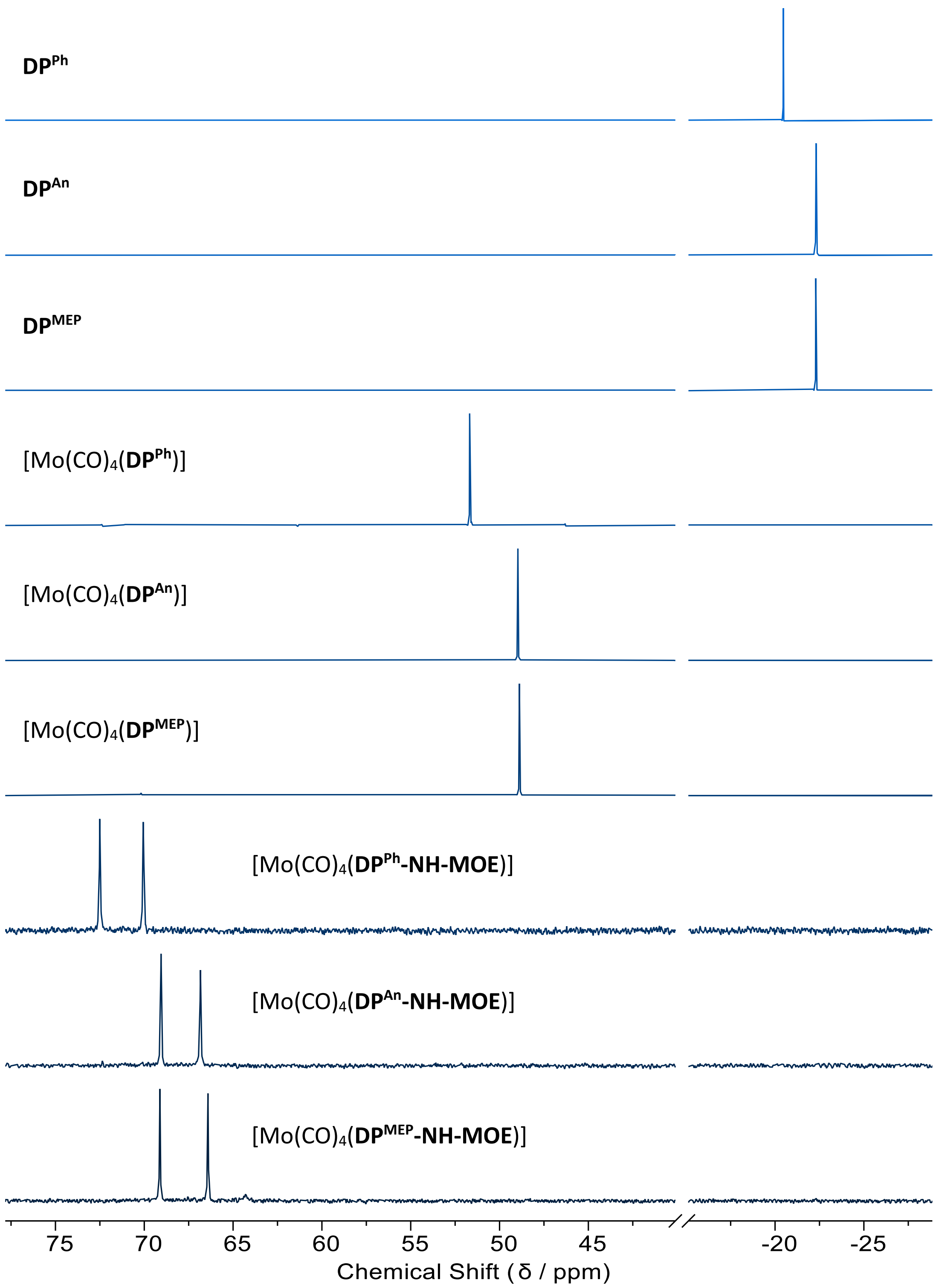

*Figure S20 – $^{31}P\{^1H\}$ NMR spectra of **DP^X** diphosphine ligands and their respective [Mo(CO)$_4$(**DP^X**)] and [Mo(CO)$_4$(**DP^X-NH-MOE**)] complexes.*

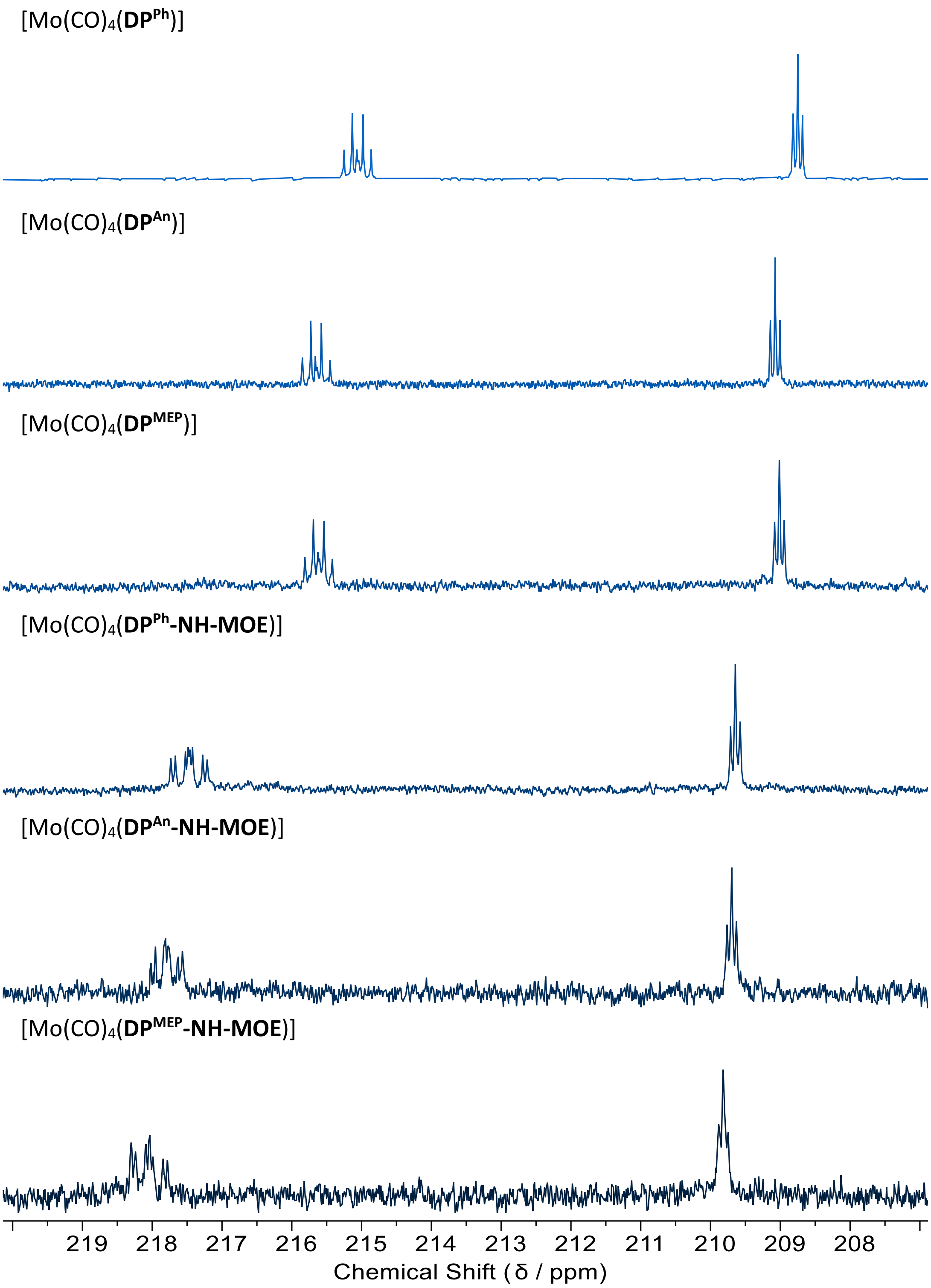

*Figure S21 – $^{13}C\{^{1}H\}$ NMR spectra (126 MHz) of [Mo(CO)$_4$(**DP^X**)] complexes in $CDCl_3$ and [Mo(CO)$_4$(**DP^X-NH-MOE**)] complexes in CD_2Cl_2.*

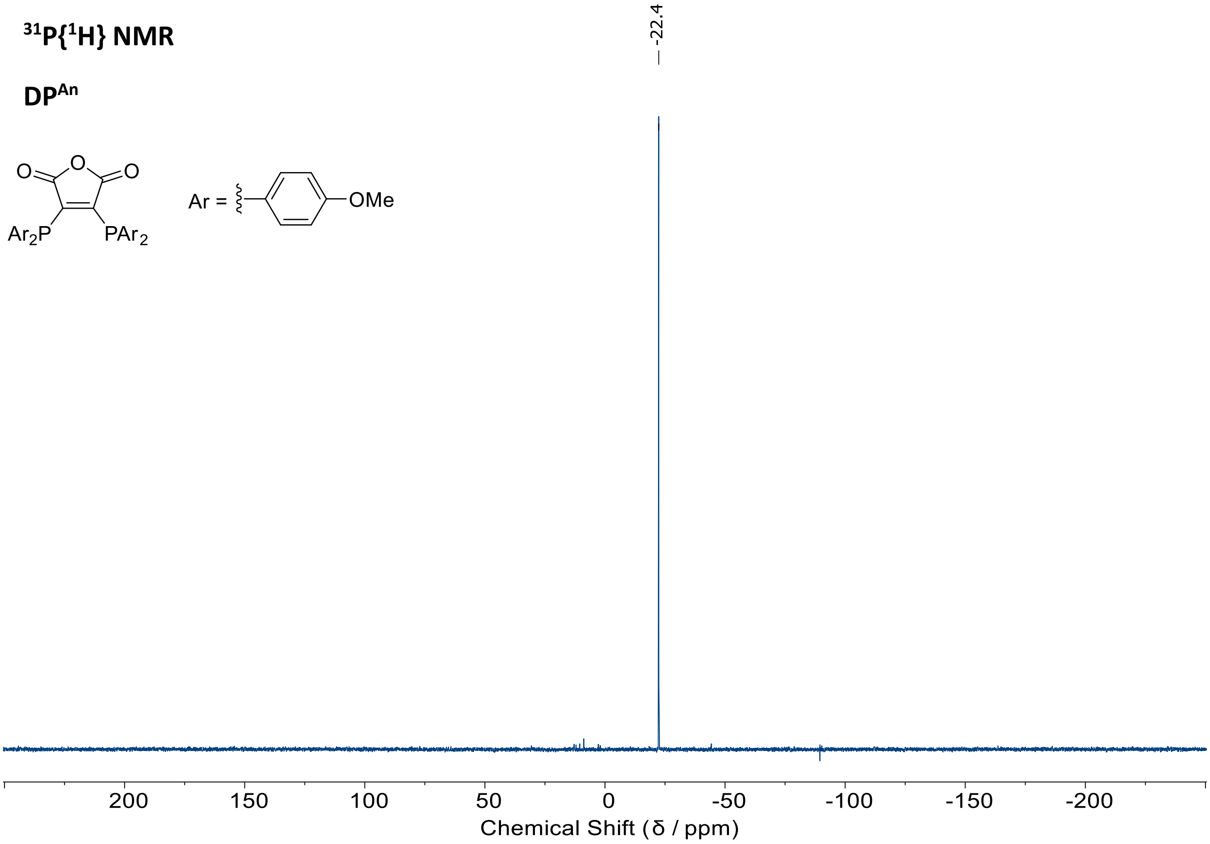
$^{31}P\{^1H\}$ NMR
DP^{An}
O
O
O
Ar$_2$P
PAr$_2$
Ar =
OMe
-22.4
200
150
100
50
0
-50
-100
-150
-200
Chemical Shift (δ / ppm)

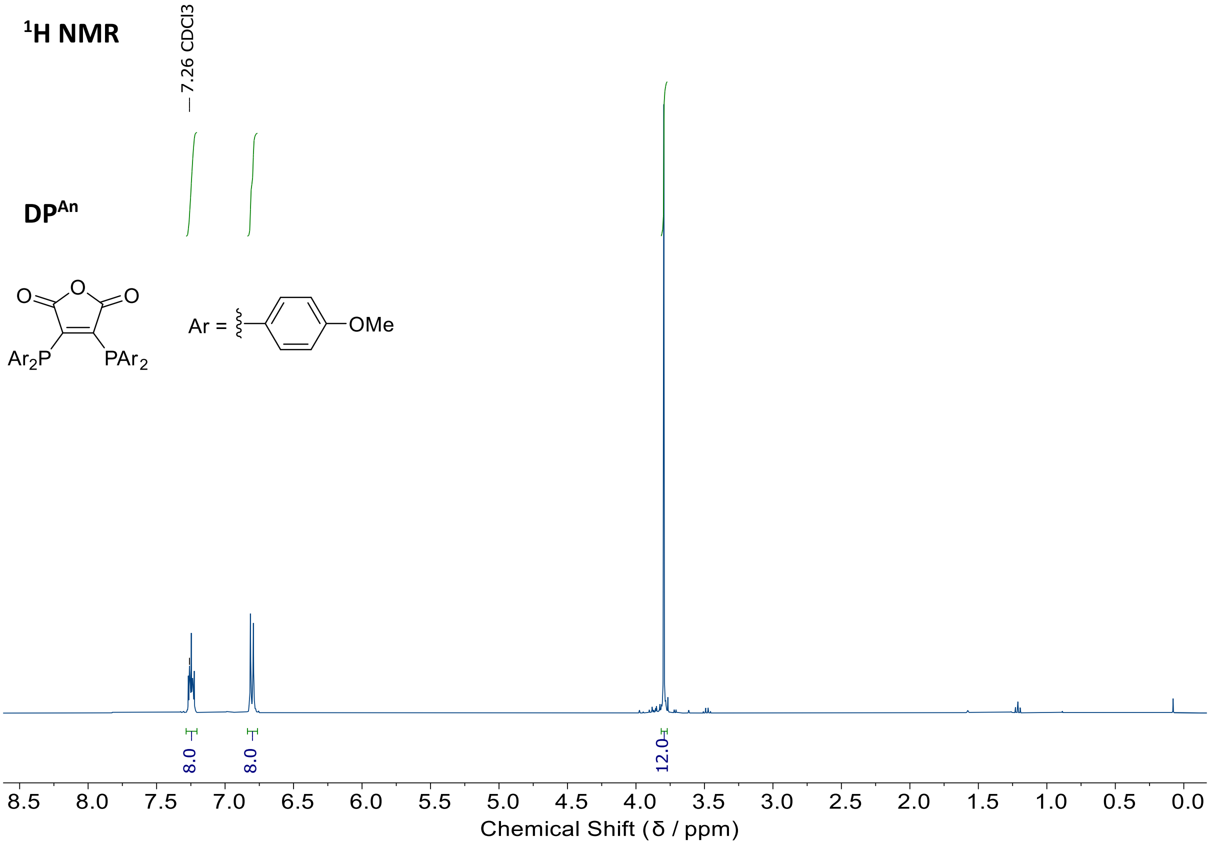
^1H NMR
7.26 CDCl3
DP^{An}
O
O
O
Ar$_2$P
PAr$_2$
Ar =
OMe
8.0
8.0
12.0
8.5
8.0
7.5
7.0
6.5
6.0
5.5
5.0
4.5
4.0
3.5
3.0
2.5
2.0
1.5
1.0
0.5
0.0
Chemical Shift (δ / ppm)

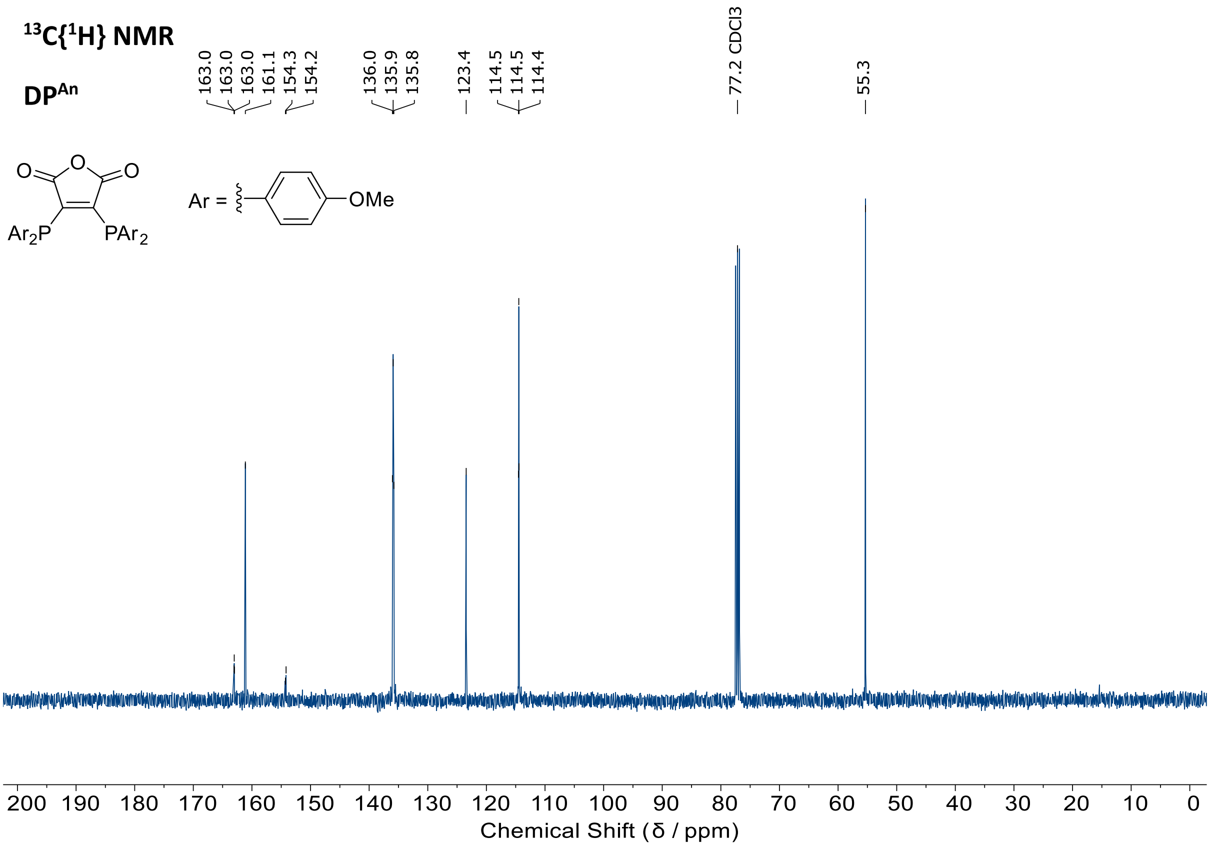
13C{1H} NMR
DPAn
Ar2P
PAr2
Ar =
OMe
163.0
163.0
163.0
161.1
154.3
154.2
136.0
135.9
135.8
123.4
114.5
114.5
114.4
77.2 CDCl3
55.3
200 190 180 170 160 150 140 130 120 110 100 90 80 70 60 50 40 30 20 10 0
Chemical Shift (δ / ppm)

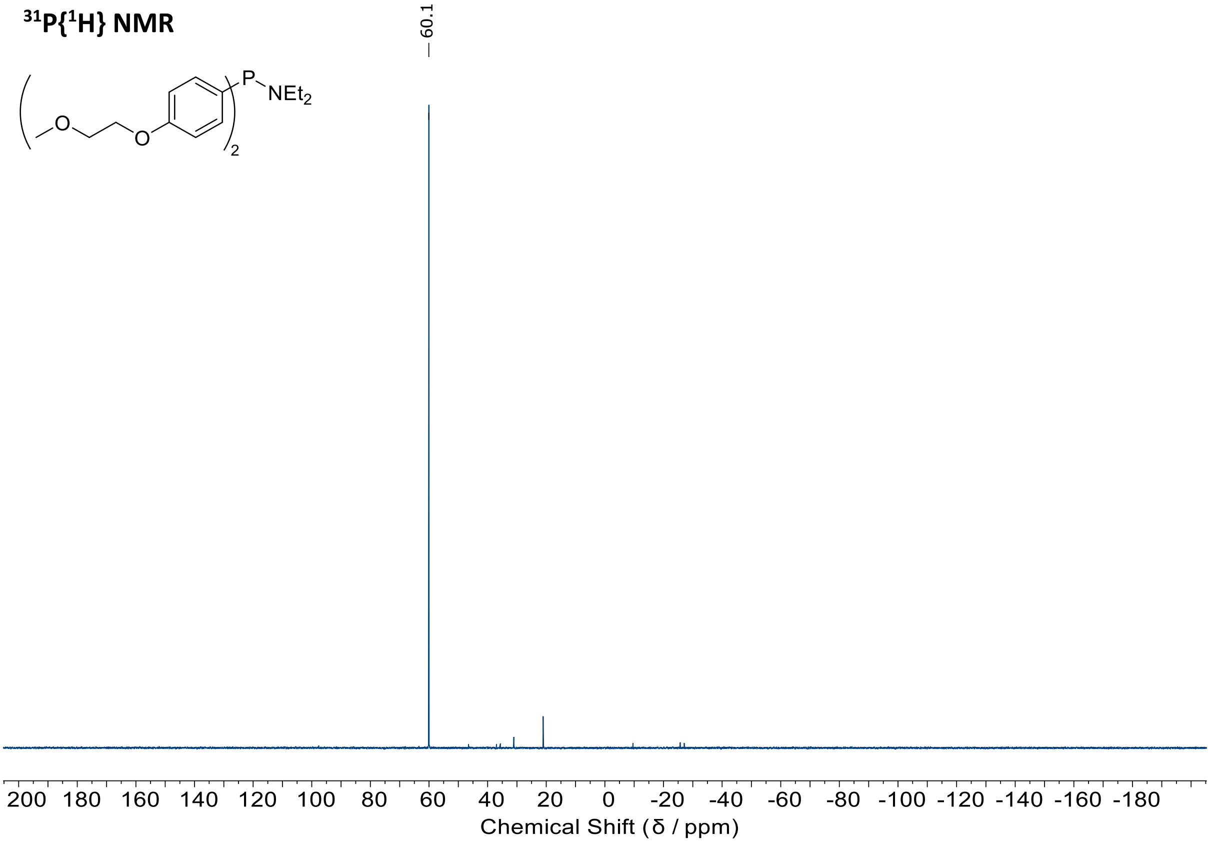
31P{1H} NMR
NEt2
60.1
200 180 160 140 120 100 80 60 40 20 0 -20 -40 -60 -80 -100 -120 -140 -160 -180
Chemical Shift (δ / ppm)

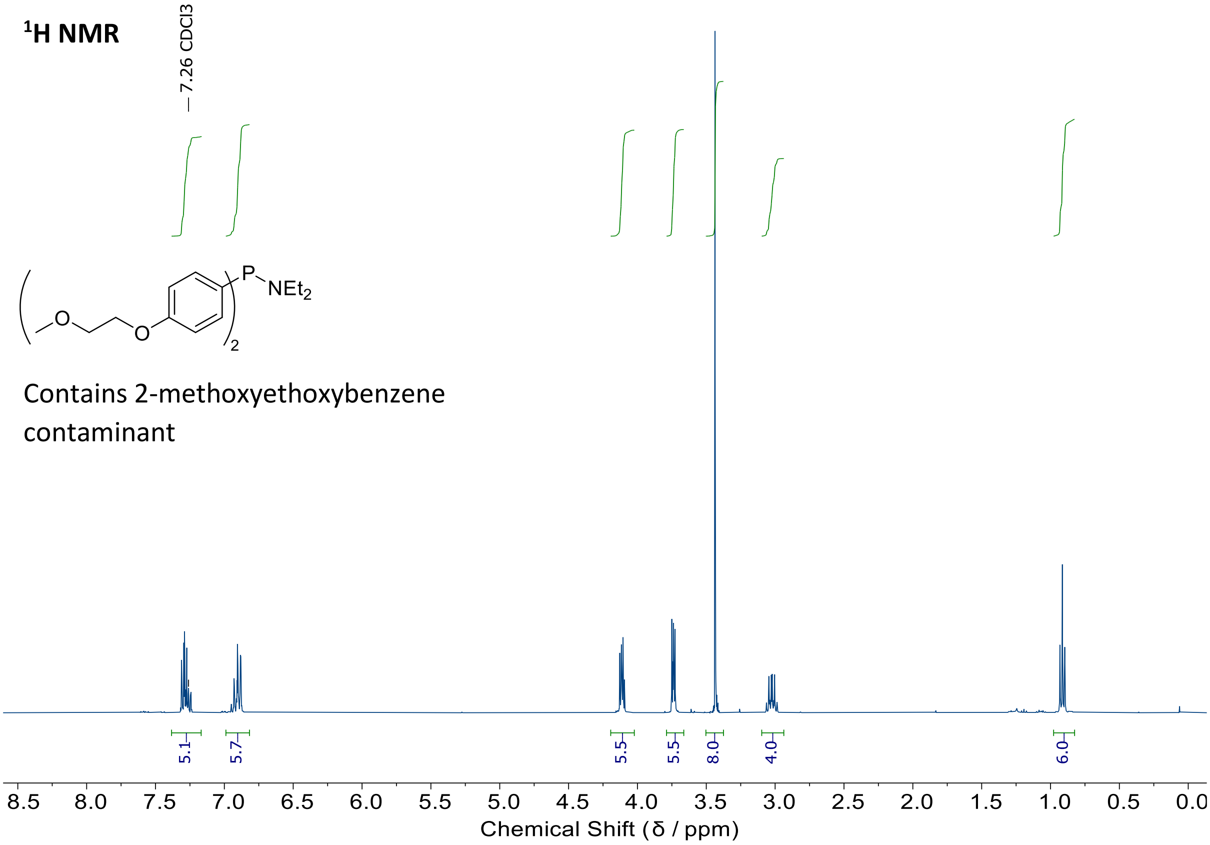

1H NMR
7.26 CDCl3
P
NEt2
O
O
2
Contains 2-methoxyethoxybenzene contaminant
5.1
5.7
5.5
5.5
8.0
4.0
6.0
8.5 8.0 7.5 7.0 6.5 6.0 5.5 5.0 4.5 4.0 3.5 3.0 2.5 2.0 1.5 1.0 0.5 0.0
Chemical Shift (δ / ppm)

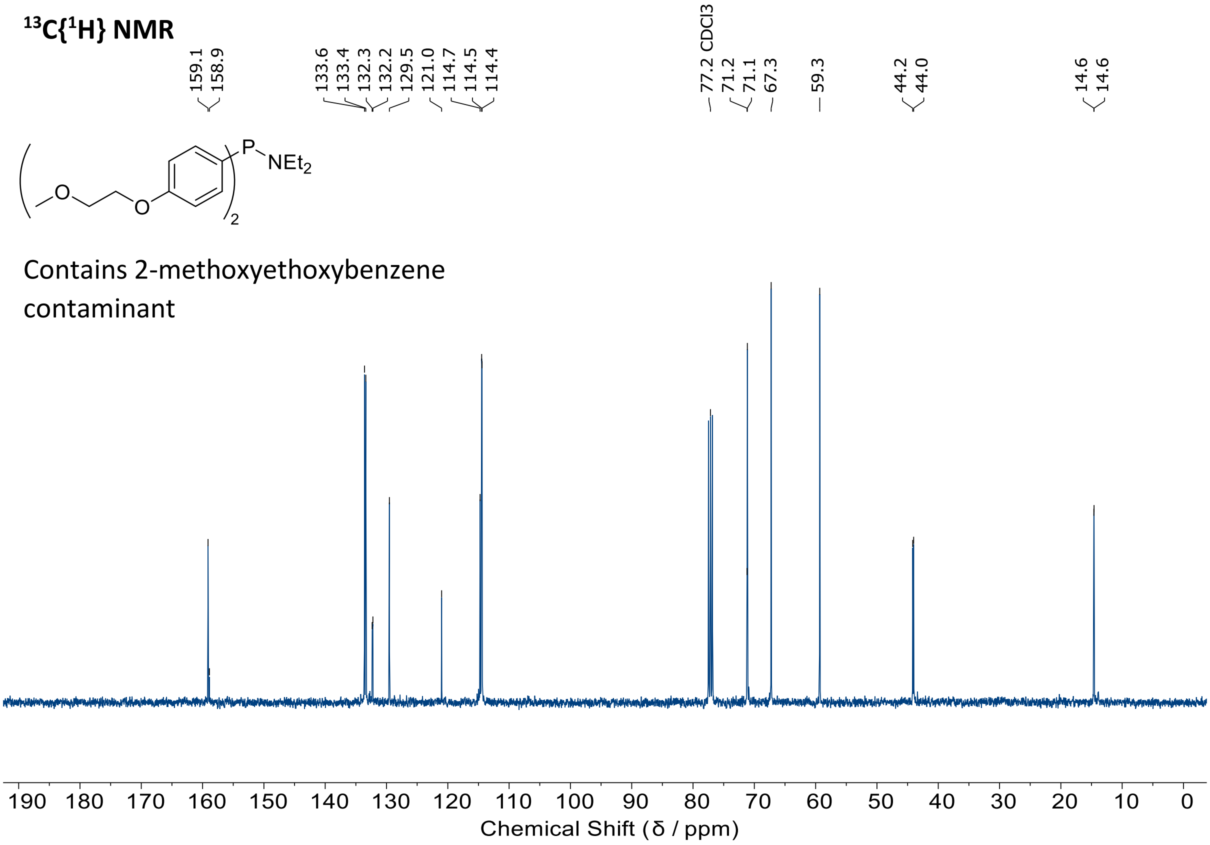

13C{1H} NMR
159.1
158.9
133.6
133.4
132.3
132.2
129.5
121.0
114.7
114.5
114.4
77.2 CDCl3
71.2
71.1
67.3
59.3
44.2
44.0
14.6
14.6
P
NEt2
O
O
2
Contains 2-methoxyethoxybenzene contaminant
190 180 170 160 150 140 130 120 110 100 90 80 70 60 50 40 30 20 10 0
Chemical Shift (δ / ppm)

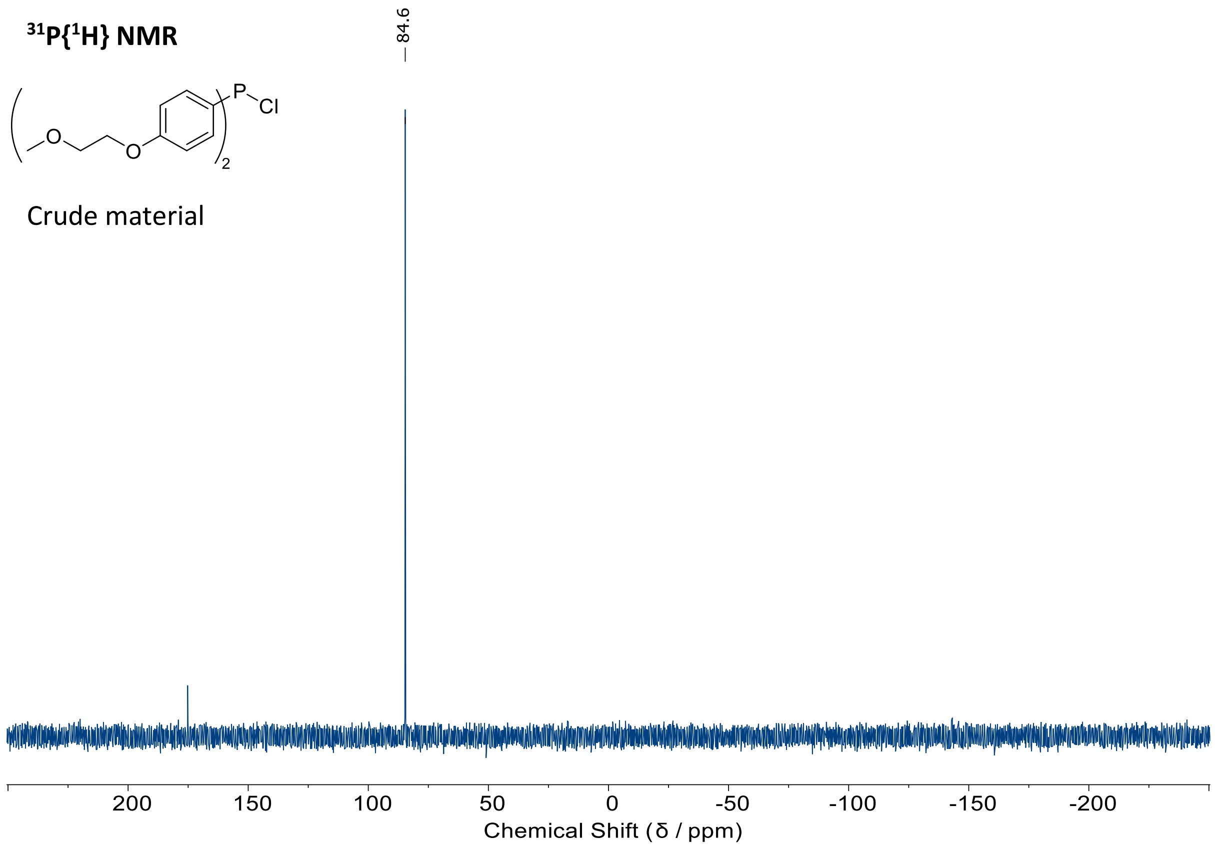
31P{1H} NMR
P
Cl
O
O
2
Crude material
84.6
200
150
100
50
0
-50
-100
-150
-200
Chemical Shift (δ / ppm)

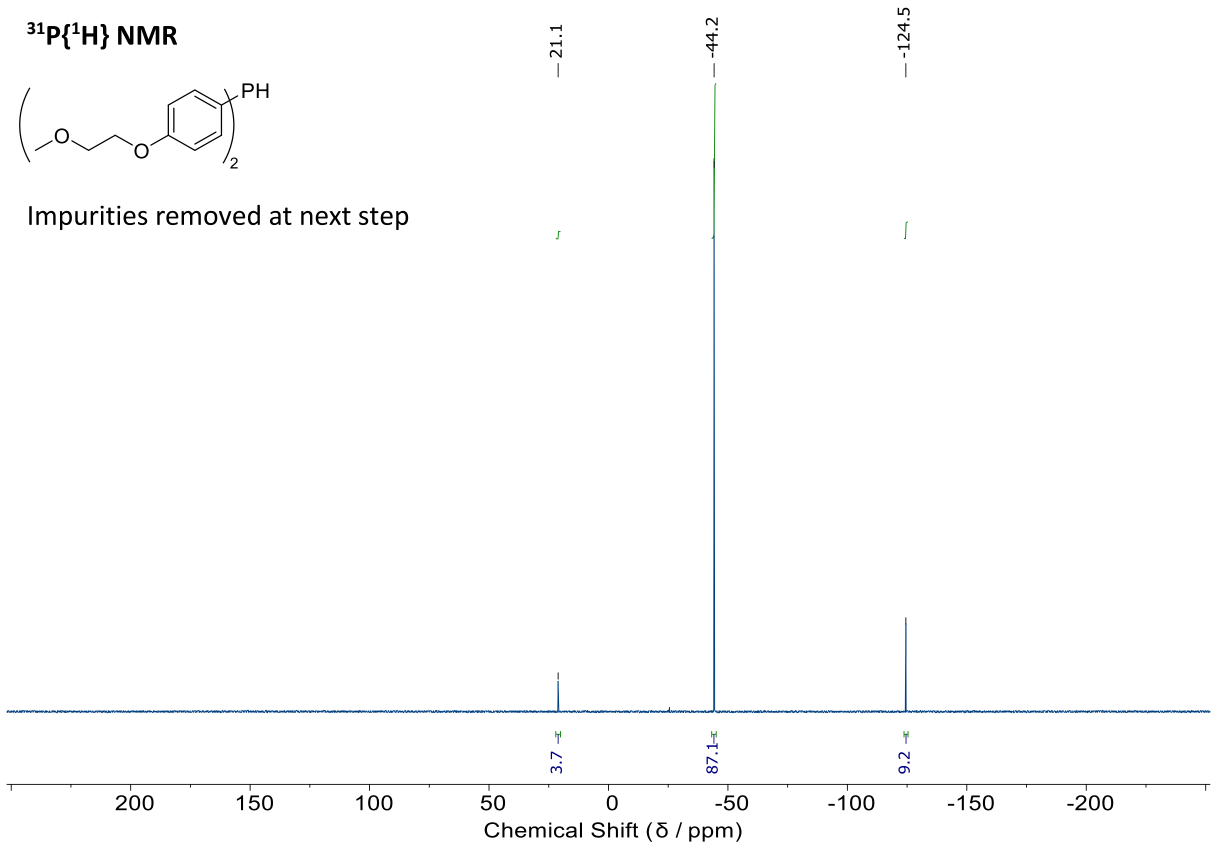
31P{1H} NMR
PH
O
O
2
Impurities removed at next step
21.1
-44.2
-124.5
3.7
87.1
9.2
200
150
100
50
0
-50
-100
-150
-200
Chemical Shift (δ / ppm)

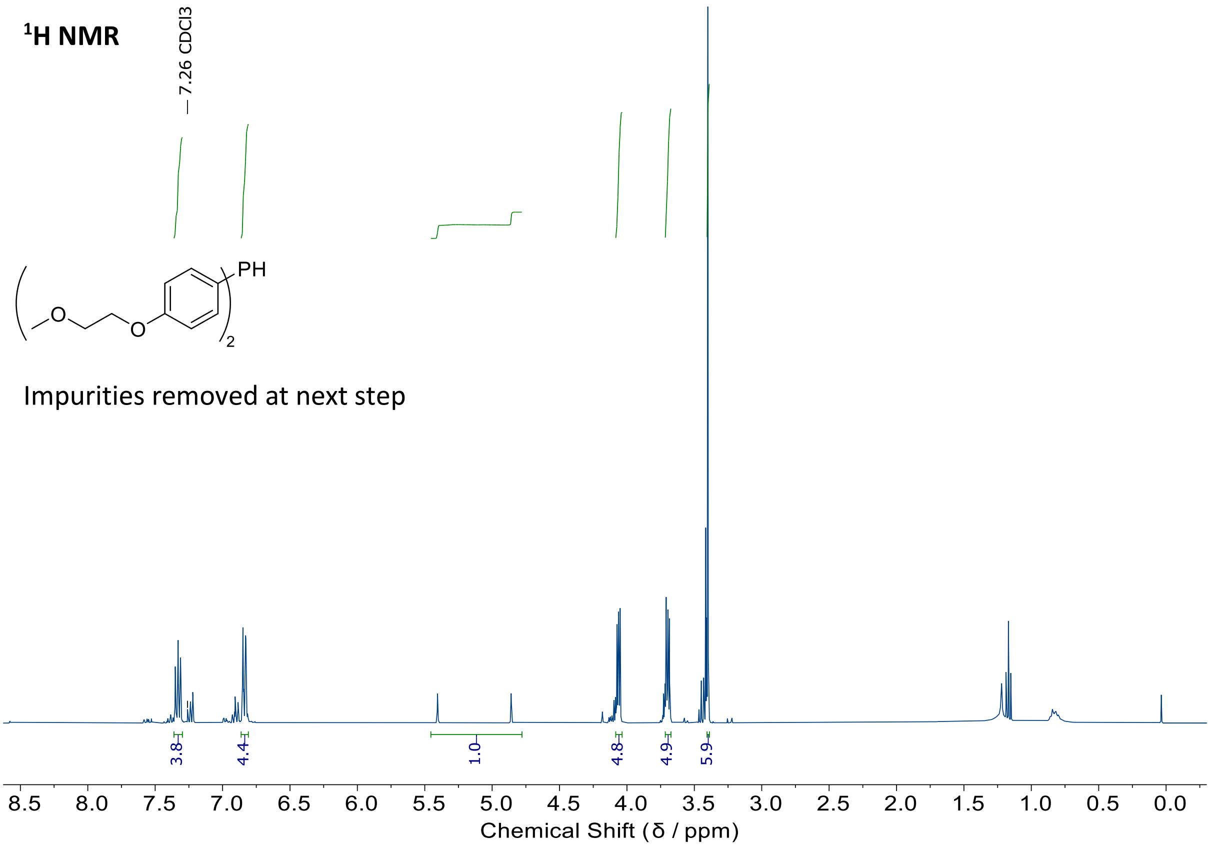

¹H NMR
7.26 CDCl3
PH
2
Impurities removed at next step
3.8
4.4
1.0
4.8
4.9
5.9
8.5 8.0 7.5 7.0 6.5 6.0 5.5 5.0 4.5 4.0 3.5 3.0 2.5 2.0 1.5 1.0 0.5 0.0
Chemical Shift (δ / ppm)

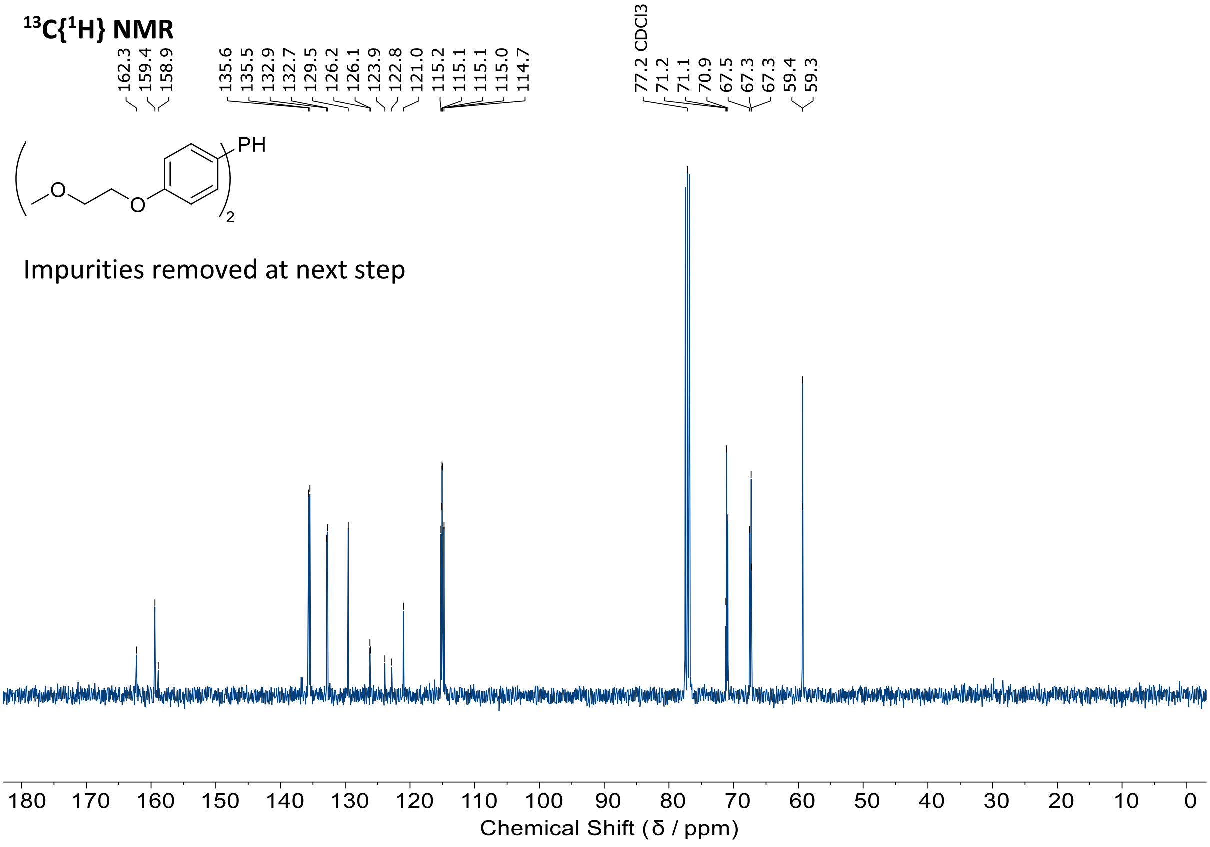

¹³C{¹H} NMR
162.3
159.4
158.9
135.6
135.5
132.9
132.7
129.5
126.2
126.1
123.9
122.8
121.0
115.2
115.1
115.1
115.0
114.7
77.2 CDCl3
71.2
71.1
70.9
67.5
67.3
67.3
59.4
59.3
PH
2
Impurities removed at next step
180 170 160 150 140 130 120 110 100 90 80 70 60 50 40 30 20 10 0
Chemical Shift (δ / ppm)

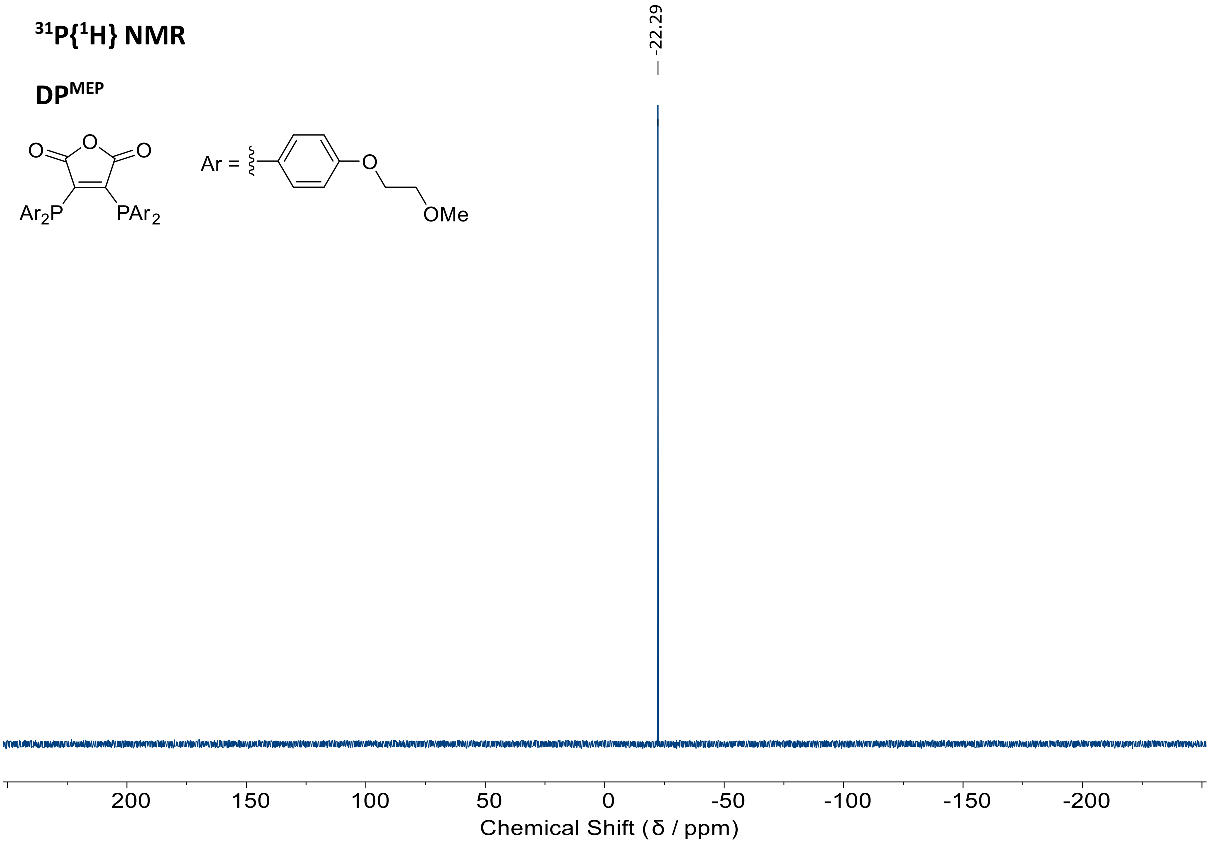

$^{31}P\{^1H\}$ NMR
DP^{MEP}
Ar$_2$P
PAr$_2$
Ar =
OMe
-22.29
200
150
100
50
0
-50
-100
-150
-200
Chemical Shift (δ / ppm)

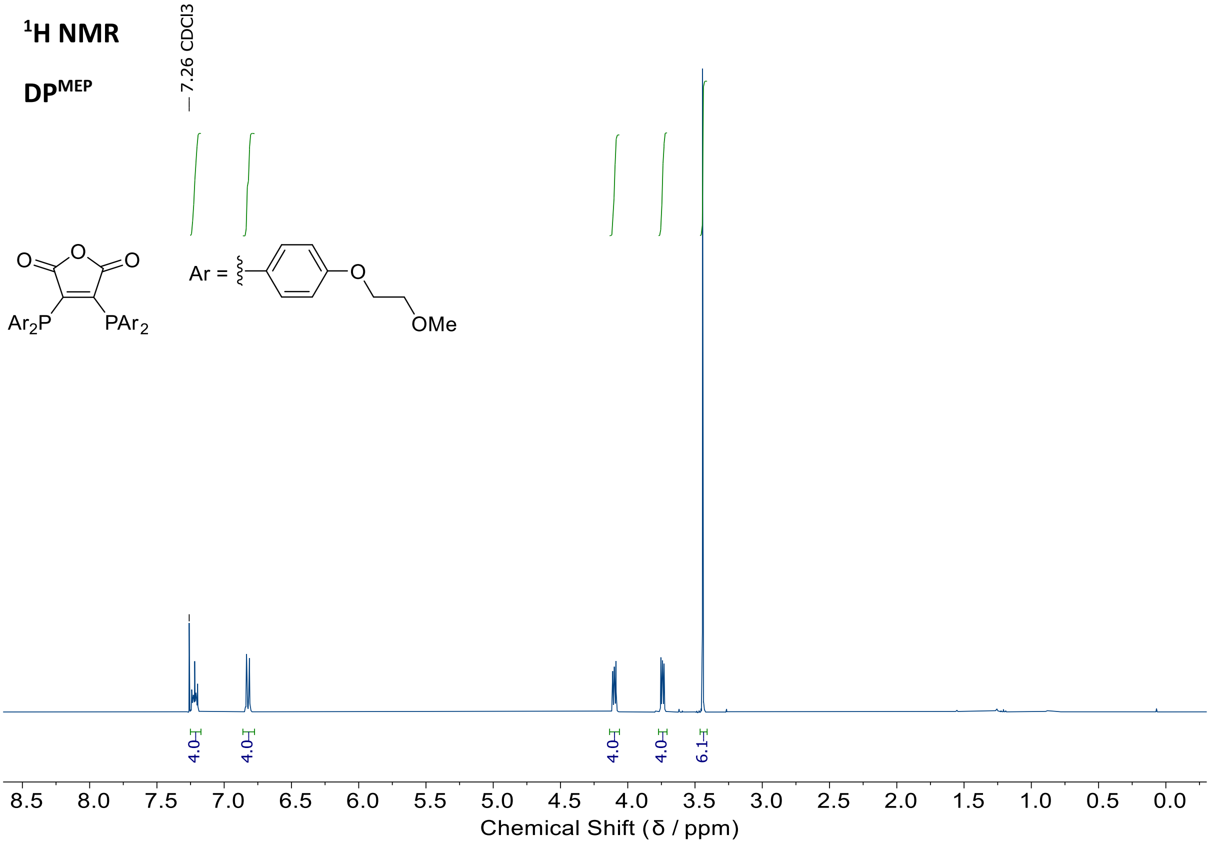

^1H NMR
DP^{MEP}
7.26 CDCl3
Ar$_2$P
PAr$_2$
Ar =
OMe
4.0
4.0
4.0
4.0
6.1
8.5
8.0
7.5
7.0
6.5
6.0
5.5
5.0
4.5
4.0
3.5
3.0
2.5
2.0
1.5
1.0
0.5
0.0
Chemical Shift (δ / ppm)

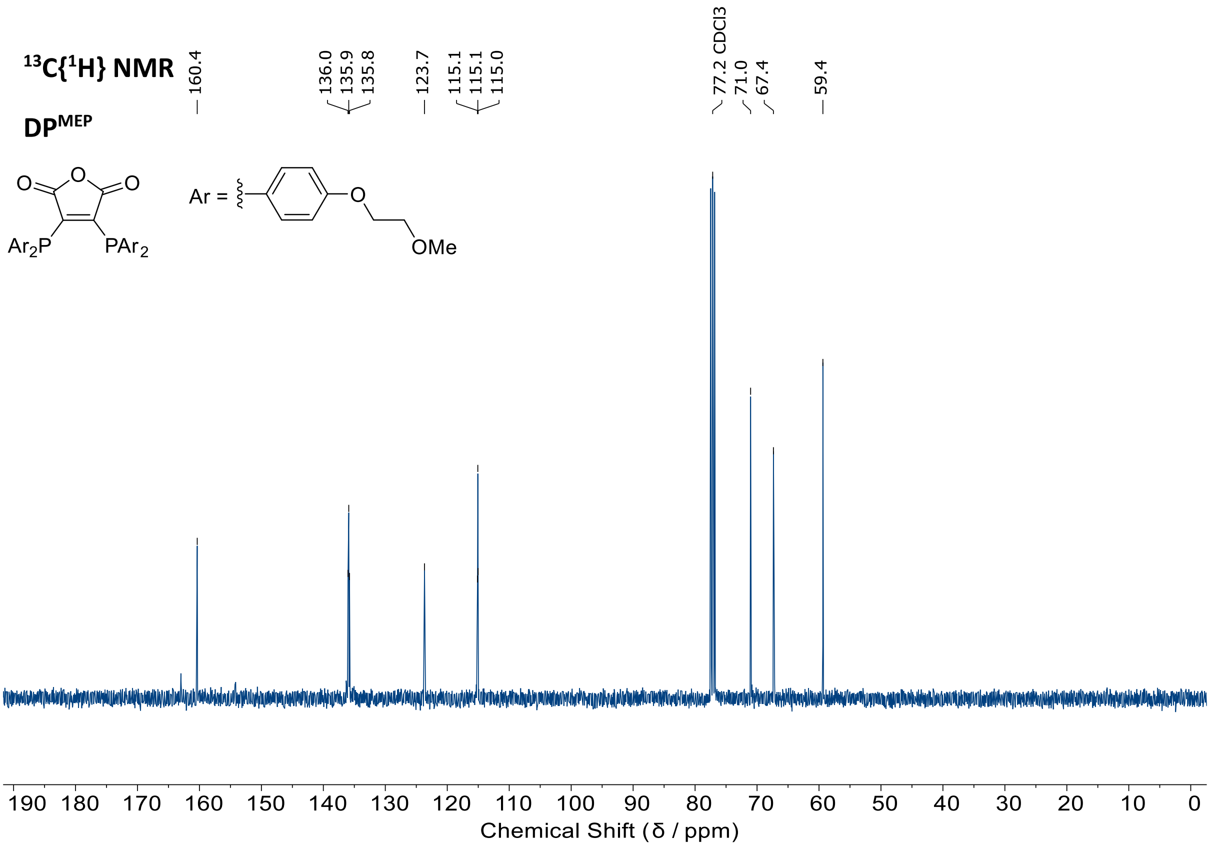

$^{13}C\{^{1}H\}$ NMR
DP^{MEP}
Ar =
OMe
Ar_2P
PAr_2
160.4
136.0
135.9
135.8
123.7
115.1
115.1
115.0
77.2 CDCl3
71.0
67.4
59.4
190 180 170 160 150 140 130 120 110 100 90 80 70 60 50 40 30 20 10 0
Chemical Shift (δ / ppm)

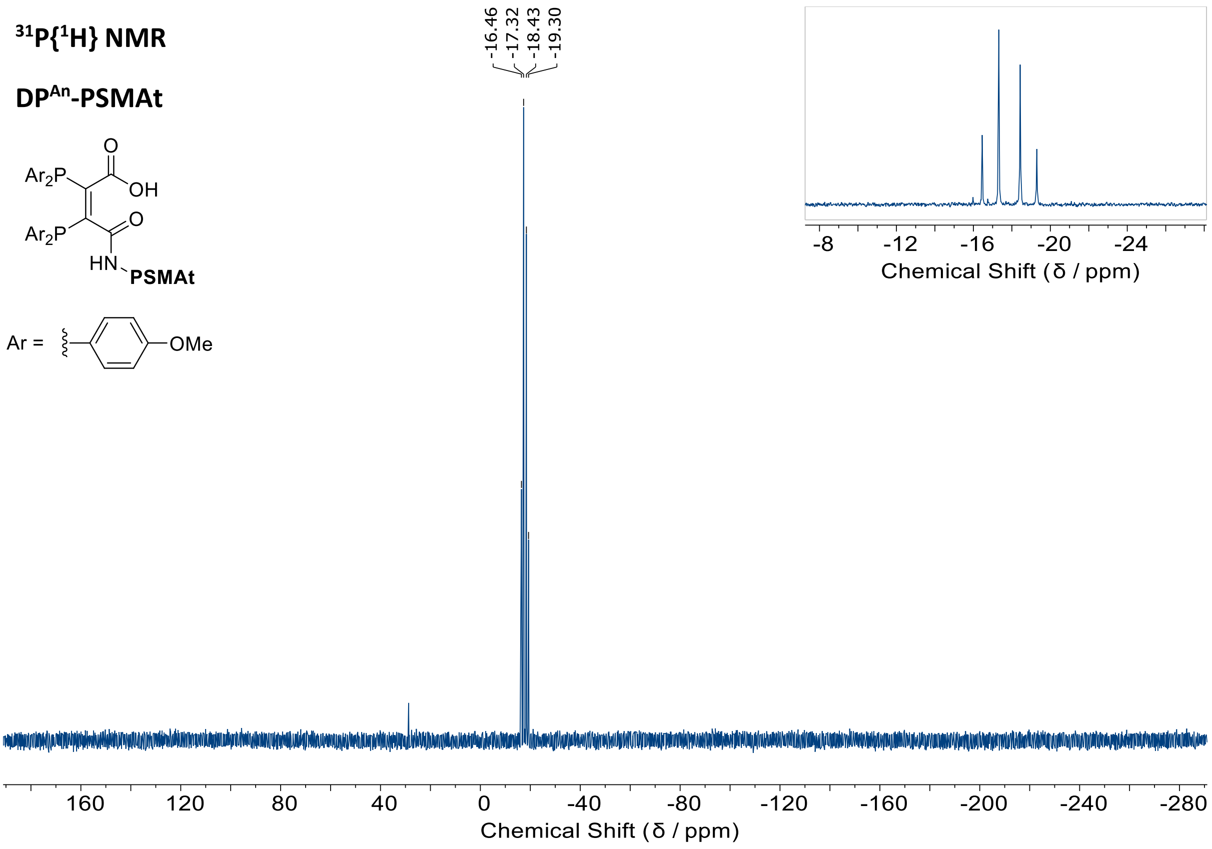
31P{1H} NMR
DPAn-PSMAt
Ar2P
OH
O
Ar2P
O
HN
PSMAt
Ar =
OMe
-16.46
-17.32
-18.43
-19.30
-8
-12
-16
-20
-24
Chemical Shift (δ / ppm)
160
120
80
40
0
-40
-80
-120
-160
-200
-240
-280
Chemical Shift (δ / ppm)

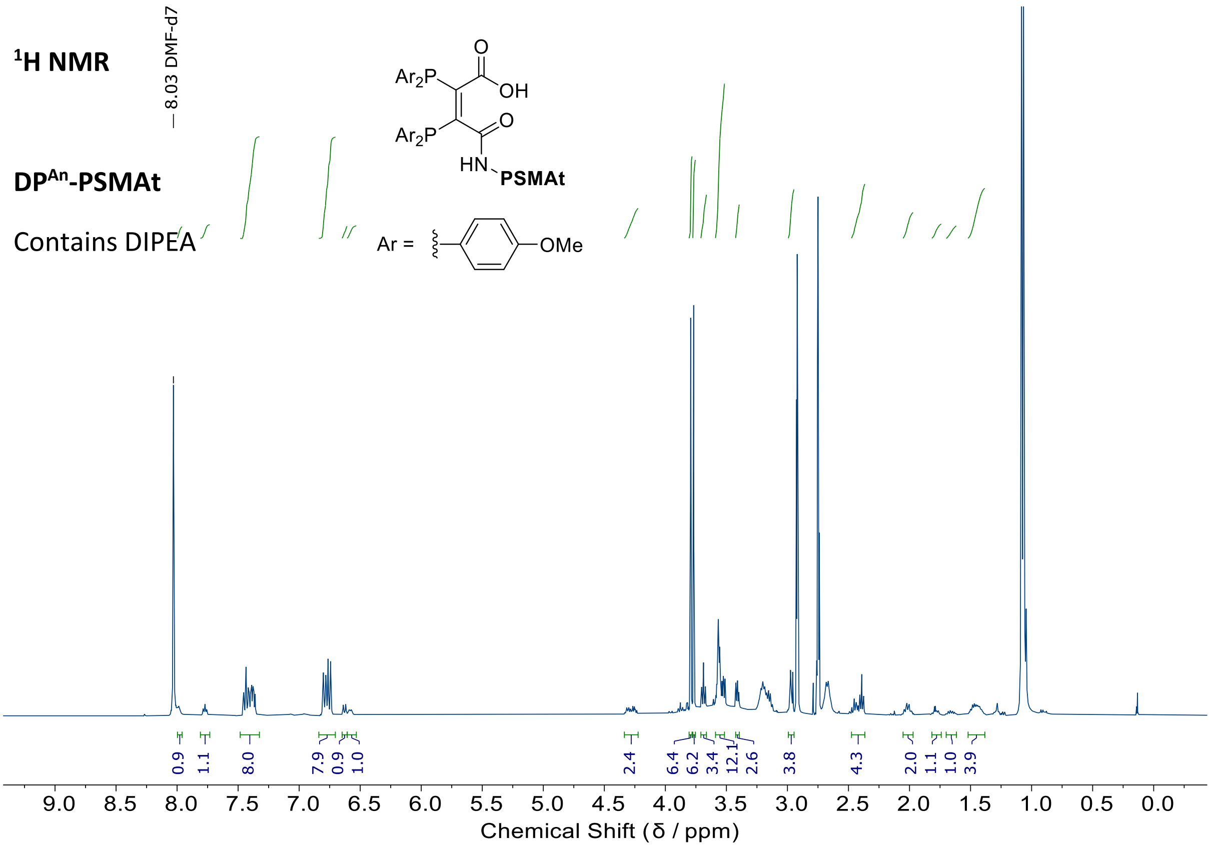
1H NMR
8.03 DMF-d7
DPAn-PSMAt
Contains DIPEA
Ar2P
OH
O
Ar2P
O
HN
PSMAt
Ar =
OMe
0.9
1.1
8.0
7.9
0.9
1.0
2.4
6.4
6.2
3.4
12.1
2.6
3.8
4.3
2.0
1.1
1.0
3.9
9.0
8.5
8.0
7.5
7.0
6.5
6.0
5.5
5.0
4.5
4.0
3.5
3.0
2.5
2.0
1.5
1.0
0.5
0.0
Chemical Shift (δ / ppm)

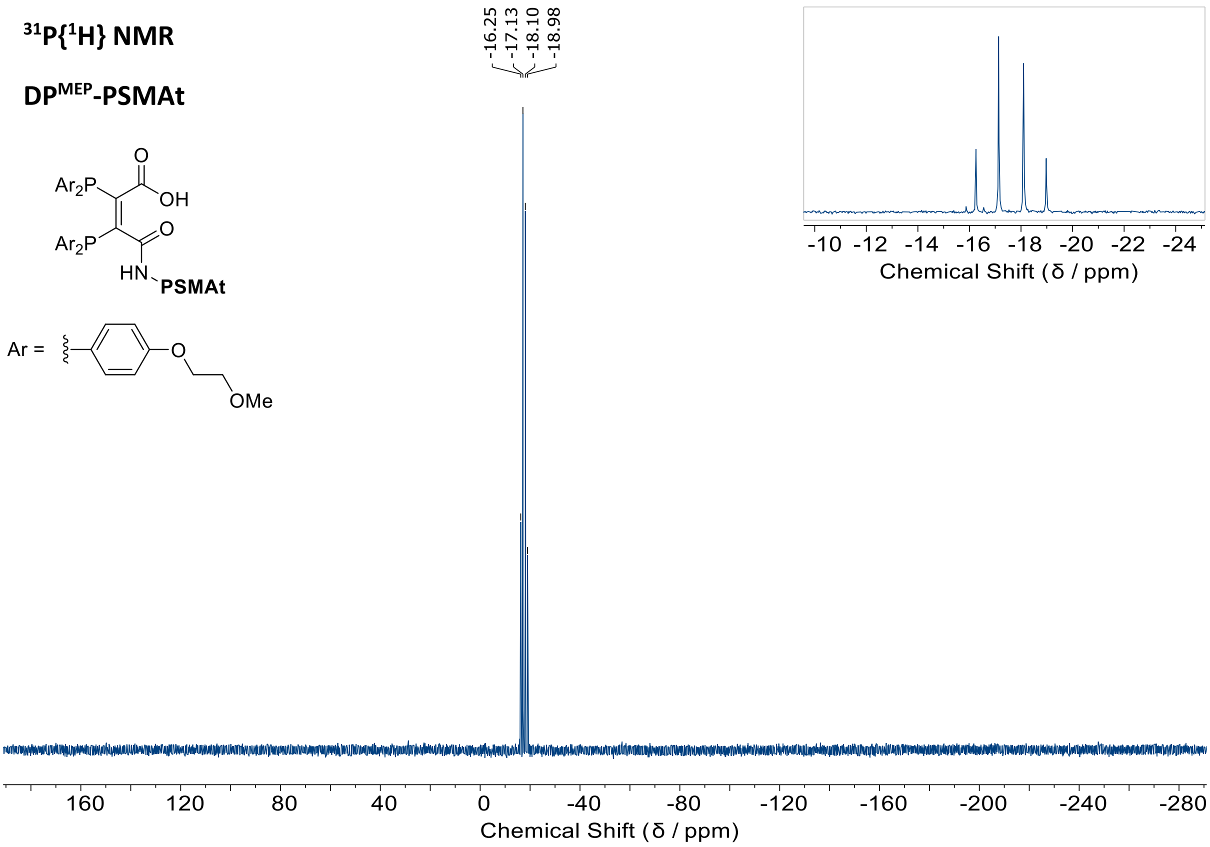
31P{1H} NMR
DPMEP-PSMAt
Ar2P
O
OH
Ar2P
O
HN
PSMAt
Ar =
O
OMe
-16.25
-17.13
-18.10
-18.98
-10 -12 -14 -16 -18 -20 -22 -24
Chemical Shift (δ / ppm)
160 120 80 40 0 -40 -80 -120 -160 -200 -240 -280
Chemical Shift (δ / ppm)

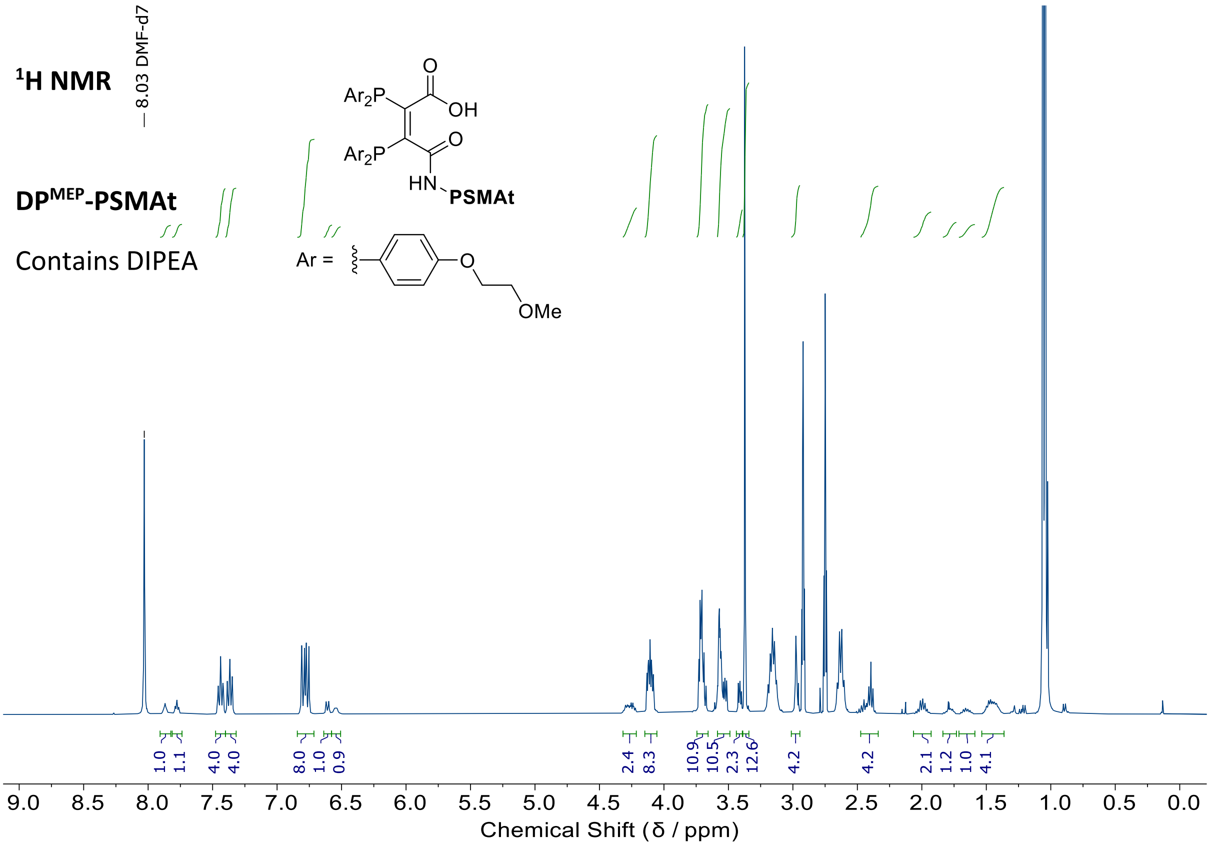
1H NMR
8.03 DMF-d7
DPMEP-PSMAt
Contains DIPEA
Ar2P
O
OH
Ar2P
O
HN
PSMAt
Ar =
O
OMe
1.0 1.1 4.0 4.0 8.0 1.0 0.9 2.4 8.3 10.9 10.5 2.3 12.6 4.2 4.2 2.1 1.2 1.0 4.1
9.0 8.5 8.0 7.5 7.0 6.5 6.0 5.5 5.0 4.5 4.0 3.5 3.0 2.5 2.0 1.5 1.0 0.5 0.0
Chemical Shift (δ / ppm)

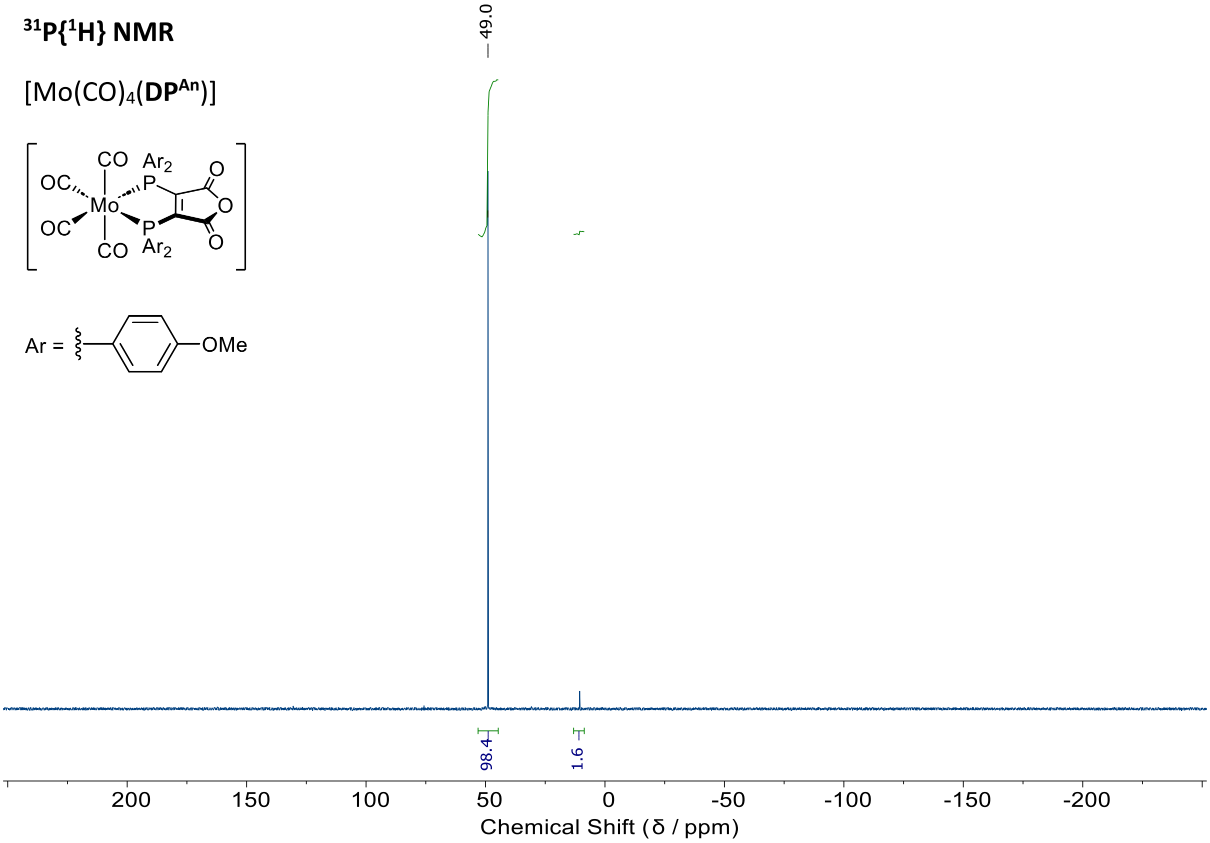
31P{1H} NMR
[Mo(CO)4(DPAn)]
CO
Ar2
OC
Mo
P
O
O
OC
P
CO
Ar2
O
Ar =
OMe
49.0
98.4
1.6
200
150
100
50
0
-50
-100
-150
-200
Chemical Shift (δ / ppm)

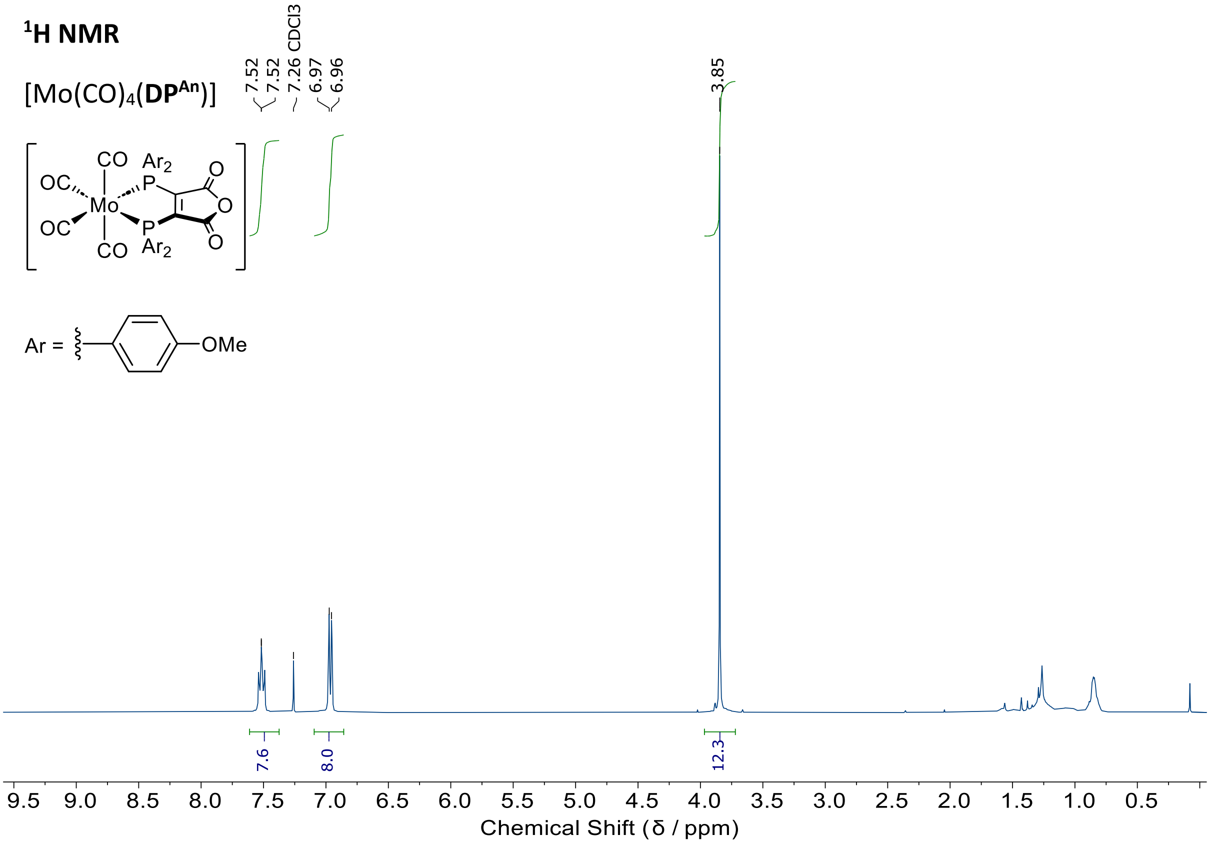
1H NMR
[Mo(CO)4(DPAn)]
CO
Ar2
OC
Mo
P
O
O
OC
P
CO
Ar2
O
Ar =
OMe
7.52
7.52
7.26 CDCl3
6.97
6.96
3.85
7.6
8.0
12.3
9.5
9.0
8.5
8.0
7.5
7.0
6.5
6.0
5.5
5.0
4.5
4.0
3.5
3.0
2.5
2.0
1.5
1.0
0.5
Chemical Shift (δ / ppm)

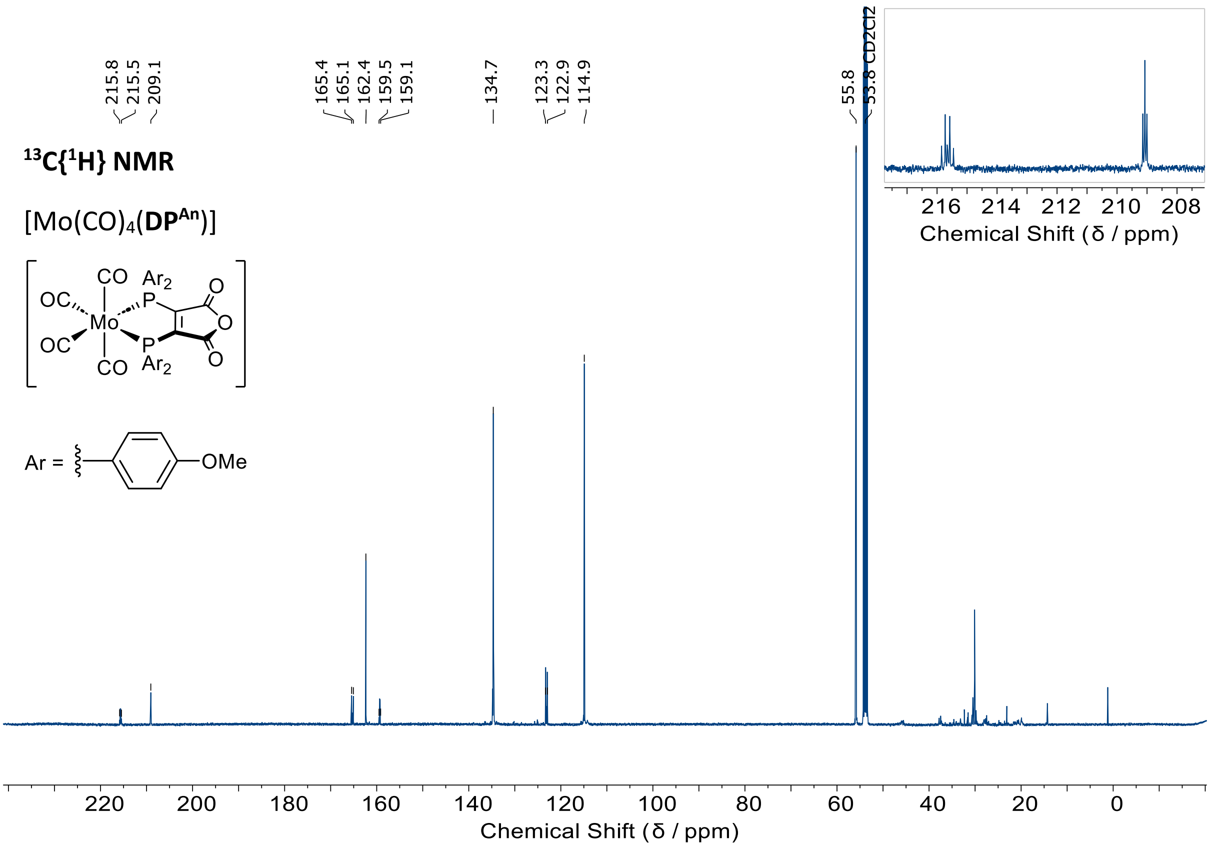
215.8
215.5
209.1
165.4
165.1
162.4
159.5
159.1
134.7
123.3
122.9
114.9
55.8
53.8 CD2Cl2
13C{1H} NMR
[Mo(CO)4(DPAn)]
CO
Ar2
OC
P
O
Mo
O
OC
P
O
CO
Ar2
Ar =
OMe
216
214
212
210
208
Chemical Shift (δ / ppm)
220
200
180
160
140
120
100
80
60
40
20
0
Chemical Shift (δ / ppm)

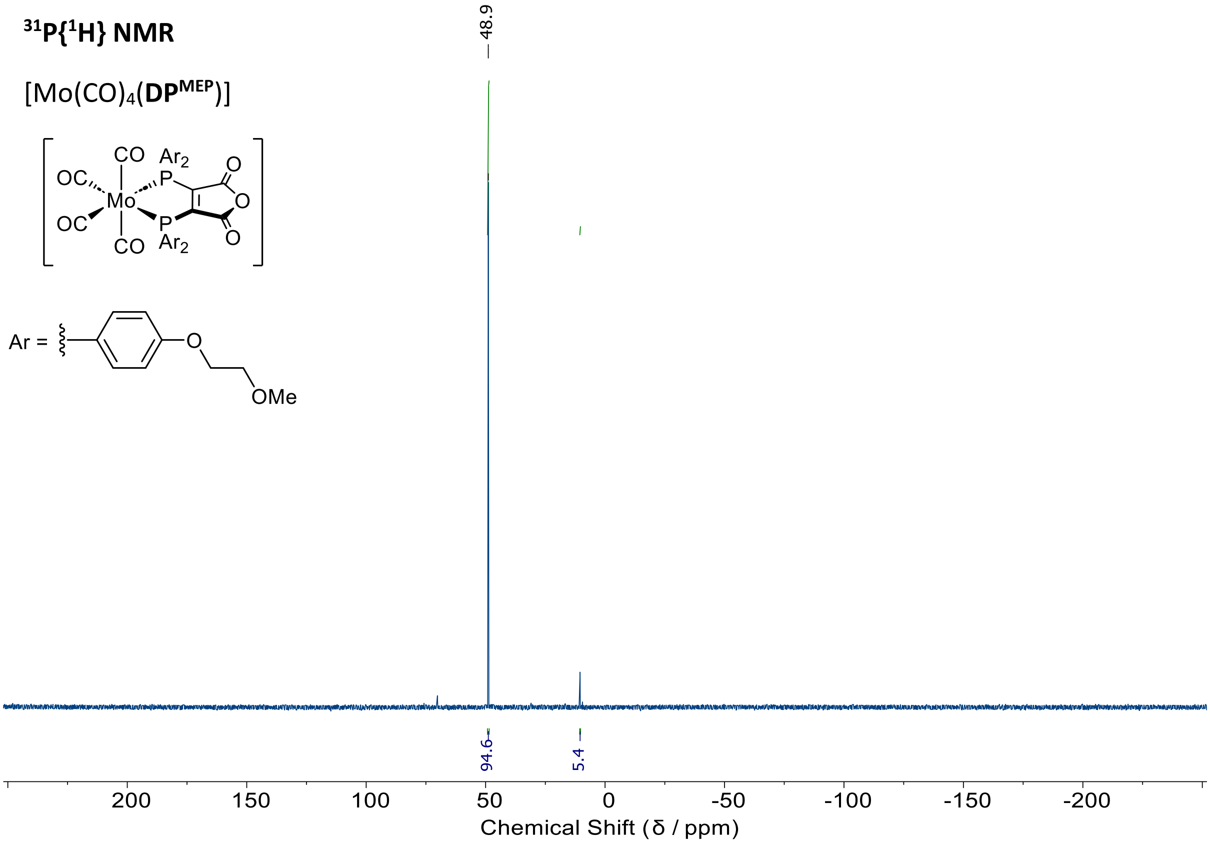
31P{1H} NMR
[Mo(CO)4(DPMEP)]
48.9
CO
Ar2
OC
P
O
Mo
O
OC
P
O
CO
Ar2
Ar =
O
OMe
94.6
5.4
200
150
100
50
0
-50
-100
-150
-200
Chemical Shift (δ / ppm)

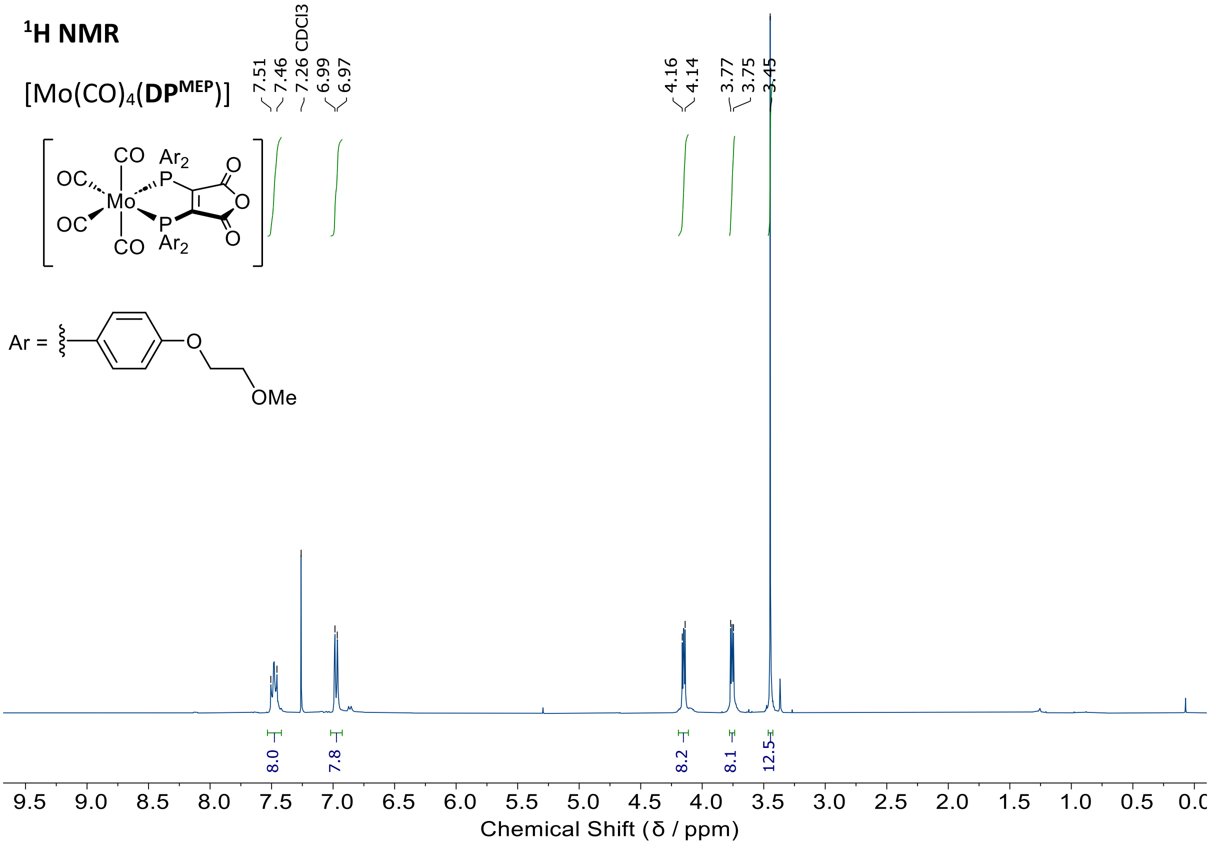
^{1}H NMR
[Mo(CO)$_4$(**DP^{MEP}**)]
CO
Ar$_2$
OC
P
O
Mo
O
OC
P
O
CO
Ar$_2$
Ar =
O
OMe
7.51
7.46
7.26 CDCl3
6.99
6.97
4.16
4.14
3.77
3.75
3.45
8.0
7.8
8.2
8.1
12.5
9.5 9.0 8.5 8.0 7.5 7.0 6.5 6.0 5.5 5.0 4.5 4.0 3.5 3.0 2.5 2.0 1.5 1.0 0.5 0.0
Chemical Shift (δ / ppm)

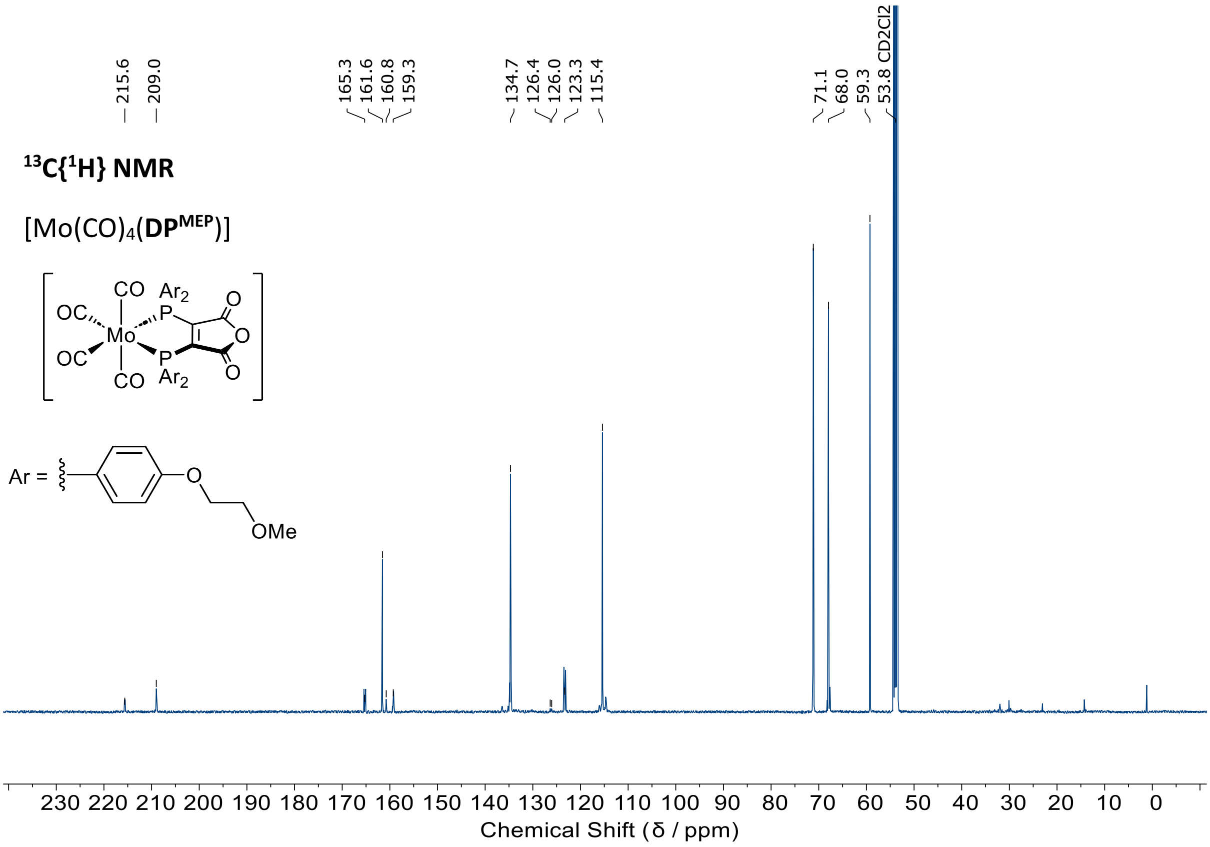
^{13}C{^{1}H} NMR
[Mo(CO)$_4$(**DP^{MEP}**)]
CO
Ar$_2$
OC
P
O
Mo
O
OC
P
O
CO
Ar$_2$
Ar =
O
OMe
215.6
209.0
165.3
161.6
160.8
159.3
134.7
126.4
126.0
123.3
115.4
71.1
68.0
59.3
53.8 CD2Cl2
230 220 210 200 190 180 170 160 150 140 130 120 110 100 90 80 70 60 50 40 30 20 10 0
Chemical Shift (δ / ppm)

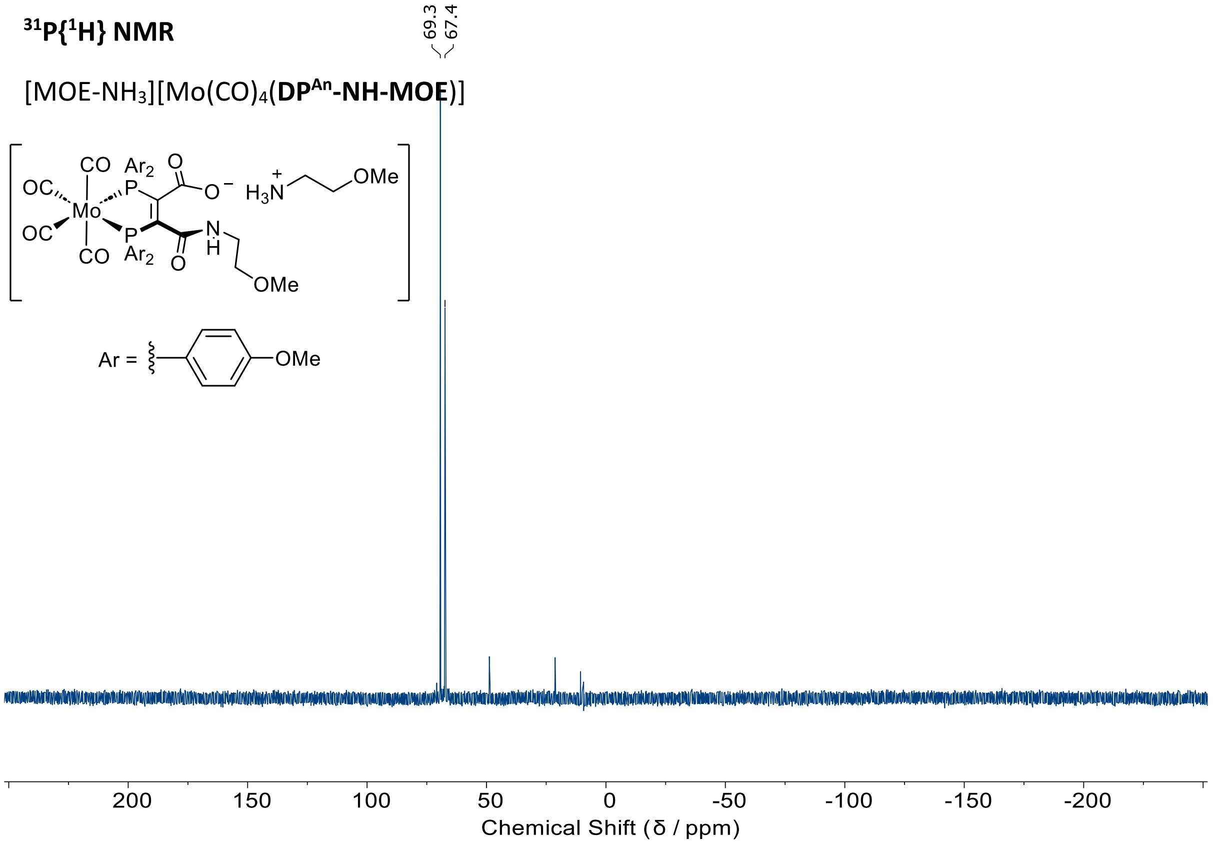
31P{1H} NMR
69.3
67.4
[MOE-NH3][Mo(CO)4(DPAn-NH-MOE)]
Ar = OMe
200 150 100 50 0 -50 -100 -150 -200
Chemical Shift (δ / ppm)

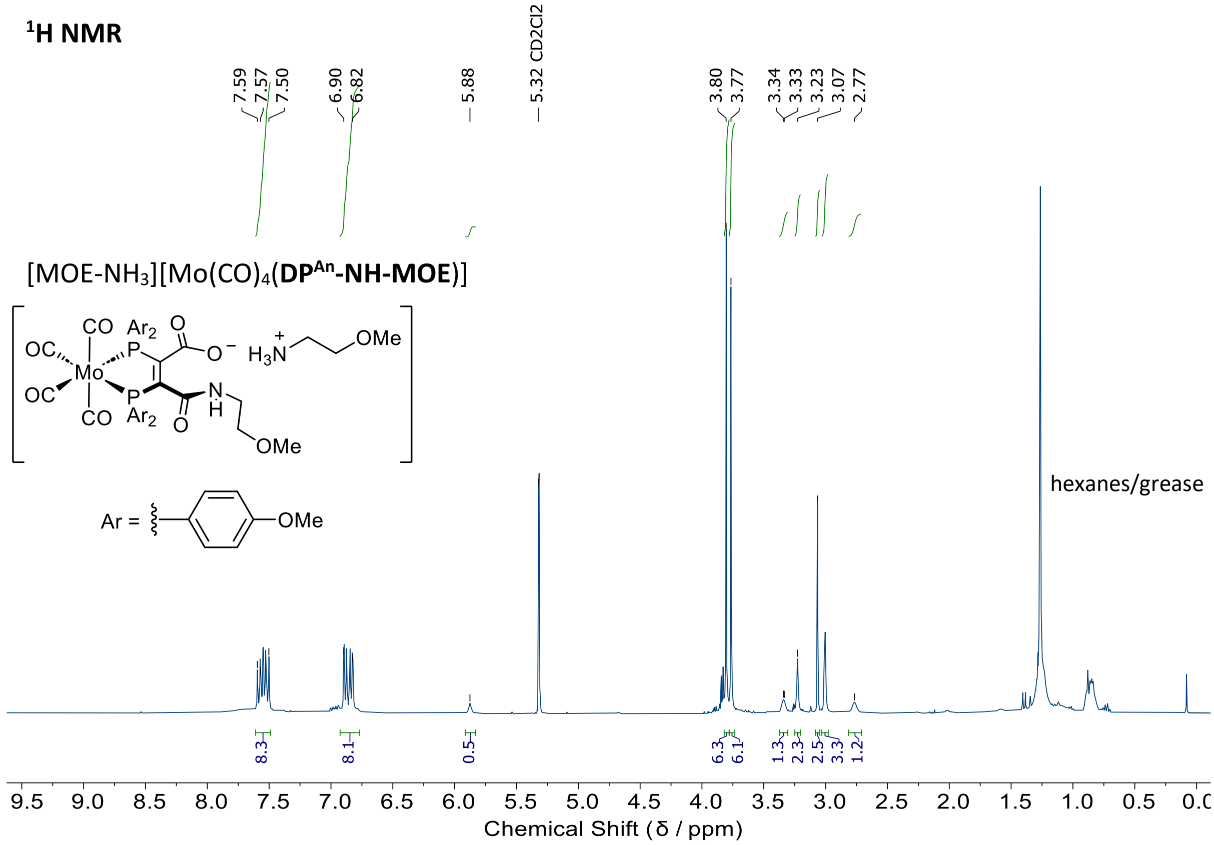
1H NMR
7.59 7.57 7.50 6.90 6.82 5.88 5.32 CD2Cl2 3.80 3.77 3.34 3.33 3.23 3.07 2.77
[MOE-NH3][Mo(CO)4(DPAn-NH-MOE)]
Ar = OMe
hexanes/grease
8.3 8.1 0.5 6.3 6.1 1.3 2.3 2.5 3.3 1.2
9.5 9.0 8.5 8.0 7.5 7.0 6.5 6.0 5.5 5.0 4.5 4.0 3.5 3.0 2.5 2.0 1.5 1.0 0.5 0.0
Chemical Shift (δ / ppm)

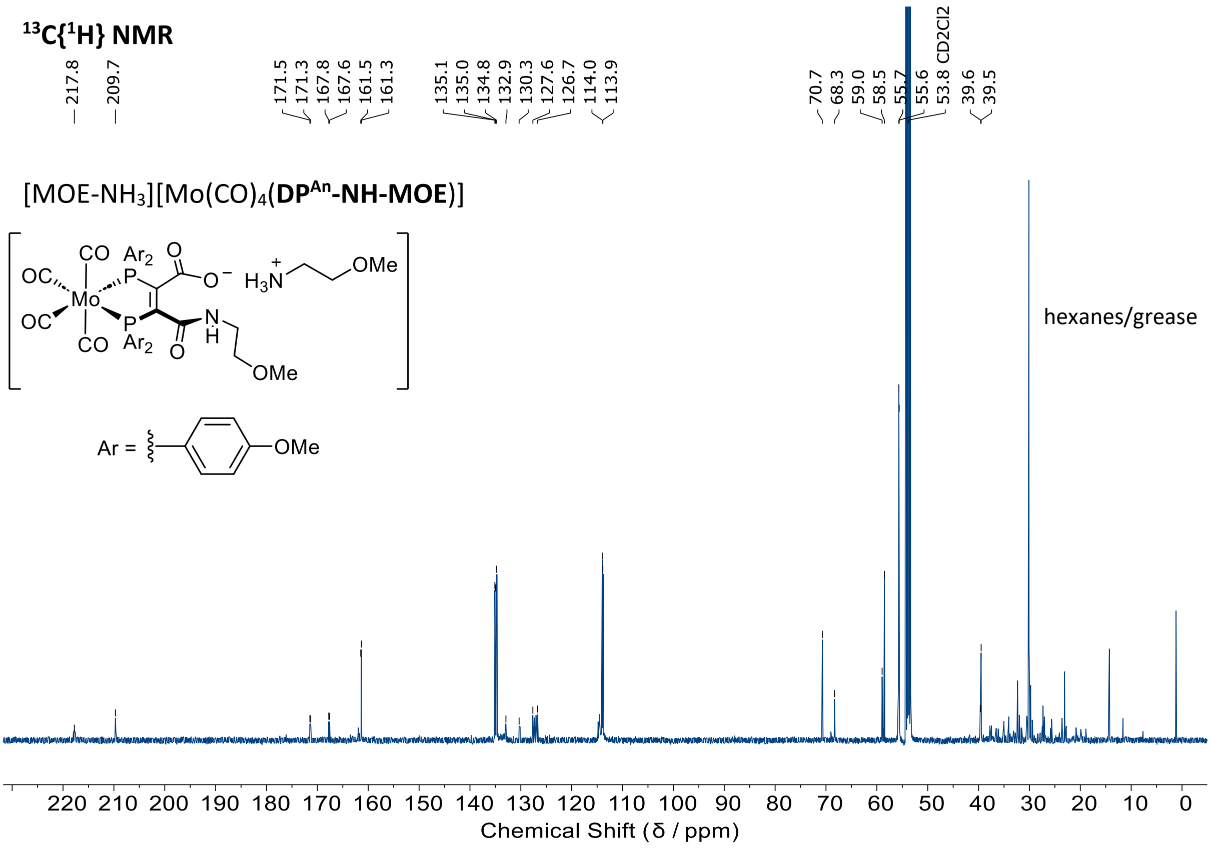
13C{1H} NMR
217.8
209.7
171.5
171.3
167.8
167.6
161.5
161.3
135.1
135.0
134.8
132.9
130.3
127.6
126.7
114.0
113.9
70.7
68.3
59.0
58.5
55.7
55.6
53.8 CD2Cl2
39.6
39.5
[MOE-NH3][Mo(CO)4(DPAn-NH-MOE)]
hexanes/grease
Ar =
OMe
220 210 200 190 180 170 160 150 140 130 120 110 100 90 80 70 60 50 40 30 20 10 0
Chemical Shift (δ / ppm)

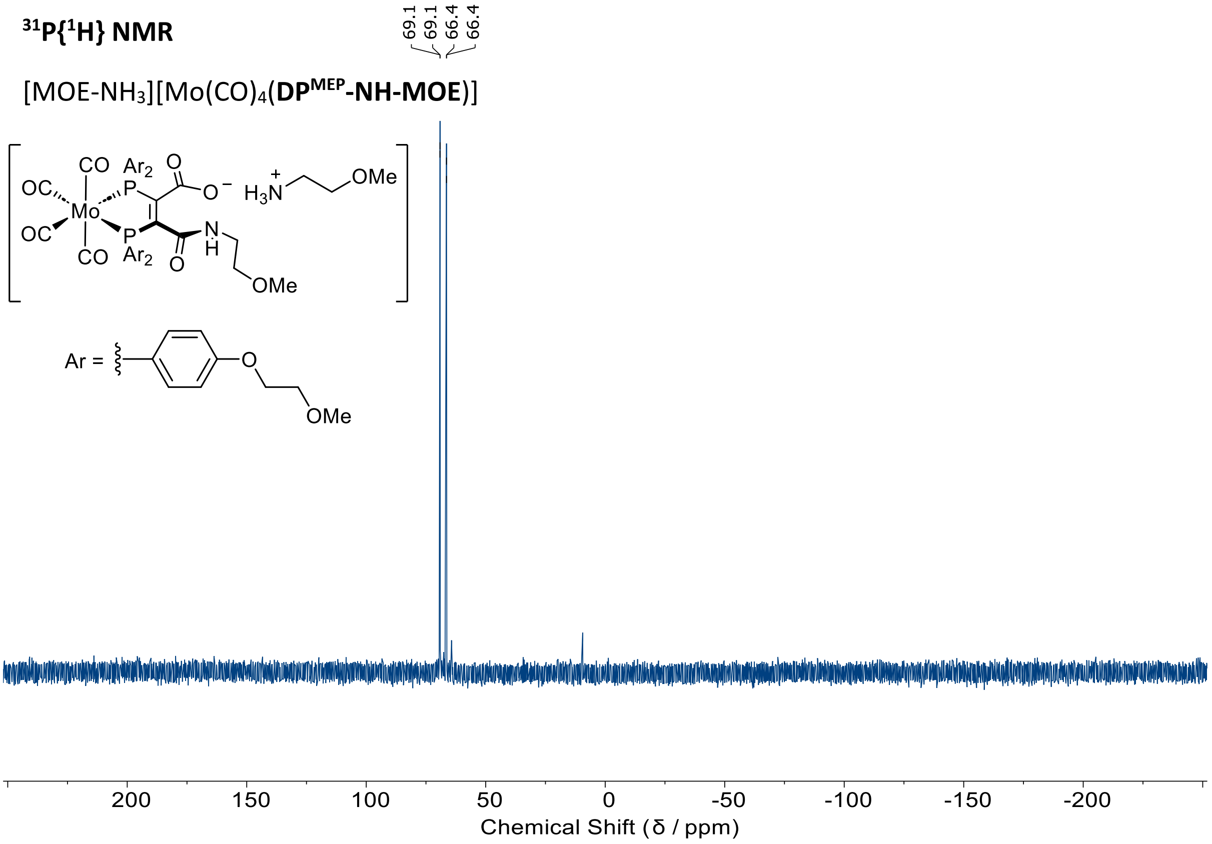
31P{1H} NMR
69.1
69.1
66.4
66.4
[MOE-NH3][Mo(CO)4(DPMEP-NH-MOE)]
Ar =
OMe
200 150 100 50 0 -50 -100 -150 -200
Chemical Shift (δ / ppm)

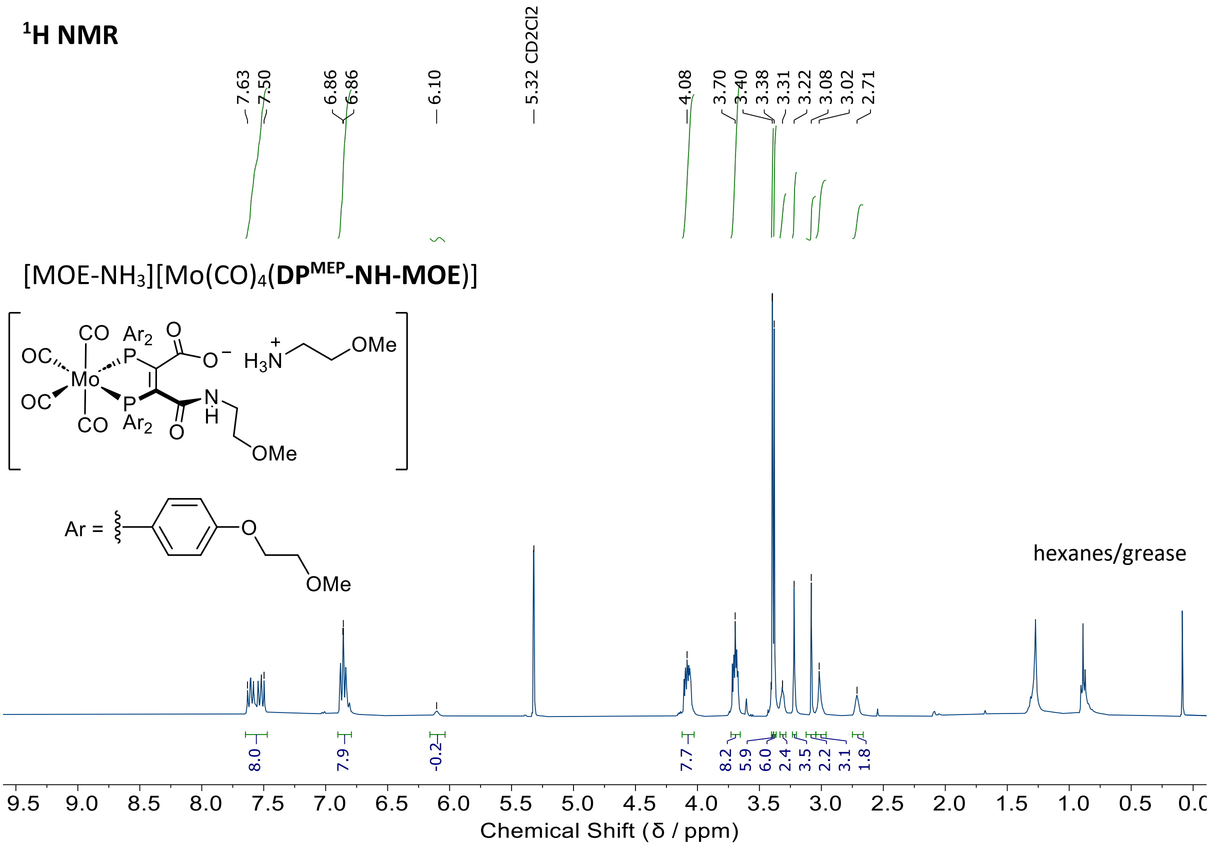
1H NMR
7.63
7.50
6.86
6.86
6.10
5.32 CD2Cl2
4.08
3.70
3.40
3.38
3.31
3.22
3.08
3.02
2.71
[MOE-NH3][Mo(CO)4(DPMEP-NH-MOE)]
CO
Ar2
OC
Mo
P
O
O−
H3N
OMe
OC
P
N
H
CO
Ar2
O
OMe
Ar =
O
OMe
hexanes/grease
8.0
7.9
-0.2
7.7
8.2
5.9
6.0
2.4
3.5
2.2
3.1
1.8
9.5 9.0 8.5 8.0 7.5 7.0 6.5 6.0 5.5 5.0 4.5 4.0 3.5 3.0 2.5 2.0 1.5 1.0 0.5
Chemical Shift (δ / ppm)

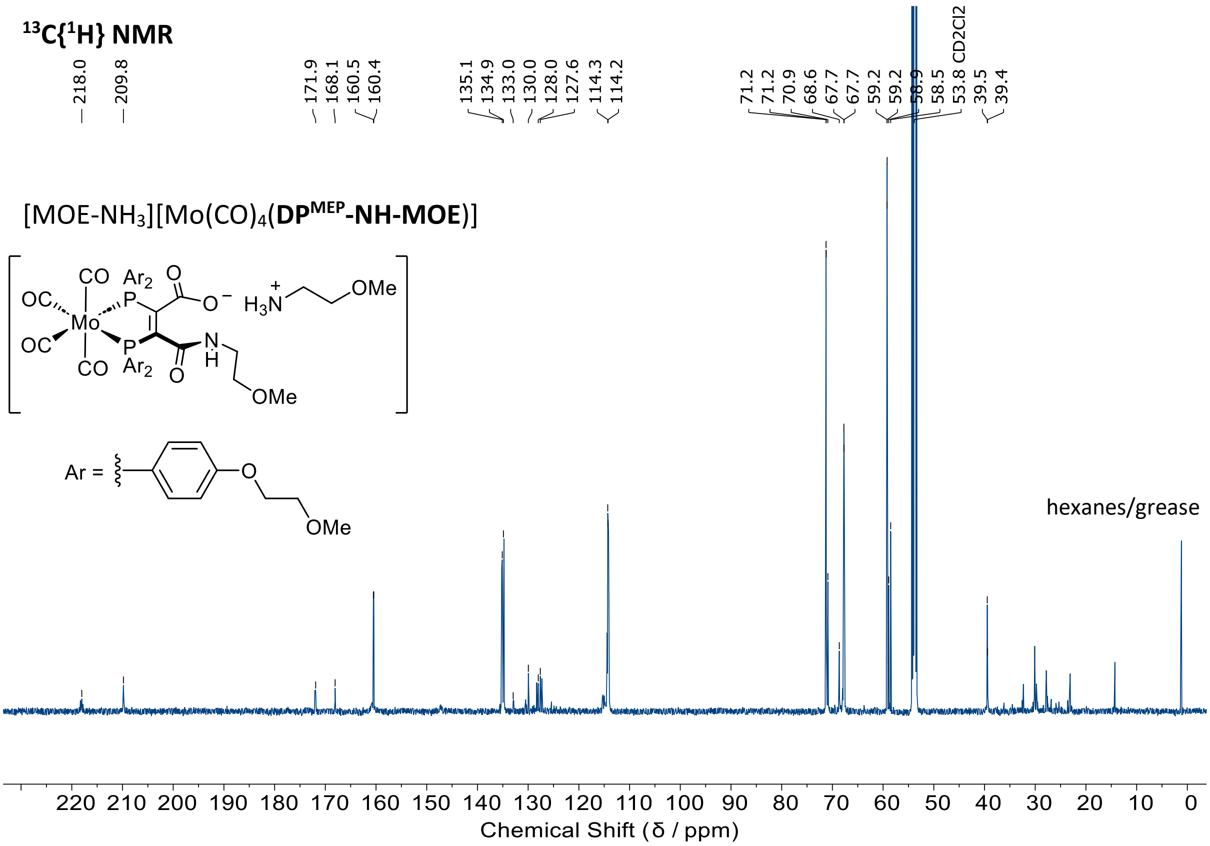
13C{1H} NMR
218.0
209.8
171.9
168.1
160.5
160.4
135.1
134.9
133.0
130.0
128.0
127.6
114.3
114.2
71.2
71.2
70.9
68.6
67.7
67.7
59.2
59.2
58.9
58.5
53.8 CD2Cl2
39.5
39.4
[MOE-NH3][Mo(CO)4(DPMEP-NH-MOE)]
CO
Ar2
OC
Mo
P
O
O−
H3N
OMe
OC
P
N
H
CO
Ar2
O
OMe
Ar =
O
OMe
hexanes/grease
220 210 200 190 180 170 160 150 140 130 120 110 100 90 80 70 60 50 40 30 20 10 0
Chemical Shift (δ / ppm)

9 References

1 I. Biancofiore, S. Cazzamalli, S. Dakhel Plaza, E. J. Donckele, J. Millul, F. Samain and E. Schmidt, *Fibroblast Activation Protein Ligands for Targeted Delivery Applications*, WO Pat., WO2021/160825A1, 2021.

2 K. Jansen, L. Heirbaut, R. Verkerk, J. D. Cheng, J. Joossens, P. Cos, L. Maes, A. M. Lambeir, I. De Meester, K. Augustyns and P. Van Der Veken, *J. Med. Chem.*, 2014, **57**, 3053–3074.

3 I. N. Hungnes, T. T. Pham, C. Rivas, J. A. Jarvis, R. E. Nuttall, S. M. Cooper, J. D. Young, P. J. Blower, P. G. Pringle and M. T. Ma, *Inorg. Chem.*, 2023, **62**, 20608–20620.

4 A. Davison, C. Orvig, H. S. Trop, M. Sohn, B. V. DePamphilis and A. G. Jones, *Inorg. Chem.*, 1980, **19**, 1988–1992.

5 M. Jauregui-Osoro, S. De Robertis, P. Halsted, S. M. Gould, Z. Yu, R. L. Paul, P. K. Marsden, A. D. Gee, A. Fenwick and P. J. Blower, *Nucl. Med. Commun.*, 2021, **42**, 1024–1038.

6 M. S. Cooper, M. T. Ma, K. Sunassee, K. P. Shaw, J. D. Williams, R. L. Paul, P. S. Donnelly and P. J. Blower, *Bioconjug. Chem.*, 2012, **23**, 1029–1039.

7 Y. Y. Yan and T. V. RajanBabu, *Org. Lett.*, 2000, **2**, 4137–4140.

8 H. Sun and C. Zhou, *Pyrazolotriazolopyrimidine Derivatives as A2A Receptor Agonist*, WO Pat., WO2020/020097A1, 2020.

9 A. Vuorinen, I. V. L. Wilkinson, M. Chatzopoulou, B. Edwards, S. E. Squire, R. J. Fairclough, N. A. Bazan, J. A. Milner, D. Conole, J. R. Donald, N. Shah, N. J. Willis, R. F. Martínez, F. X. Wilson, G. M. Wynne, S. G. Davies, K. E. Davies and A. J. Russell, *Eur. J. Med. Chem.*, 2021, **220**, 113431.

10 S. St John-Campbell, A. K. Ou and J. A. Bull, *Chem. Eur. J.*, 2018, **24**, 17838–17843.

11 T. T. Pham, I. N. Hungnes, C. Rivas, J. Cleaver, G. Firth, P. J. Blower, J. Sosabowski, G. J. R. Cook, L. Livieratos, J. D. Young, P. G. Pringle and M. T. Ma, *J. Nucl. Med.*, 2024, **65**, 1087–1094.

12 F. Kampmeier, J. D. Williams, J. Maher, G. E. Mullen and P. J. Blower, *EJNMMI Res.*, 2014, **4**, 1–10.
